# Design of an open-shell nitrogen-centered diradicaloid with tunable stimuli-responsive electronic properties

**DOI:** 10.1038/s42004-022-00747-8

**Published:** 2022-10-14

**Authors:** Bin Huang, Hao Kang, Chang-Wei Zhang, Xiao-Li Zhao, Xueliang Shi, Hai-Bo Yang

**Affiliations:** Shanghai Key Laboratory of Green Chemistry and Chemical Processes, School of Chemistry and Molecular Engineering, East China Normal University, 3663 N. Zhongshan Road, Shanghai, 200062 PR China

**Keywords:** Photochemistry, Organic molecules in materials science

## Abstract

Organic diradicaloids usually display an open-shell singlet ground state with significant singlet diradical character (*y*_0_) which endow them with intriguing physiochemical properties and wide applications. In this study, we present the design of an open-shell nitrogen-centered diradicaloid which can reversibly respond to multiple stimuli and display the tunable diradical character and chemo-physical properties. **1a** was successfully synthesized through a simple and high-yielding two-step synthetic strategy. Both experimental and calculated results indicated that **1a** displayed an open-shell singlet ground state with small singlet-triplet energy gap (Δ*E*_S−T_ = −2.311 kcal mol^−^^1^) and a modest diradical character (*y*_0_ = 0.60). Interestingly, **1a** was demonstrated to undergo reversible Lewis acid-base reaction to form acid-base adducts, which was proven to effectively tune the ground-state electronic structures of **1a** as well as its diradical character and spin density distributions. Based on this, we succeeded in devising a photoresponsive system based on **1a** and a commercially available photoacid merocyanine (MEH). We believe that our studies including the molecular design methodology and the stimuli-responsive organic diradicaloid system will open up a new way to develop organic diradicaloids with tunable properties and even intelligent-responsive diradicaloid-based materials.

## Abstract (web summary)

The electronic structures and open-shell diradical character of organic diradicaloids endow them with potentially useful optical, electronic and magnetic properties. Here, an open-shell nitrogen-centered diradicaloid is reported and shown to readily react with Lewis and Brønsted acids to form acid-base adducts, allowing for tuning of ground-state electronic structure, diradical character and spin density distribution.

## Introduction

**Fig. 1:**
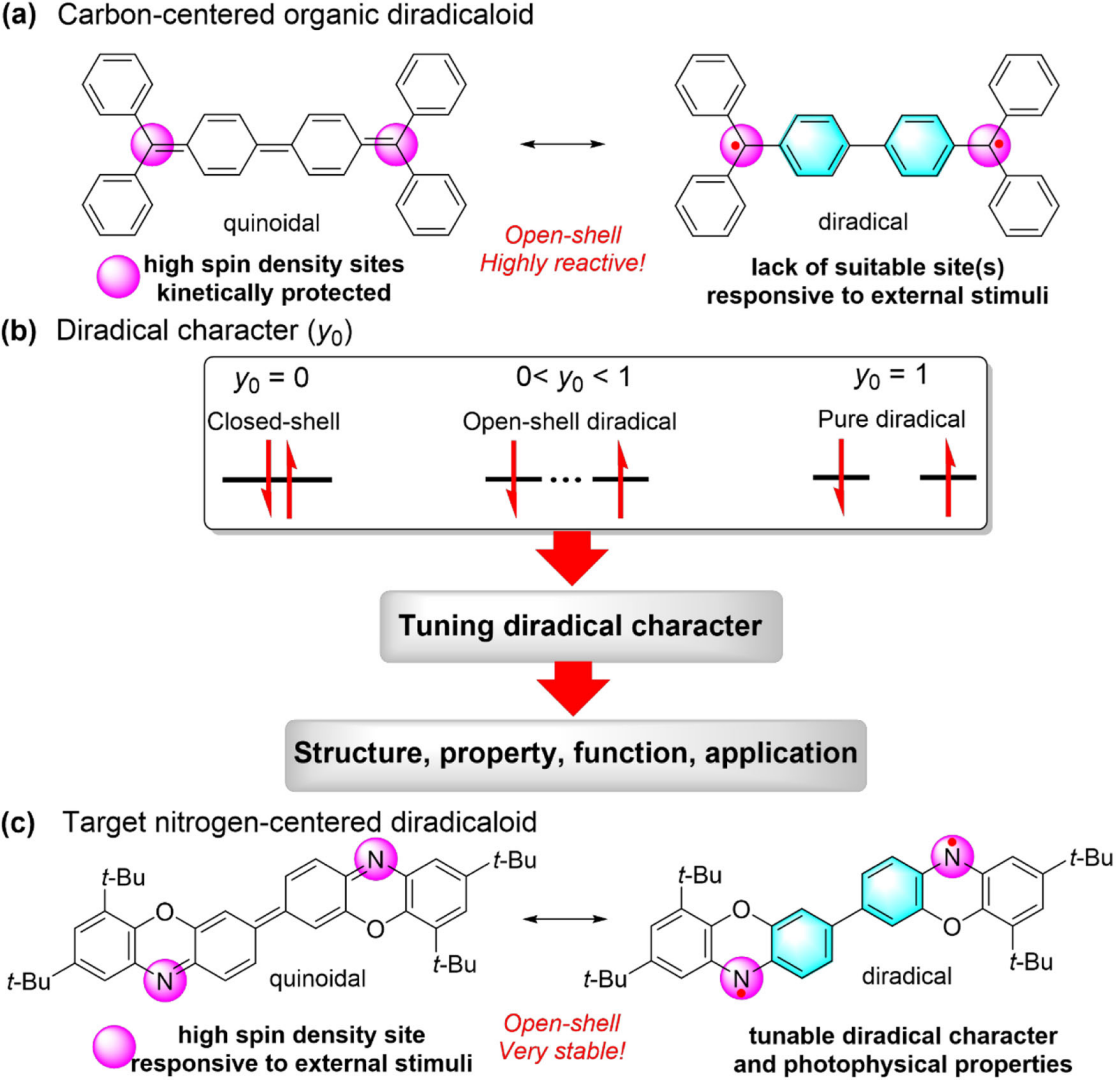
Design principle of this study. **a** Classical carbon-centered organic diradicaloid of Chichibabin’s hydrocarbon and its two characteristic resonance structures. **b** Diradical character (*y*_0_) of organic diradicaloids. **c** Target nitrogen-centered diradicaloid.

Organic diradicaloids refer to a special kind of molecules with two electrons occupying two quasi-degenerate orbitals, and can be represented by two characteristic resonance structures between closed-shell quinoidal and pure open-shell (diradical) forms^1,2^. A great number of studies have demonstrated that many organic diradicaloids, such as the most famous Chichibabin’s hydrocarbon (Fig. 1a), display an open-shell singlet ground state with significant singlet diradical character (*y*_0_). The unique electronic structures and open-shell diradical character usually endows organic diradicaloids with intriguing properties such as small energy gap with long absorption wavelength^3^, ambipolar transport characteristics^4–6^, distinct magnetic behavior^7^, and special chemical reactivity^8,9^. Therefore, tuning the diradical character is crucially important because the magnitude of diradical character not only dictates the chemical reactivity, magnetic behavior and photophysical properties of diradicaloids but also determines their functions and applications, especially optoelectronic and spintronic applications (Fig. 1b).

Numerous reports in the literature have successfully demonstrated that many internal factors such as the quinoidal conjugation length^10,11^, fusion motifs of aromatic rings^12–15^, planarity of molecules^16^, incorporation of heteroatoms^17–19^ and so on could effectively influence or control their ground-state electronic structures and diradical characters^20–22^. Recently, a few of reports have disclosed that some external stimuli, particularly heat, could exert a significant effect on the diradical characters of organic diradicaloids as well as their magnetic behavior and photophysical properties^23–29^. For example, Dou and co-workers succeeded in the dynamic modulation of the diradical character based on a family of boron-containing organic diradicaloids by Lewis acid-base coordination^23,30–33^. However, the examples of tuning the diradical character of organic diradicaloids via external stimuli are still very conservative compared with internal factors. This is largely due to the fact that most organic diradicaloids are π-conjugated polycyclic hydrocarbons containing only carbon and hydrogen atoms, thus lacking the suitable site(s) responsible for external stimuli. In addition, the sites with higher spin density (the most reactive sites) in most organic diradicaloids are normally kinetically protected because of the stability concerns^34–38^, which also makes the response to external stimuli more difficult. In this scenario, we speculate that varying the chemical environment of sites with high radical density in the organic diradicaloid can more effectively influence its ground-state electronic structures (Fig. 1). In addition, reversibly tuning the diradical character of organic diradicaloids with multiple external stimuli still remains a great challenge and has not yet been realized thus far.

Herein we report the de novo design and synthesis of an open-shell organic diradicaloid molecule **1a**, and the general molecular design concept is based on the following considerations (Fig. 1c): (a) target molecule **1a** can be conceptually considered as a nitrogen-centered diradicaloid, wherein significant spin density is expected on the two bare aminyl nitrogen atoms in its open-shell resonance form; (b) replacement of the carbon radical center with the electronegative and Lewis basic aminyl radical is essential to improving the stability of **1a** as well as the occurrence of the Lewis acid-base reaction^39–41^; (c) moreover, the *tert*-butyl groups at both ends of **1a** not only improve its solubility but also effectively protect the adjacent reactive positions of carbon bearing some spin densities (*vide infra*). The systematical studies revealed that **1a** displayed an open-shell singlet ground state with small singlet-triplet energy gap (Δ*E*_S−T_ = −2.311 kcal mol^−^^1^) and a medium diradical character (*y*_0_ = 0.60). Moreover, based on this rational molecular design, **1a** was demonstrated to readily undergo the Lewis acid-base reaction with the Lewis acid, Brønsted acid and photoacid to form acid-base adducts, which was accompanied by the significant changes in both photophysical and magnetic properties. Therefore, this work provides a convenient synthesis along with the characterizations of an open-shell organic diradicaloid with tunable and responsive electronic properties and diradical character for the first time to the best of our knowledge.

## Results

### Molecular synthesis and characterization

**Fig. 2:**
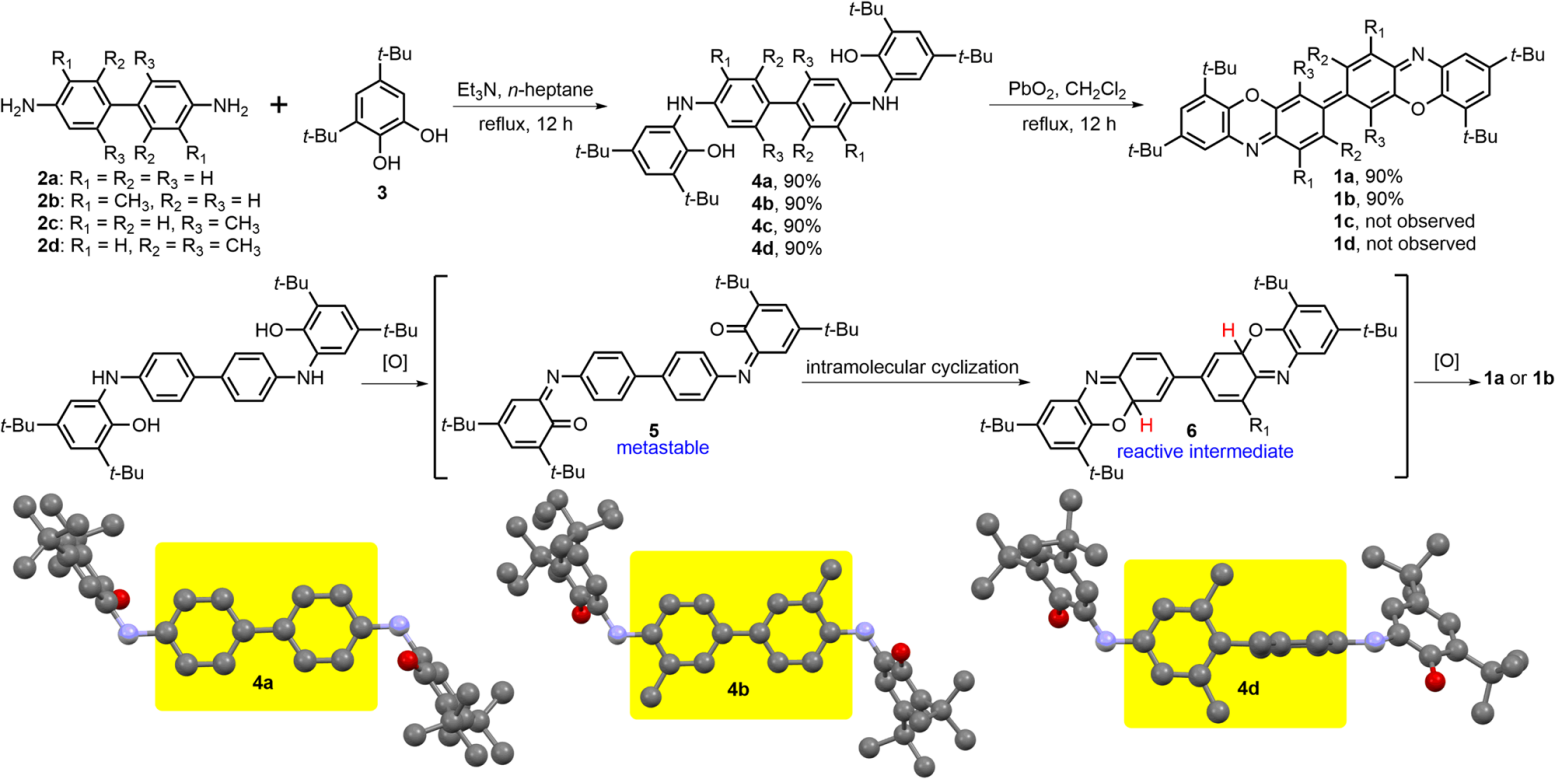
Two-step synthesis protocol for the preparation of the target nitrogen-centered diradicaloid without the need for column chromatography purification. Synthesis of **1a** and **1b** and the proposed cascade reaction sequence. The single crystal structures of the condensation products **4a**, **4b** and **4d** are shown.

The prevalent strategies for preparing organic diradicaloids usually require multistep synthesis strategies, strict air-free conditions, and tedious purification processes. In contrast, as shown in Fig. 2, diradicaloid **1a** can be simply and efficiently synthesized in two steps involving a facile condensation reaction between benzidine **2a** and two equivalent amounts of 3,5-di-*tert*-butyl catechol **3**, and a cascade reaction in the presence of lead (IV) oxide, without any purification by column chromatography. The cascade reaction sequence might consist of an oxidation reaction, an intramolecular cyclization^42^ and a subsequent oxidative aromatization step, accompanied by the generation of unstable quinone imines **5** and a highly reactive dihydro intermediate **6**. The scope of the substituted benzidine substrates was surveyed and the preliminary results indicated that the substrate scope of this cascade reaction was restricted to benzidine **2a** and 3,3’-dimethylbenzidine **2b**, e.g., all attempts to synthesize diradicaloids **1c** and **1d** were unsuccessful (for details see the Supplementary Information (SI), Synthesis and characterization section). X-ray single-crystal analyses revealed the distinct conformations of the condensation products, namely, **4a** and **4b** were planar while **4d** was highly twisted (Supplementary Data 5, Data 6 and Data 7). Therefore, we speculated that diradicaloids **1c** and **1d** might be highly reactive because of their twisted structures which was supported by the simulations (Supplementary Fig. 25). As a result, the highly twisted structure led to a higher diradical character of **1d**, which was up to 0.977 (Table 7). The resultant diradicaloids **1a** and **1b** were very stable and thoroughly characterized by ^1^H NMR, HR-MS measurements and X-ray crystallographic analysis (*vide infra*). Because **1a** and **1b** are expected to display similar properties, we focus on the investigation of **1a** to avoid redundancy.

### Photophysical and electrochemical properties

**Fig. 3:**
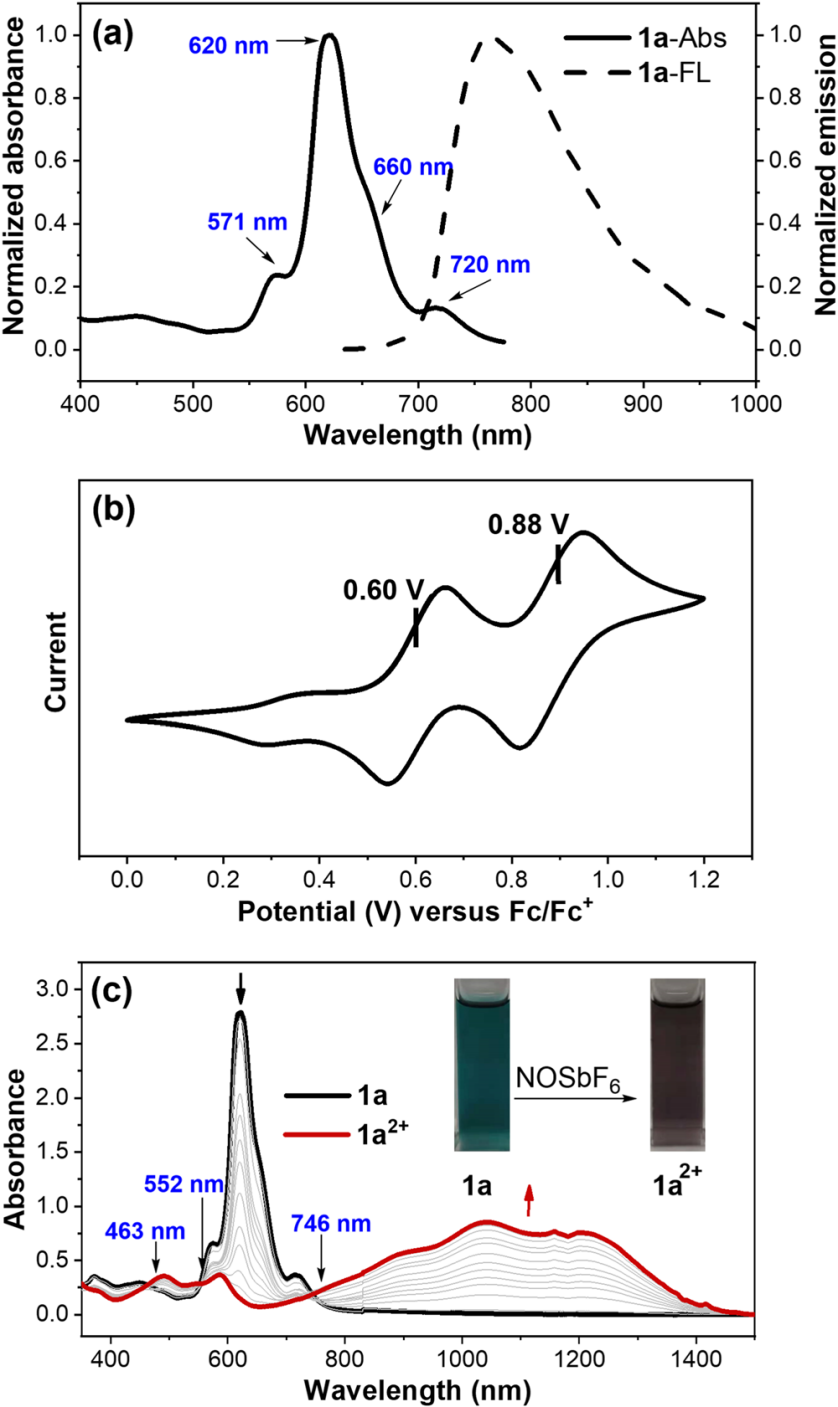
Photophysical and electrochemical properties of 1a. **a** Normalized absorption and emission spectrum of **1a**. **b** Cyclic voltammogram of **1a** in DCM with 0.1 M Bu_4_NPF_6_ as the supporting electrolyte, Ag/AgCl as the reference electrode, and a Pt wire as the counter electrode and a scan rate at 20 mV/s. **c** Absorption titration for the chemical oxidation of **1a** using NOSbF_6_ as oxidant. Inserted are the photos of the solutions.

The UV-vis-NIR absorption spectrum of **1a** featured four main bands from the far-red to near-infrared region with maxima at 571, 620, 660, and 720 nm, resulting in its extensive green color (Fig. 3a). Notably, the weak, longest-wavelength absorption band is the characteristic of the open-shell singlet diradicaloids mainly due to their double-excitation nature (HOMO,HOMO → LUMO,LUMO)^43^. The absorption profile of **1a** was also very similar to some other representative open-shell diradicaloids, such as quinoidal oligothiophenes^44–46^, zethrenes^47–50^, teranthene^51^, bisphenalenyls^52–54^ and so on^55^, further indicating its intrinsic open-shell diradical character. When we surveyed its luminescent property, an interesting observation was that **1a** unexpectedly exhibited near infrared fluorescence (Fig. 3a), which is obviously different from the nonemissive nature of most open-shell diradicaloids. The near-infrared fluorescence quantum yield of **1a** was estimated to be approximately ~4% by the absolute integrating sphere method (Supplementary Fig. 4). Recently, organic radicals, typical monoradicals, have emerged as increasingly important promising luminescent materials for practical applications, while luminescent diradicals are very rare^48,56–61^. Therefore, to the best of our knowledge, **1a** might be the first example of emissive open-shell organic diradicaloid with substantial fluorescence quantum yield.

The electrochemical property of **1a** was investigated by performing cyclic voltammetry (CV) in a dry DCM solution (Fig. 3b and Supplementary methods). **1a** exhibited two reversible oxidation waves with halfwave potentials (*E*_1/2_) of 0.60 and 0.88 V (vs. Fc/Fc^+^). The HOMO energy level was then estimated to be −5.31 eV based on the onset potential of the first oxidation wave. It is important to note that no reduction wave was observed, which is in contrast to the electrochemical amphoteric redox behavior of most organic diradicaloids. The high-lying HOMO energy level and unobserved reduction wave might be indicative of the highly electron-rich nature of **1a**, and thus making it possible to serve as a Lewis base. The two reversible and well-separated oxidation waves of **1a** implied that its radical cation **1a**^**•+**^ and dication **1a**^**2+**^ species were theoretically stable and accessible. However, the UV-vis-NIR absorption titration for the chemical oxidation of **1a** revealed the exclusive generation of dication species **1a**^**2+**^ as demonstrated by the presence of the isosbestic points (Fig. 3c). Similarly, during the chemical oxidation of **1a** and crystal growing process, single crystals of **1a**^**2+**^ always formed when the oxidation compound precipitated out, even with the addition of less than one equivalent of NOSbF_6_ (*vide infra*). Thus, this finding provided accumulating evidence that only the dication species was accessible when **1a** underwent chemical oxidation although it displayed two reversible oxidation waves.

### X-ray crystallographic analysis

**Fig. 4:**
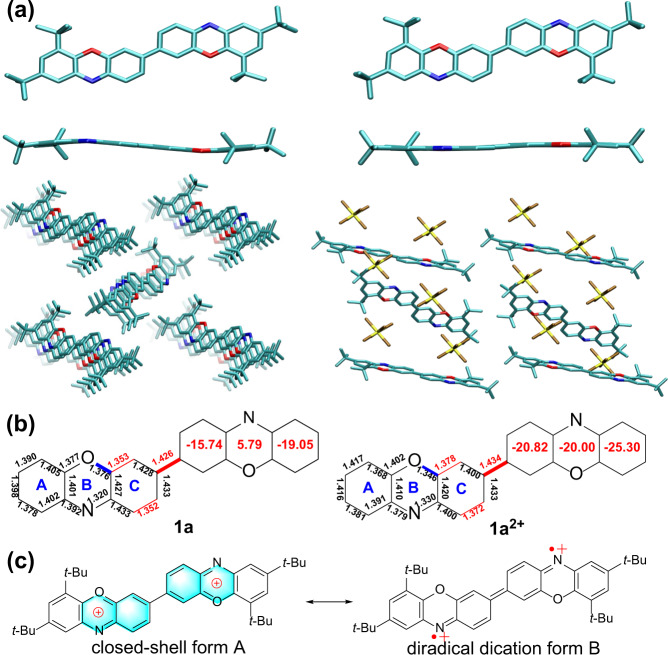
Single crystal structures of 1a and 1a^2+^. Single crystal structures including top view, side view and packing modes (**a**), selected bond lengths (**b**) of **1a** and **1a**^**2+**^, and resonance structures of **1a**^**2+**^ (**c**). The red numbers in the rings denote the calculated NICS(1)_ZZ_ values.

Single crystals of **1a** and **1a**^**2+**^, suitable for X-ray crystallography analysis, were successfully grown and analyzed (Supplementary Data 2, Data 3 and Supplementary methods). We found only the *trans* isomer of **1a** formed, due to its lower energy compared with *cis* isomer **1a’** (Supplementary Fig. 26)^62^. The molecular geometry of **1a** was slightly bent with dihedral angle nearly 171.6°, mainly due to the lone pairs of electrons on the oxygen and nitrogen atoms. In addition, **1a** adopted 1D columnar packing along the *a* axis with offset face-to-face π–π stacking (~3.7 Å) and the adjacent π-stacks were connected through C − H⋅⋅⋅H − C interactions (~2.50 Å) (Fig. 4a). Therefore, the crystal packing effects might also account for the favorable *trans* isomer observed in the solid state. Bond length analysis was performed to better analyze the ground-state molecular geometry of **1a** (Fig. 4b). A large bond length alternation (BLA) was observed for the central bridged benzene ring (ring C) in **1a** (~0.078 Å), which was much larger than that of the Chichibabin’s hydrocarbon (~0.052 Å)^63^, indicating its dominant quinoidal structure at a low temperature (100 K). However, the bond length for the central C-C bond (highlighted in bold red in Fig. 4b) in **1a** was determined to be approximately 1.426 Å, which was longer than that of a double bond (1.34 Å) and slightly shorter than the typical biphenyl single bond (1.48 Å), suggesting the nonnegligible contribution of the diradical resonance form to the ground state. Single crystals of **1b** were also successfully obtained, and detailed structural information, such as its planarity and bond length analysis, was quite similar to that of **1a** (Supplementary Figs. 8 and 9, Supplementary Data 4, Supplementary Table 1), therefore, the analysis of its crystal will not be covered again here.

The chemical oxidation of **1a** resulted in a highly symmetrical molecule bearing two SbF_6_^-^ counteranions, clearly confirming the formation of **1a**^**2+**^. **1a**^**2+**^ was essentially planar with a dihedral angle of ~180°, indicating an electronic structure that was distinct from that of neutral **1a** (Fig. 4a). No intermolecular π–π stacking was observed for **1a**^**2+**^, probably due to the potential coulomb repulsion between the two positively charged molecules. Regarding the ground-state geometry of **1a**^**2+**^, it raised the obvious question: what’s the major resonance contributor of **1a** between the closed-shell form A and diradical dication form B (Fig. 4c)? Bond length analysis illustrated that **1a**^**2+**^ showed a smaller BLA (~0.038 Å) than neutral **1a**. Moreover, the bond length for the central C-C bond (highlighted in bold red in Fig. 4b) in **1a**^**2+**^ was measured to be ~1.434 Å, which was longer than that of **1a**. More significantly, the C-O (highlighted in bold blue in Fig. 4b) bond length decreased from 1.376 Å in **1a** to 1.346 Å in **1a**^**2+**^, likely suggesting that the latter displayed partial double-bond characteristics. The above bond length analysis results of **1a**^**2+**^ basically elucidated that the closed-shell form A might dominate its ground-state, which was also consistent with its sharp NMR peaks and unobserved EPR signal (*vide infra*). Specifically, because the quinoidal benzidine unit intrinsically tended to recover the aromaticity of the two benzenoid rings, **1a** was thus prone to the two-electron oxidation process rather than the one-electron oxidation process, thereby leading to the exclusive formation of dication **1a**^2+^^64,65^.

### Magnetic properties

**Fig. 5:**
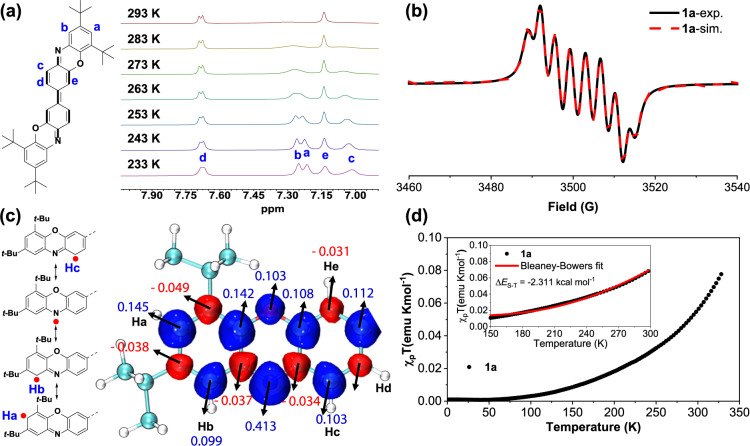
Magnetic properties of 1a. **a** VT ^1^H NMR spectra (aromatic region) of **1a** in CD_2_Cl_2_ and assignment of all aromatic protons. **b** EPR spectrum of **1a** in CH_2_Cl_2_ solution measured at room temperature and its simulation. **c** The typical resonance forms of **1a** and its calculated spin density distributions. For clarity, only one-half of **1a** is shown. **d** *χ*T–T plot for the powder of **1a** in SQUID measurement. The inset shows the best fitting plots (red solid line) obtained with the Bleaney-Bowers equation. *χ*_p_ = *χ*_mol_-*χ*_d_, *χ*_d_: diamagnetic susceptibility (*χ*_d_ of **1a**: ~ −425 × 10^−^^6 ^emu mol^−^^1^).

Variable temperature (VT) ^1^H NMR spectra of **1a** were recorded in CD_2_Cl_2_ (Fig. 5a and Supplementary methods). The resonances of all the protons were successfully assigned to the structure of **1a** based on through-space correlations by 1D and 2D NOESY experiments at 233 K (Supplementary Fig. 1 and Supplementary methods). As expected, significant signal broadening was observed for **1a** even at room temperature mainly due to the presence of thermally populated triplet species caused by the small singlet-triplet energy gap ∆*E*_S-T_, further confirming that **1a** displayed an open-shell singlet ground state. The VT NMR spectra of **1a** showed a progressive sharpening of some resonances in both aromatic and aliphatic regions when the temperature was gradually lowered to 233 K (Fig. 5a and Supplementary Fig. 2). In contrast, the intensity of the aromatic protons of *d* and *e* exhibited only a small temperature dependence from 293 to 233 K. We deduced that the spin densities of **1a** were mainly delocalized on the N atoms as well as the *ortho* and *para* carbon atoms, which could be depicted by its multiple resonance structures (Fig. 5c). According to the spin density calculations (Fig. 5c), in addition to the obvious spin density of the N atom, the alternating spin densities of the outermost two benzene rings and the quinoidal benzidine unit were also observed, thus providing support for our hypothesis. As a result, the protons on or around the carbons with high spin density were subject to a more obvious paramagnetic environment, leading to the more obvious broadening of *a*, *b* and *c* protons than that of *d* and *e*.

The EPR study of **1a** provided more direct evidence of the presence of radical species both in the solution and solid state (Fig. 5b and Supplementary Fig. 5). The solution of **1a** displayed a weak EPR signal mainly due to the relatively low spin concentration of its dilute solution. Nevertheless, its solution-state EPR spectrum exhibited a well-resolved octet hyperfine structure, mainly stemming from the hyperfine interactions of the N and multiple H nuclei based on the simulations. Specifically, the simulated spectrum satisfactorily agreed with the experimentally observed spectrum, yielding simulated hyperfine coupling constants (hfccs) of 7.408 G (*A*_N_), and 3.777 G, 2.853 G and 2.688 G, corresponding to three proton hyperfine couplings, and a *g*-value of 2.0024, which was consistent with the aforementioned calculated spin density maps and VT NMR results. **1a** also displayed three broad EPR lines in the solid state with enhanced intensity compared with that in solution because of the increased spin concentration (Supplementary Fig. 5). The forbidden half-field transition of **1a** was not observed, mainly because the concentration of the thermally populated triplet species was relatively low, and this was the common phenomenon in many diradicaloids with small to moderate diradical character^47–50,66–73^. In contrast to **1a**, **1a**^**2+**^ was EPR silent and showed well-resolved sharp peaks in the ^1^H NMR spectrum at room temperature (Supplementary Figs. 6 and 7), which was consistent with its inferred closed-shell form A (Fig. 4c). Nucleus independent chemical shift (NICS) calculations were then conducted to clarify the difference between neutral **1a** and dication **1a**^**2+**^ regarding their aromaticity (Fig. 4b). The obviously negative NICS value of ring C and small positive value of ring B in **1a** suggested the significant aromaticity of the central bridged benzene ring but very weak antiaromaticity of the phenoxazine ring, further supporting its nonnegligible contribution of the diradical resonance form to the ground state (Fig. 4b). Compared to **1a**, the NICS values for central phenoxazine ring B and ring C in **1a**^**2+**^ became more negative, indicating that the oxidation process could increase the aromaticity of dication **1a**^**2+**^, consistent with its highly planar structure and the proposed closed-shell form A based on X-ray crystallographic analysis.

The magnetic property of **1a** was further investigated by the superconducting quantum interference device (SQUID) magnetometer (Fig. 5d and Supplementary methods). SQUID measurement of the solid of **1a** showed a very small *χ*_p_T value of approximately 0.07 emu Kmol^-1^ at 300 K. This is very common among most reported open-shell diradicaloids with moderate diradical character, in which a small quantity of thermally excited triplet species account for their paramagnetic property. **1a** exhibited very weak antiferromagnetic behavior, and a magnetic susceptibility enhancement was observed with increasing temperature. The singlet-triplet energy gap (∆*E*_S-T_ or 2 *J*/*k*_B_) of **1a** was then determined to be approximately ~−2.311 kcal mol^−^^1^ by fitting the susceptibility data using the Bleaney-Bowers equation (Supplementary methods)^74^. Thus, **1a** was arguably an open-shell diradicaloid, and a thermally excited triplet state was accessible at room temperature due to its relatively small ∆*E*_S-T_. Density functional theory (DFT) calculations at the CAM-B3LYP-D3(BJ)/def2-TZVP level were further conducted to elucidate the ground-state electronic structure of **1a** (Supplementary Fig. 28 and Supplementary Table 6). The electronic structure was analyzed using Multiwfn^75^. The results showed that **1a** favored an open-shell singlet ground state with a medium singlet diradical character (*y*_0_ = 0.55). The open-shell singlet diradical state was estimated to be 2.10 and 10.22 kcal mol^-1^ lower than the triplet diradical and closed-shell states, respectively. Thus, the calculation results were in good agreement with the featured open-shell electronic absorption, X-ray crystallographic analysis and magnetic measurements of **1a**, all of which proved that it had an open-shell singlet ground state.

### Acid-base stimuli-responsive properties

**Fig. 6:**
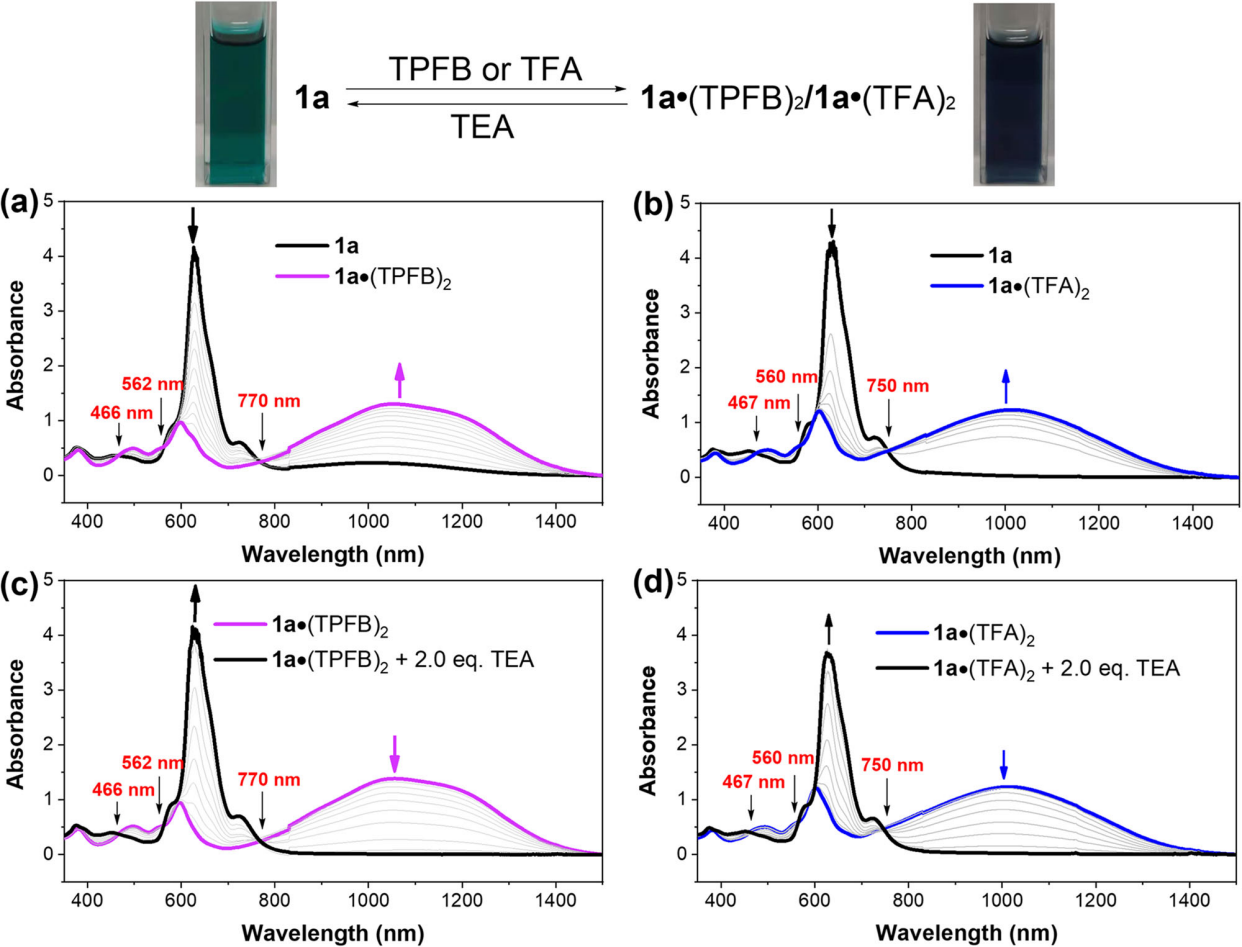
UV–vis–NIR absorption changes of 1a in the reversible Lewis acid-base reaction. **a** Schematic illustration of the reversible Lewis acid-base reaction between **1a** and corresponding acids. **b** Absorption spectral changes during the titration of **1a** with TPFB (left) and TFA (right). **c** Absorption spectral changes during the titration of **1a**•(TPFB)_2_ (left) and **1a**•(TFA)_2_ (right) with TEA.

As mentioned above, **1a**, consisting of electron-rich nitrogen centers, is anticipated to act as a Lewis base and undergo reversible acid-base reactions (Fig. 6a). UV-vis-NIR absorption spectrophotometric titrations between **1a** and Lewis acid tris(pentafluorophenyl)borane (TPFB) or Brønsted acid trifluoroacetic acid (TFA) proved the intrinsic Lewis basicity of **1a**, thereby permitting the Lewis acid-base adduct approach to tuning its diradical character. Upon the addition of TPFB to **1a**, an obvious decrease in the intensity of its maximum absorption between 600 and 700 nm was observed, accompanied by the emergence of a weak absorption band at 600 nm and a new broad long-wavelength absorption in the range of 700–1400 nm (Fig. 6b). In addition, saturation of the absorbance at a nearly 2:1 molar ratio of TPFB to **1a** was observed. This was concomitant with a set of isosbestic points at 463, 560, 770 nm, suggesting the formation of the Lewis acid-base adduct of **1a**•(TPFB)_2_. A similar spectral change was observed when titrating **1a** with TFA, yielding the acid-base adduct **1a**•(TFA)_2_, which resembled **1a**•(TPFB)_2_ in the absorption profile (Fig. 6b). Thus, both Lewis acid and Brønsted acid produced similar effects on the photophysical properties of **1a** after the formation of acid-base adducts. Similar to the oxidation process, the intermediate of the 1:1 acid-base adduct was not observed, which might be desirable to recover more aromaticity. Titration of a **1a** with a weaker acid (glacial acetic acid (AA)) also indicated that no mono-protonated species formed during this acid-base reaction (Supplementary Fig. 20). Interestingly, the acid-base adducts could almost completely be restored to **1a** after the addition of two equivalents of a stronger Lewis base triethylamine (TEA), indicating that this Lewis acid-base adduct approach was highly reversible (Fig. 6 and Supplementary Fig. 20).

**Fig. 7:**
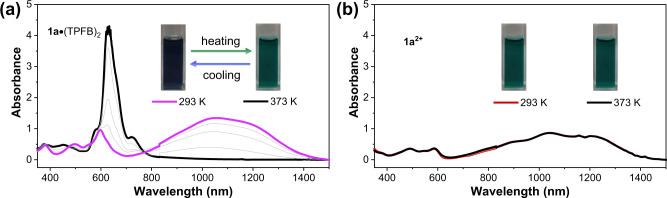
Variable temperature UV-vis-NIR absorption of acid-base adducts and dications. Variable temperature absorption of (**a**) **1a**•(TPFB)_2_ and (**b**) **1a**^**2+**^ in C_2_H_2_Cl_4_ (14 μM).

One may argue that the dication species **1a**^**2+**^ and the acid-base adducts more or less resembled each other in their UV-vis-NIR absorption profiles. However, by comparing and contrasting the UV data, the detailed absorption characteristics, especially the fine structures in the UV-vis-NIR absorption spectra, between **1a**^**2+**^ and both acid-base adducts were different (Supplementary Fig. 14, highlight in shaded region). In addition, their solution also displayed distinct color that **1a**^**2+**^ was dark brown while the acid-base adducts were dark blue. Therefore, their distinct UV data clearly implied that **1a**^**2+**^ and the acid-base adducts exhibited completely different electronic structures. Moreover, variable temperature (293 K to 373 K) UV-vis-NIR absorption of **1a**•(TPFB)_2_ and **1a**^**2+**^ indicated that the former could undergo reversible dissociation while the latter exhibited no response to temperature (Fig. 7)^32,76^. Specifically, the acid-base adduct **1a**•(TPFB)_2_ dissociated into free **1a** progressively with increasing temperature, accompanied by an obvious color change from original dark blue at room temperature to blue color at a higher temperature. This intrinsic temperature-dependent photophysical behavior of **1a**•(TPFB)_2_ suggested that the reaction between **1a** and TPFB was simple Lewis acid-base reaction while the oxidation reaction was not involved^77^.

**Fig. 8:**
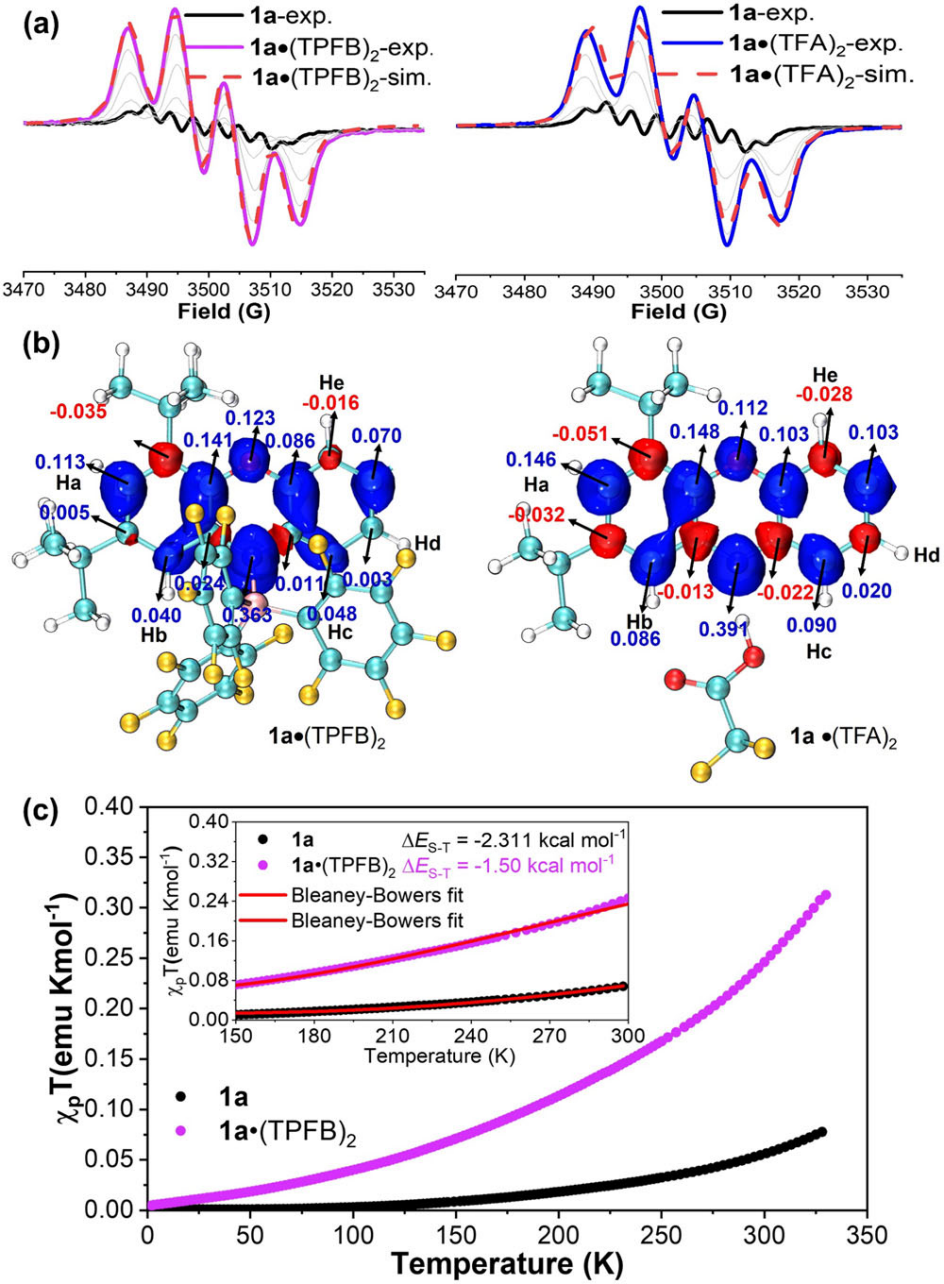
Magnetic property changes of 1a in the reversible Lewis acid-base reaction. **a** EPR spectral changes during the titration of **1a** with TPFB (left) and TFA (right), and EPR simulation of **1a**•(TPFB)_2_ and **1a**•(TFA)_2_. **b** Calculated spin density distributions of **1a**•(TPFB)_2_ (left) and **1a**•(TFA)_2_ (right). For clarity, only one-half of molecule is shown. **c** Comparison of *χ*T–T plot for **1a** and **1a**•(TPFB)_2_. The inset shows the best fitting plots (red solid line) and singlet-triplet energy gap obtained with the Bleaney-Bowers equation. *χ*_p_ = *χ*_mol_-*χ*_d_, *χ*_d_: diamagnetic susceptibility (*χ*_d_ of **1a**: ~ −425 × 10^−^^6 ^emu mol^-1^, *χ*_d_ of **1a**•(TPFB)_2_: ~ −846 × 10^-6 ^emu mol^-1^).

The magnetic properties of **1a** also changed significantly upon the addition of acids. The EPR signal intensity of pristine **1a** progressively increased with increasing acid concentration and then reached a saturation point when nearly two equivalent amounts of TFA or TPFB were added (Fig. 8a). The EPR spectral changes for both titrants were very similar, and the resultant acid-base adducts **1a**•(TPFB)_2_ and **1a**•(TFA)_2_ resembled each other in their EPR patterns. Thus, the EPR titration results also supported the formation of acid-base adducts in a 2:1 molar ratio, which was consistent with the absorption titration data. In addition, another notable change in the EPR titration spectra was the distinct hyperfine splitting patterns between **1a** and the acid-base adducts. Theoretical calculations demystified that the characteristic quartet hyperfine splitting patterns of the acid-base adducts originated from the distinct spin density distributions after **1a** coordinated with acid, *i.e*., the spin densities on the carbons linked to Hb and Hc diminished significantly (Fig. 8b). Therefore, the EPR spectra could be roughly simulated based on their calculated spin density distributions (Fig. 8a, b), wherein the four-line splitting of both **1a**•(TFA)_2_ and **1a**•(TPFB)_2_ occurred mainly due to the isotropic ^14^N hyperfine coupling and proton (Ha, Hb and Hc) superhyperfine splitting. Moreover, the EPR profile could spring back to its original shape and intensity after the addition of two equivalents of TEA, indicating that **1a**•(TFA)_2_ and **1a**•(TPFB)_2_ can be fully converted back to **1a** (Supplementary Fig. 16), agreeing well with the observed absorption titration results. Therefore, the delocalization of spin density of **1a** could also be reversibly regulated by the reversible acid-base reaction, during which both **1a** and its acid-base adducts remained inherent stability.

The observed enhanced EPR signal intensity was a clue that the diradical character of **1a** might undergo significant changes before and after acid stimulation. Interestingly, the NMR line broadening effect of **1a** became more pronounced when acid was added, indicating an increased amount of paramagnetic species after acid stimulation (Supplementary Figs. 17 and 18). Specifically, taking **1a**•(TPFB)_2_ as an example, the VT NMR spectroscopy experiments indicated that **1a**•(TPFB)_2_ displayed broad signals even at a low temperature (Supplementary Fig. 17), in contrast to the relatively sharp peaks of **1a**. To understand in more detail the differences in the change in magnetic properties, SQUID measurement of a powdered sample of **1a**•(TPFB)_2_ at a temperature gradient ranging from 2 to 330 K was obtained and compared with that of **1a** (Fig. 8c and Supplementary Table 5). As expected, the magnetic susceptibility (*χ*_p_T) of **1a**•(TPFB)_2_ increased to 0.25 emu Kmol^−^^1^ at 300 K, which was significantly larger than that of **1a**. This larger magnetic susceptibility might account for the enhanced EPR signal intensity and more obvious NMR line broadening of **1a**•(TPFB)_2_. The singlet-triplet energy gap (∆*E*_S-T_) of **1a**•(TPFB)_2_ was then estimated to be −1.50 kcal mol^−^^1^ by fitting the data using the Bleaney-Bowers equation, which was smaller than that of **1a**. In addition, DFT calculations predicted that the acid-base adducts of **1a**•(TFA)_2_ and **1a**•(TPFB)_2_ both showed an open-shell singlet diradical ground state with ∆*E*_S-T_ values of −1.98 and −0.78 kcal mol^−^^1^, respectively, which were smaller than that of **1a** (Supplementary Table 6, Supplementary Data 1). The diradical character of **1a**•(TFA)_2_ and **1a**•(TPFB)_2_ were calculated to be 0.64 and 0.78, respectively, which was larger than that of **1a** (Supplementary Table 7, Supplementary Data 1). Therefore, the different values of ∆*E*_S-T_ and *y*_0_ perfectly illustrated the fact that the population of thermally excited triplet species was even more favorable after **1a** formed acid-base adducts in the presence of acid stimulation, thus directly affecting their magnetic properties.

### Light-controlled reversible modulation of UV-vis-NIR absorption and diradical character

**Fig. 9:**
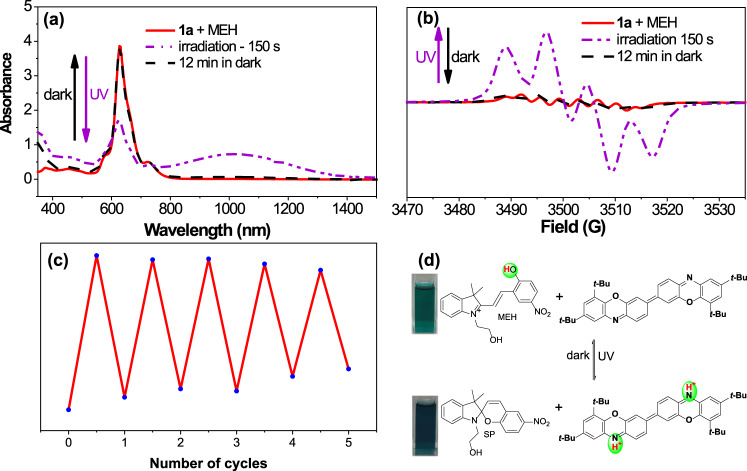
Light-controlled reversible modulation of UV-vis-NIR absorption and diradical character. UV-vis-NIR absorption (**a**) and EPR spectra (**b**) of a solution of **1a** and MEH (mole ratio **1a**: MEH = 1: 5) before irradiation (red line), after irradiation for 150 s (purple dotted line), and kept in the dark for 12 min after irradiation (black dotted line). **c** The reversible changes of the absorbance under cycles of irradiation and darkness. **d** Schematic illustration of the light-controlled reversible protonation and deprotonation process of **1a** through the use of a photoacid (MEH).

The success of the Lewis acid-base adduct approach in enhancing the diradical character of **1a** further inspired us to investigate its photoresponsive properties by using a photoacid as an acid stimulus. Photoacids are often referred to as molecules that can release proton(s) upon light irradiation; they can regain a proton(s) in the dark or by heating, making this process reversible^78,79^. Therefore, the light-controlled reversible acid-base reaction between **1a** and a suitable photoacid via intermolecular proton transfer is theoretically feasible, although **1a** itself is non-photoresponsive^80,81^. A commercially available photoacid merocyanine (MEH) was selected for use in this study. When the mixed solution of **1a** and MEH (mole ratio **1a**: MEH = 1: 5) was illuminated with ultraviolet light at a wavelength of 365 nm, a continuous change in the UV-vis-NIR spectra was observed, and the trend together with the absorption characteristics resembled the experimental acid-titration results (Fig. 9a). After 150 s of irradiation, the new broad long-wavelength absorption from 700–1400 nm reached a maximum, and the color of the solution changed from green to dark blue. Interestingly, the absorption profile and the color returned to their original form when the irradiated mixture was kept in the dark for 12 min (Fig. 9a). Moreover, this photoswitching process displayed good stability over five cycles (Fig. 9c). Similarly, EPR spectroscopy was applied to monitor the photoswitching process in situ, and the distinct octet and quartet hyperfine splitting patterns could be reversibly switched many times without decomposition by alternating between UV irradiation and dark storage (Fig. 9b and Supplementary Fig. 21). The changes in intensity along with the corresponding changes in the hyperfine splitting pattern in the EPR spectra were all consistent with the titration experiment. The proposed working principle of the operation of this responsive system was that the photoacid MEH could transfer to a highly metastable form SP under irradiation and at the same time released a proton that then protonated the nitrogen atom of **1a**; in the dark, the metastable form SP could recapture the proton, regenerating both the photoacid MEH and **1a** (Fig. 9d). Therefore, the UV-vis-NIR absorption and diradical character of **1a** could be well manipulated during this photoswitching process. To the best of our knowledge, this work is the first to realize the light-controlled reversible modulation of the photophysical and magnetic properties of an organic diradicaloid.

## Discussion

In summary, an open-shell nitrogen-centered diradicaloid **1a** was successfully synthesized through a simple and high-yielding two-step synthesis protocol without the need for column chromatography. The ground-state electronic structure of **1a** was systematically investigated by X-ray crystallographic analysis, VT NMR, EPR, and SQUID techniques along with DFT calculations, and all the data collectively suggested that **1a** displayed an open-shell singlet ground state with a small singlet-triplet energy gap and a modest diradical character. Interestingly, the photophysical and magnetic properties of **1a** changed dramatically in the presence of acidic stimulus, accompanied by a decrease in the singlet-triplet energy gap and an increase in diradical character. Detailed theoretical and experimental studies disclosed that the fundamental cause of the change in these properties was that the formation of the Lewis acid-base adduct prominently impacted the ground-state electronic structures of **1a** as well as its diradical character and spin density distributions. Therefore, this work for the first time demonstrates a rare and interesting stimuli-responsive organic diradical system to the best of our knowledge that is capable of reversibly altering its photophysical properties and diradical character upon exposure to external stimuli. It is expected that our studies would not only provide a simple synthetic strategy to design open-shell diradicaloid but also shed some light on the development of the advanced organic diradicaloids and related materials with tunable stimuli-responsive electronic properties and diradical character.

## Methods

All reagents and starting materials were obtained from commercial suppliers and used without further purification. All air-sensitive reactions were carried out under inert N_2_ atmosphere. The ^1^H NMR, ^11^B NMR, ^13^C NMR and 1D NOESY spectra were recorded in solution of CD_2_Cl_2_ and DMSO-*d*_6_ on Bruker 400 MHz, 500 MHz spectrometer. 2D NMR (NOESY) and variable temperature NMR were measured in solution of CD_2_Cl_2_ on Agilent DD2 600 MHz. UV-vis-NIR spectra were recorded in a quartz cell (light path 10 mm) on a Shimadzu UV2700 UV-visible spectrophotometer. Fluorescence spectra and photoluminescence quantum yields (Ф) were recorded on HORIBA Duetta. Cyclic voltammetry was recorded on a Bio-Logic SAS SP-150 spectrometer in anhydrous DCM containing *n*-Bu_4_NPF_6_ (0.1 M) as supporting electrolyte at a scan rate of 20 mV/s at room temperature. The CV cell has a glassy carbon electrode, a Pt wire counter electrode, and an Ag/Ag^+^ reference electrode. The potential was externally calibrated against the ferrocene/ferrocenium (Fc/Fc^+^) couple. The highest occupied molecular orbital (HOMO) and lowest unoccupied molecular orbital (LUMO) energy levels were calculated based on the equations: *E*_HOMO/LUMO_ = −(4.80 + *E*_onset_^ox^/*E*_onset_^red^) eV. EPR spectra for radicals were obtained on Bruker EMX instrument EMXPLUS-10/12. EPR spectra simulation was conducted on the Bruker SpinFit software. For SQUID measurement, magnetic susceptibility of powder sample (30 mg) was measured in a polycarbonate capsule fitted in a plastic straw as a function of temperature in heating (2 K → 330 K) mode with 30 s of temperature stability at each temperature (1 K increment in a range 2–10 K, 2 K increment in a range 10–20 K, 5 K increment in a range 20–100 K, 10 K increment in a range 100–330 K,) at 1.0 T using a SQUID magnetometer (Quantum MPMS3). The data was corrected for both sample diamagnetism (Pascal’s constants) and the diamagnetism of the sample holder (polycarbonate capsule). The single crystals of this work were measured on Bruker Apex duo equipment with Cu Kα radiation (λ = 1.54184 Å). The HR-ESI mass spectra were performed on Q Exactive Focus (Thermo Scientific, USA). The singlet-triplet energy gap (∆*E*_S-T_ or 2 *J*/*k*_B_) was determined by fitting the susceptibility data using the Bleaney-Bowers equation,

$${\chi }_{{{{{{\bf{M}}}}}}}=\frac{{{{{{\bf{2}}}}}}{{{{{\boldsymbol{N}}}}}}{{{{{{\boldsymbol{g}}}}}}}^{{{{{{\bf{2}}}}}}}{{{{{{\boldsymbol{\beta }}}}}}}^{{{{{{\bf{2}}}}}}}}{{{{{{{\boldsymbol{k}}}}}}}_{{{{{{\bf{B}}}}}}}{{{{{\boldsymbol{T}}}}}}}\frac{{{{{{\bf{1}}}}}}}{{{{{{\bf{3}}}}}}+{{{{{\mathbf{exp }}}}}}\left(-\frac{{{{{{\bf{2}}}}}}{{{{{\boldsymbol{J}}}}}}}{{{{{{{\boldsymbol{k}}}}}}}_{{{{{{\bf{B}}}}}}}{{{{{\boldsymbol{T}}}}}}}\right)}\left({{{{{\bf{1}}}}}}-{{{{{\boldsymbol{\rho }}}}}}\right)+\frac{{{{{{\boldsymbol{N}}}}}}{{{{{{\boldsymbol{g}}}}}}}^{{{{{{\bf{2}}}}}}}{{{{{{\boldsymbol{\beta }}}}}}}^{{{{{{\bf{2}}}}}}}}{{{{{{\bf{2}}}}}}{{{{{{\boldsymbol{k}}}}}}}_{{{{{{\bf{B}}}}}}}{{{{{\boldsymbol{T}}}}}}}{{{{{\boldsymbol{\rho }}}}}}$$

where −2*J* is correlated to the excitation energy from the ground state to the first excited state, *ρ* is the content of paramagnetic impurities, *T* is the temperature, *k*_B_ is Boltzmann constant, *N* is Avogadro constant.

## Supplementary information

Shi_PR File

Supplementary Information

Description of Additional Supplementary Files

Supplementary Data 1

Supplementary Data 2

Supplementary Data 3

Supplementary Data 4

Supplementary Data 5

Supplementary Data 6

Supplementary Data 7

## Data Availability

The data that support the findings of this study are available from the authors on reasonable request, see author contributions for specific data sets. Experimental details, additional characterizations, computational details, and figures including synthetic route, UV−vis-NIR spectra, ^1^H and ^13^C NMR spectra, 1D and 2D NOESY NMR spectra, MS spectra, EPR spectra, spin density and spin population (PDF) are described in the Supplementary Information. Cartesian coordinates are deposited in Supplementary Data 1 file. The X-ray crystallographic coordinates for structures reported in this study have been deposited at the Cambridge Crystallographic Data Centre (CCDC), under deposition numbers CCDC 2132537 (**4a**), 2132539 (**4b**), 2132540 (**4d**), 2104545 (**1a**), 2104547 (**1b**), and 2132542 (**1a**^**2+**^). These data can be obtained free of charge from The Cambridge Crystallographic Data Centre via www.ccdc.cam.ac.uk/data_request/cif. The CIF files of CCDC 2132537 (**4a**), 2132539 (**4b**), 2132540 (**4d**), 2104545 (**1a**), 2104547 (**1b**), and 2132542 (**1a**^**2+**^) are also included as Supplementary Data 2-Data 7.
