# Supplementary material for: Design of an open-shell nitrogen-centered diradicaloid with tunable stimuli-responsive electronic properties
Source: Commun Chem. 2022 Oct 14;5:127. doi: 10.1038/s42004-022-00747-8 (PMC9814612; doi:10.1038/s42004-022-00747-8)
Supplement: Supplementary file 2 — Supplementary Information [file 42004_2022_747_MOESM2_ESM.pdf]

Supporting Information

Design of an open-shell nitrogen-centered diradicaloid with tunable stimuli-responsive electronic properties

Bin Huang, Hao Kang, Chang-Wei Zhang, Xiao-Li Zhao, Xueliang Shi,* Hai-Bo Yang

Shanghai Key Laboratory of Green Chemistry and Chemical Processes, School of Chemistry and Molecular Engineering, East China Normal University, 3663 N. Zhongshan Road, Shanghai 200062, People’s Republic of China

Corresponding authors: xlshi@chem.ecnu.edu.cn

Table of Contents

1. Supplementary methods 1
2. Synthesis and characterization 2
3. X-ray crystallographic analysis 8
4. Lewis acid-base stimuli-responsive properties 16
5. Theoretical calculation 23
    5.1 Structure simulation 24
    5.2 Conformational isomer interconversion pathway 24
    5.3 Nucleus independent chemical shift (NICS) values 25
    5.4 Spin density and spin population 26
    5.5 Molecular thermochemistry properties 29
    5.6 Diradical character 30
    5.7 Theoretical UV-Vis spectrum 30
6. ^{1}H NMR and ^{13}C NMR spectra of new compounds 31
7. Mass spectra of new compounds 36
8. Supplementary references 39

1. Supplementary methods

All reagents and starting materials were obtained from commercial suppliers and used without further purification. All air-sensitive reactions were carried out under inert N_2 atmosphere. The ^{1}H NMR, ^{11}B NMR, ^{13}C NMR spectra were recorded in solution of CD_2Cl_2 and DMSO-d_6 on Bruker 400 MHz, 500 MHz spectrometer. 2D NMR (NOESY) and variable temperature NMR were measured in solution of CD_2Cl_2 on Agilent DD2 600 MHz. Coupling constants (J) are denoted in Hz and chemical shifts (δ) are denoted in ppm. Multiplicities are denoted as follows: s = singlet, d = doublet, b = broaden and m = multiplet. UV-vis-NIR spectra were recorded in a quartz cell (light path 10 mm) on a Shimadzu UV2700 UV-visible spectrophotometer. Fluorescence spectra and photoluminescence quantum yields (Φ) were recorded on HORIBA Duetta. Cyclic voltammetry was recorded on a Bio-Logic SAS SP-150 spectrometer in anhydrous DCM containing n-Bu_4NPF_6 (0.1 M) as supporting electrolyte at a scan rate of 20 mV/s at room temperature. The CV cell has a glassy carbon electrode, a Pt wire counter electrode, and an Ag/Ag^+ reference electrode. The potential was externally calibrated against the ferrocene/ferrocenium (Fc/Fc^+) couple. The highest occupied molecular orbital (HOMO) and lowest unoccupied molecular orbital (LUMO) energy levels were calculated based on the equations: $E_{HOMO/LUMO} = -(4.80 + E_{onset}^{ox}/E_{onset}^{red})$ eV. EPR spectra for radicals were obtained on Bruker EMX instrument EMXPLUS-10/12. EPR spectra simulation was conducted on the Bruker SpinFit software. For SQUID measurement, magnetic susceptibility of powder sample (30 mg) was measured in a polycarbonate capsule fitted in a plastic straw as a function of temperature in heating (2 K→ 330 K) mode with 30 seconds of temperature stability at each temperature (1 K increment in a range 2-10 K, 2 K increment in a range 10-20 K,5 K increment in a range 20-100 K, 10 K increment in a range 100-330 K,) at 1.0 T using a SQUID magnetometer (Quantum MPMS3). The data was corrected for both sample diamagnetism (Pascal's constants) and the diamagnetism of the sample holder (polycarbonate capsule).[S1] The single crystals of this work were measured on Bruker Apex duo equipment with Cu Kα radiation (λ = 1.54184 Å). The HR-ESI mass spectra were performed on Q Exactive Focus (Thermo Scientific, USA). The singlet-triplet energy gap ($\Delta E_{S\text{-}T}$ or $2J/k_B$) was determined by fitting the susceptibility data using the Bleaney-Bowers equation,

$$\chi_{\mathbf{M}} = \frac{2Ng^2\beta^2}{k_B T}\frac{1}{3+\exp\left(-\frac{2J}{k_B T}\right)}(1-\rho) + \frac{Ng^2\beta^2}{2k_B T}\rho$$

where -2J is correlated to the excitation energy from the ground state to the first excited state, ρ is the content of paramagnetic impurities, T is the temperature, k_B is Boltzmann constant, N is Avogadro constant.

2. Synthesis and characterization

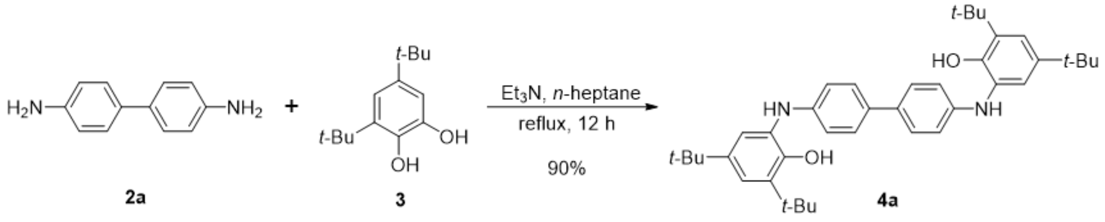

Compound 4a

To a *n*-heptane solution (30 mL) of compound **3** (2.22 g, 10 mmol), compound **2a** (0.92 g, 5.0 mmol) and triethylamine (0.05 g, 0.5 mmol) were added slowly. Then the mixture was heated to reflux for 12 h. After cooling to the room temperature, the mixture was kept at 0 °C for 1.0 h. The precipitate was collected by vacuum filtration and washed by cold *n*-heptane for three times. The residue was dried in vacuo to give compound **4a** (2.67 g, 4.5 mmol) as a grey powder in 90% yield. ^{1}H NMR (400 MHz, DMSO-d_6, 298 K) δ (ppm) 7.95 (s, 2H), 7.38 (d, *J* = 8.5 Hz, 4H), 7.17 (s, 2H), 7.05 (d, *J* = 2.4 Hz, 2H), 6.98 (d, *J* = 2.4 Hz, 2H), 6.78 (d, *J* = 8.5 Hz, 4H), 1.40 (s, 18H), 1.24 (s, 18H). ^{13}C NMR (100 MHz, DMSO-d_6, 298 K) δ (ppm) 147.42, 145.31, 141.23, 136.59, 130.67, 130.56, 126.59, 118.76, 118.64, 115.57, 35.17, 34.41, 31.93, 30.12. HR-ESI-MS (*m/z*): $[M+H]^+$ calculated for $C_{40}H_{53}N_2O_2$ 593.4062, found 593.4097.

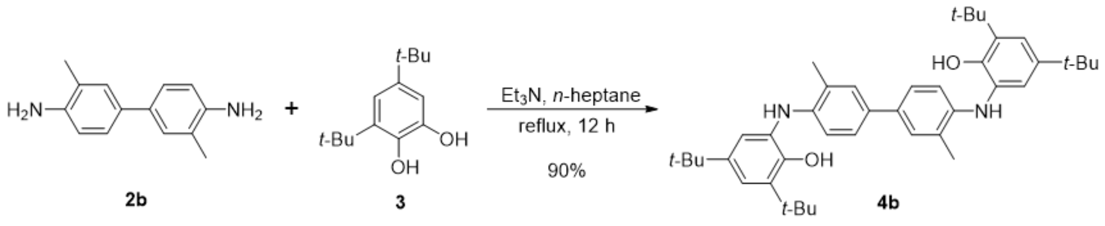

Compound 4b

To a *n*-heptane solution (30 mL) of compound **3** (2.22 g, 10 mmol), compound **2b** (1.06 g, 5.0 mmol) and triethylamine (0.05 g, 0.5 mmol) were added slowly. Then the mixture was heated to reflux for 12 h. After cooling to the room temperature, the mixture was kept at 0 °C for 1.0 h. The precipitate was collected by vacuum filtration and was washed by cold *n*-heptane for three times. The residue was dried in vacuo to give compound **4b** (2.79 g, 4.5 mmol) as a grey powder in 90% yield. ^{1}H NMR (400 MHz, DMSO-d_6, 298 K) δ (ppm) 7.97 (s, 2H), 7.35 (d, *J* = 2.2 Hz, 2H), 7.21 (dd, *J* = 8.4, 2.3 Hz, 2H), 6.98 (d, *J* = 2.4 Hz, 2H), 6.94 (d, *J* = 2.3 Hz, 2H), 6.55 (d, *J* = 8.4 Hz, 2H), 6.38 (s, 2H), 2.29 (s, 6H), 1.40 (s, 18H), 1.23 (s, 18H). ^{13}C NMR (100 MHz, DMSO-d_6, 298 K) δ (ppm) 147.24, 143.36, 141.27, 136.56, 131.14, 130.80, 128.19, 125.62, 124.06, 119.37, 118.48, 114.48, 35.18, 34.39, 31.92, 30.15, 18.50. HR-ESI-MS (*m/z*): $[M+H]^+$ calculated for $C_{42}H_{57}N_2O_2$ 621.4375, found 621.4397.

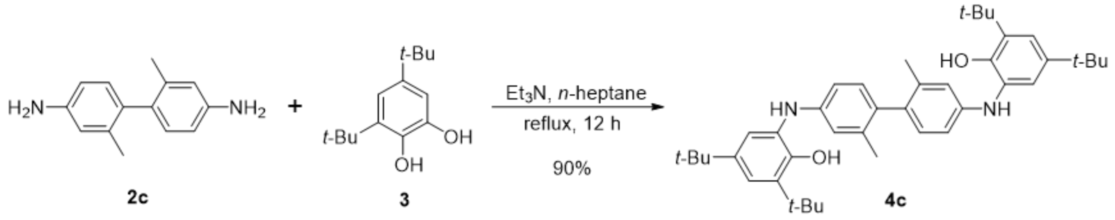

Compound 4c

To a *n*-heptane solution (30 mL) of compound **3** (2.22 g, 10 mmol), compound **2c** (1.06 g, 5.0 mmol) and triethylamine (0.05 g, 0.5 mmol) were added slowly. Then the mixture was heated to reflux for 12 h. After cooling to the room temperature, the mixture was kept at 0 °C for 1.0 h. The precipitate was collected by vacuum filtration and was washed by cold *n*-heptane for three times. The residue was dried in vacuo to give compound **4c** (2.79 g, 4.5 mmol) as a reddish-brown powder in 90% yield. ^{1}H NMR (500 MHz, DMSO-d_6, 298 K) δ (ppm) 7.94 (s, 2H), 7.10-7.06 (m, 4H), 6.98 (d, J = 2.4 Hz, 2H), 6.80 (d, J = 8.2 Hz, 2H), 6.68 (d, J = 2.4 Hz, 2H), 6.57 (dd, J = 8.3, 2.4 Hz, 2H), 1.93 (s, 6H), 1.40 (s, 18H), 1.24 (s, 18H). ^{13}C NMR (125 MHz, DMSO-d_6, 298 K) δ (ppm) 147.50, 145.47, 141.06, 136.43, 136.38, 131.62, 130.69, 130.51, 119.13, 118.57, 116.13, 112.34, 35.16, 34.41, 31.94, 30.10, 20.52. HR-ESI-MS (*m/z*): $[M+H]^+$ calculated for $C_{42}H_{57}N_2O_2$ 621.4375, found 621.4402.

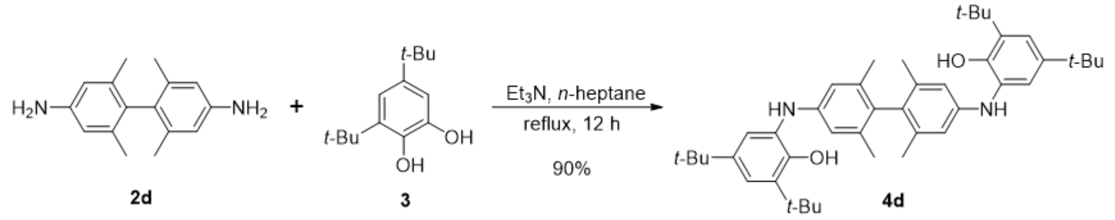

Compound 4d

To a *n*-heptane solution (30 mL) of compound **3** (2.22 g, 10 mmol), compound **2d** (1.2 g, 5.0 mmol) and triethylamine (0.05 g, 0.5 mmol) were added slowly. Then the mixture was heated to reflux for 12 h. After cooling to the room temperature, the mixture was kept at 0 °C for 1.0 h. The precipitate was collected by vacuum filtration and was washed by cold *n*-heptane for three times. The residue was dried in vacuo to give compound **4d** (2.92 g, 4.5 mmol) as a reddish-brown powder in 90% yield. ^{1}H NMR (500 MHz, DMSO-d_6, 298 K) δ (ppm) 7.91 (s, 2H), 7.09 (d, J = 2.3 Hz, 2H), 6.99-6.93 (m, 4H), 6.57 (s, 4H), 1.77 (s, 12H), 1.40 (d, J = 2.3 Hz, 18H), 1.24 (s, 18H). ^{13}C NMR (125 MHz, DMSO-d_6, 298 K) δ (ppm) 147.05, 144.79, 140.95, 136.47, 136.35, 130.85, 130.15, 118.77, 118.11, 114.23, 35.16, 34.41, 31.92, 30.11, 26.82, 20.56. ppm. HR-ESI-MS (*m/z*): $[M+H]^+$ calculated for $C_{44}H_{61}N_2O_2$ 649.4688, found 649.4717.

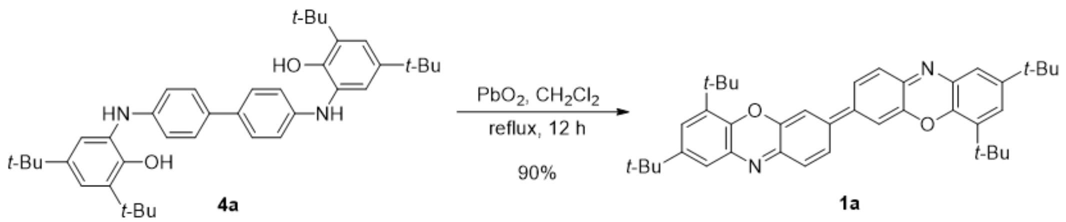

Compound 1a

Compound **4a** (592 mg, 1.0 mmol) was dissolved in DCM (20 mL), then lead dioxide (480 mg, 2.0 mmol) was added into the solution. The mixture was heated to reflux for 12 h. After the mixture cooled to the room temperature, precipitate in solution was filtered out, and the solution was collected. The solvent was removed under reduced pressure, and the residue was washed by methanol for several times to give compound **1a** (528 mg, 0.9 mmol) as a black purple solid in 90% yield. The target compound was further purified by recrystallization from MeOH/CH_2Cl_2 for magnetic properties test. ^{1}H NMR (600 MHz, CD_2Cl_2, 233 K) δ (ppm) 7.68 (d, J = 9.5 Hz, 2H), 7.25 (s, 2H), 7.21 (s, 2H), 7.13 (s, 2H), 7.02 (b, 2H), 1.46 (s, 18H), 1.28 (s, 18H). HR-ESI-MS (*m/z*): $[M+H]^+$ calculated for $C_{40}H_{47}N_2O_2$ 587.3559, found 587.3623.

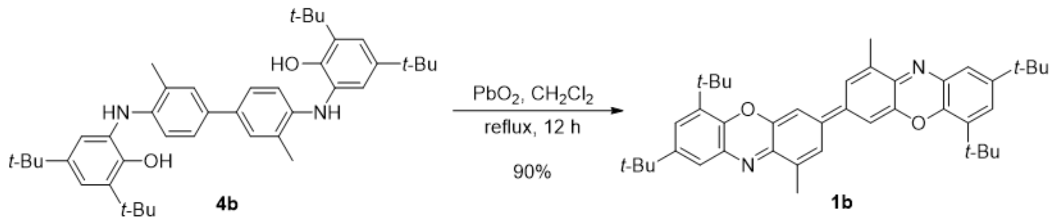

Compound 1b

Compound **4b** (620 mg, 1.0 mmol) was dissolved in DCM (20 mL), then lead dioxide (480 mg, 2.0 mmol) was added into the solution. The mixture was heated to reflux for 12 h. After the mixture cooled to the room temperature, precipitate in solution was filtered out, and the solution was collected. The solvent was removed under reduced pressure, and the residue was washed by methanol for several times to give compound **1b** (528 mg, 0.9 mmol) as a black purple solid in 90% yield. ^{1}H NMR (600 MHz, CD_2Cl_2, 248 K) δ (ppm) 7.53 (s, 2H), 7.25 (s, 2H), 7.20 (s, 2H), 7.00 (s, 2H), 2.34 (s, 6H), 1.45 (s, 18H), 1.30 (s, 18H). HR-ESI-MS (*m/z*): $[M+H]^+$ calculated for $C_{42}H_{51}N_2O_2$ 615.3872, found 615.3931.

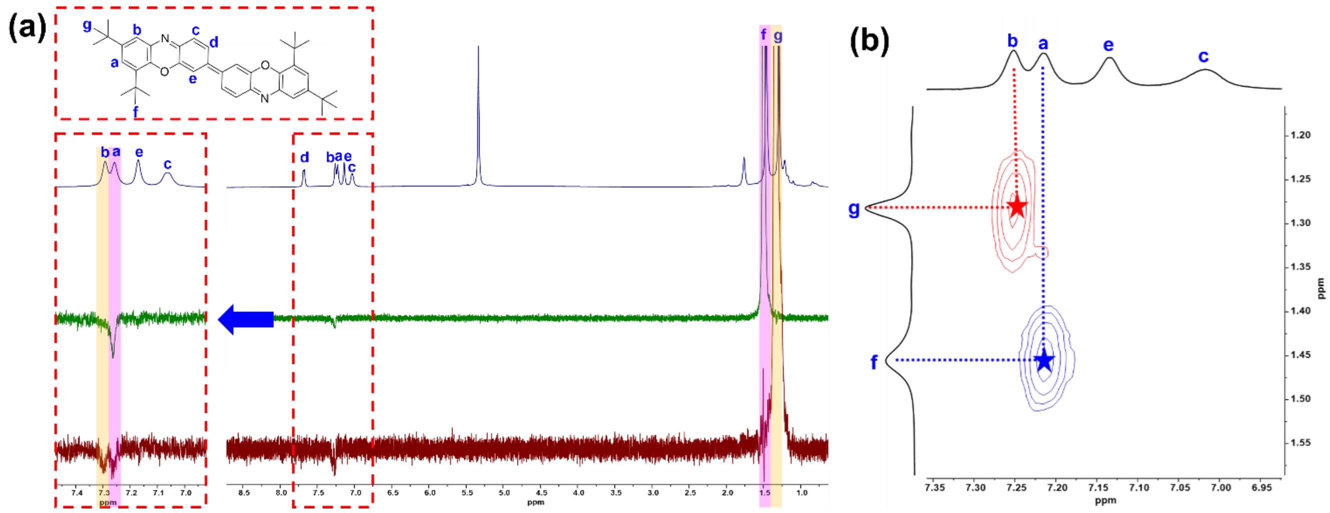

Supplementary Fig. 1: (a) 1D NOESY NMR (500 MHz, CD_2Cl_2, 233 K) spectrum and (b) 2D NOESY NMR (600 MHz, CD_2Cl_2, 233 K) spectrum of **1a**.

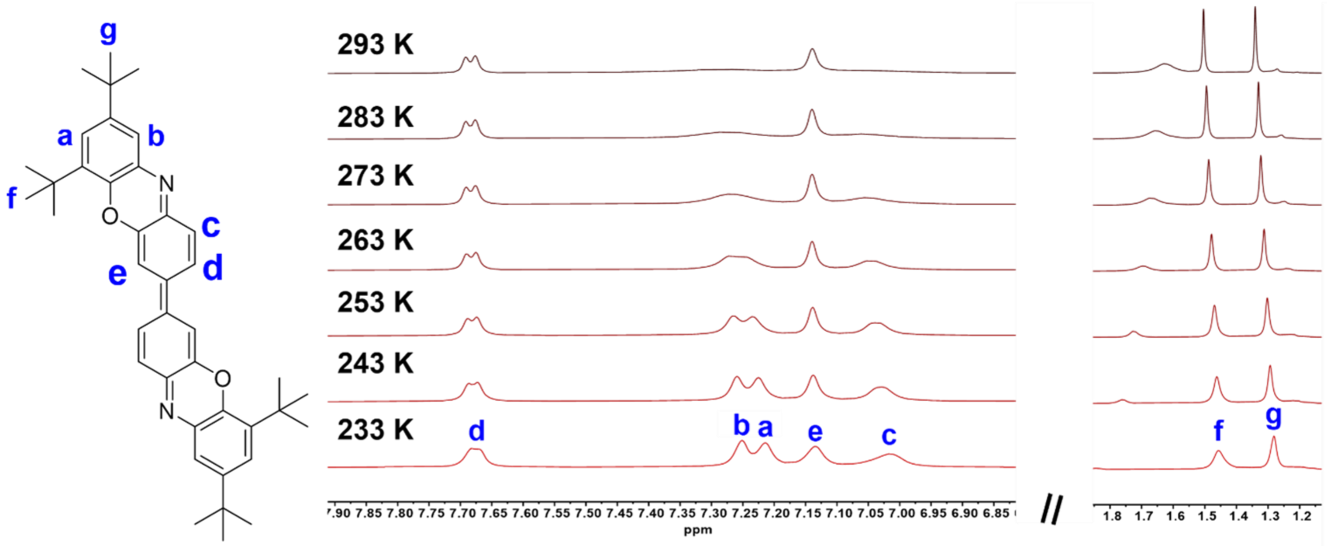

Supplementary Fig. 2: Variable-temperature ^{1}H NMR (600 MHz, CD_2Cl_2) spectra of **1a** from 233 K to 293 K.

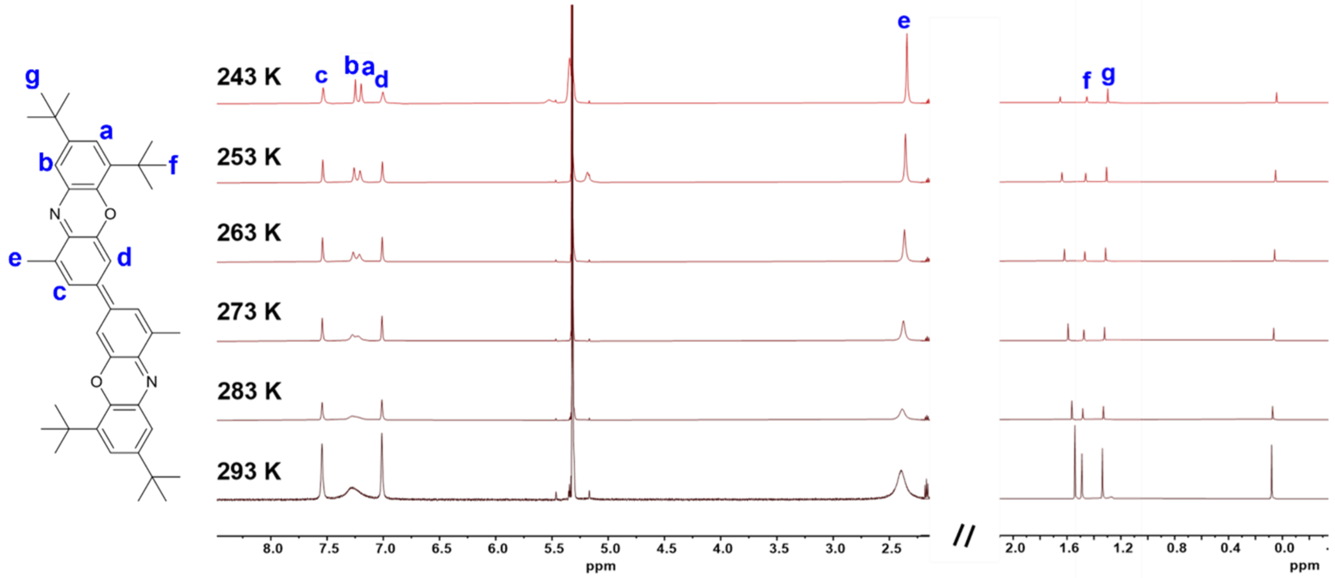

Supplementary Fig. 3: Variable-temperature ^{1}H NMR (600 MHz, CD_2Cl_2) spectra of **1b** from 243 K to 293 K.

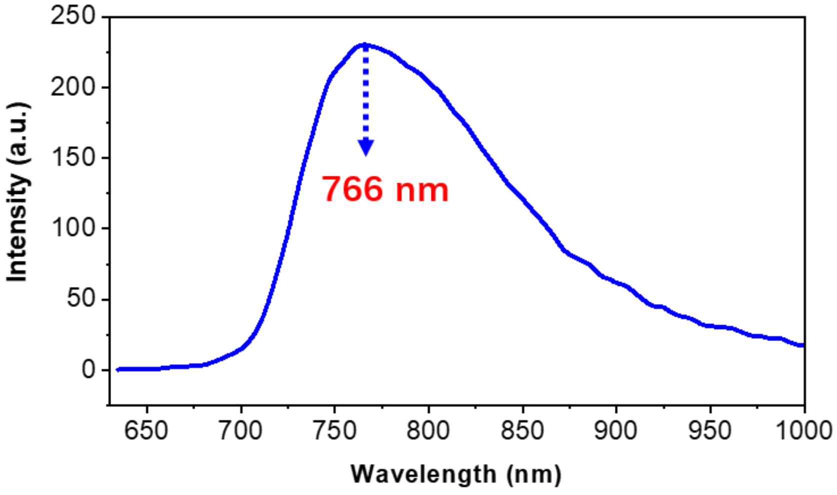

Supplementary Fig. 4: Emission spectrum of **1a** (14 μM) in toluene (λ_{ex} = 620 nm). The photoluminescence quantum yields (Φ) of **1a** in toluene was determined as ~ 4%.

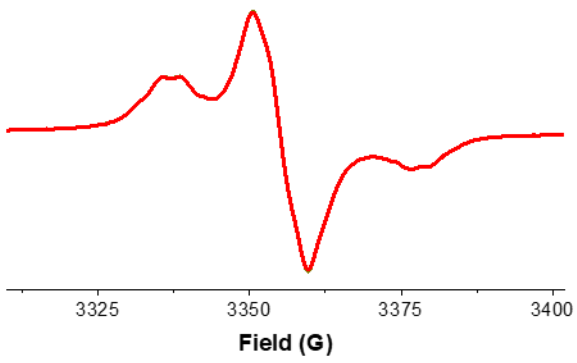

Supplementary Fig. 5: Solid-state EPR of **1a** at 298 K.

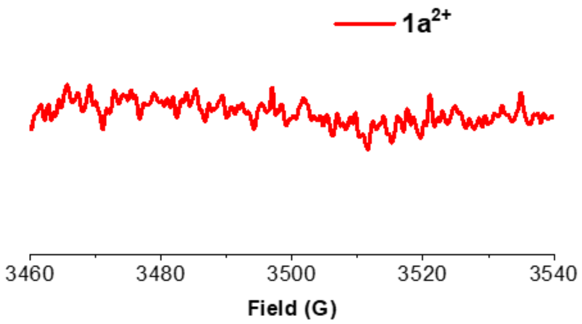

Supplementary Fig. 6: EPR spectrum of $\mathbf{1a^{2+}}$ in CH_2Cl_2 at 298 K.

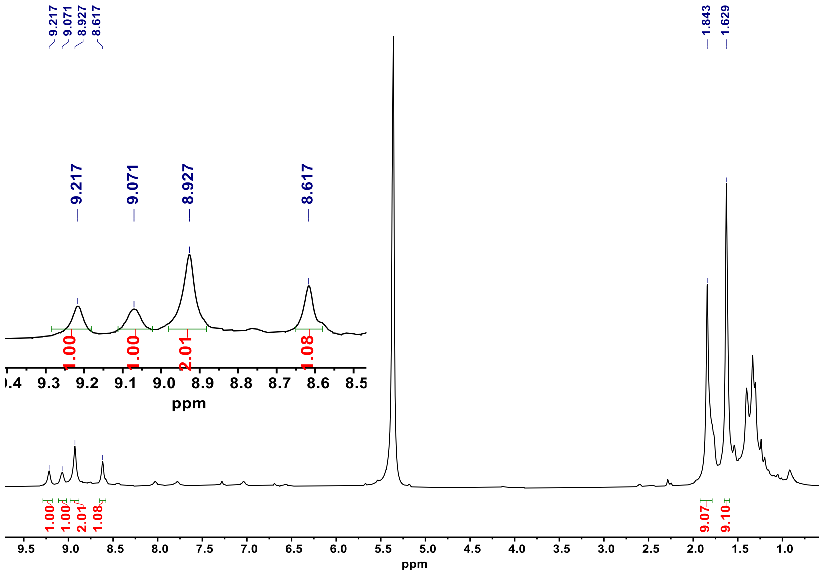

Supplementary Fig. 7: ^{1}H NMR (500 MHz, CD_2Cl_2, 298 K) spectrum of $\mathbf{1a^{2+}}$.

3. X-ray crystallographic analysis

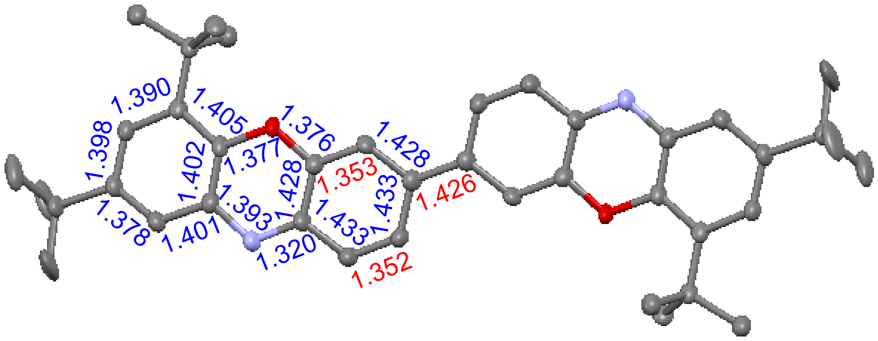

Supplementary Fig. 8: Single crystal structure of **1a** and bond-length analysis based on X-ray diffraction data. Hydrogen atoms are omitted for clarity.

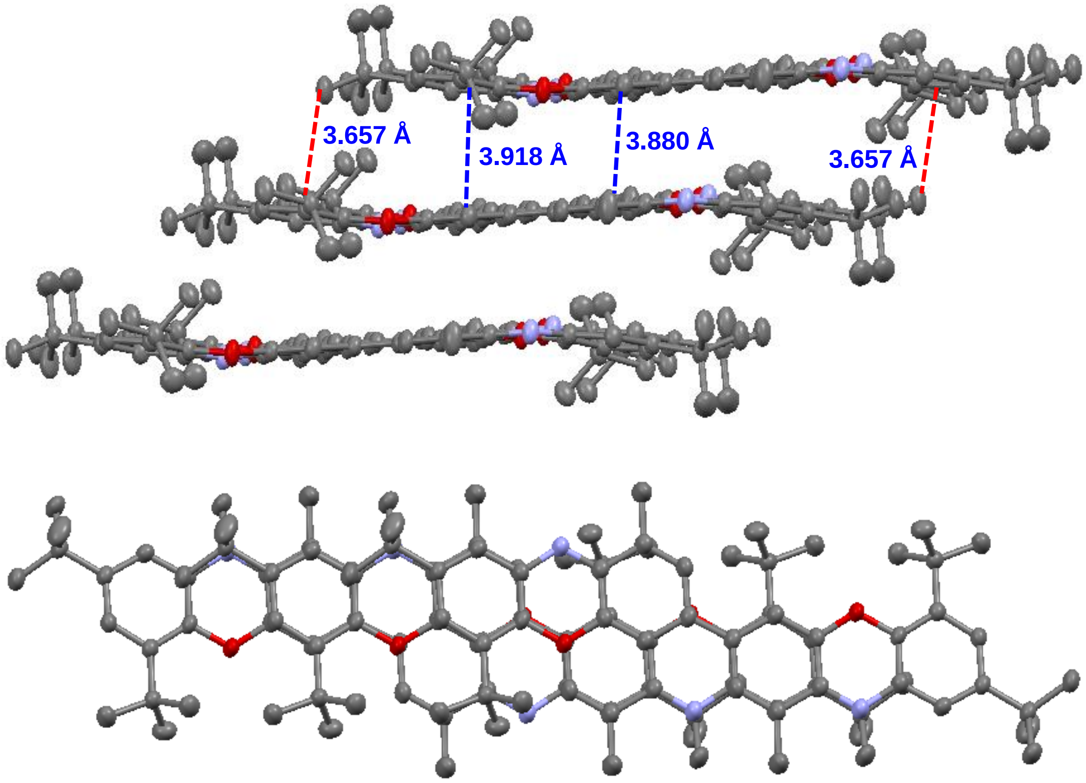

Supplementary Fig. 9: Packing structure of **1b** at 170 K in the crystalline state. Other hydrogen atoms are omitted for clarity.

Supplementary Table 1: Bond-length (Å) data of **1a**, **1b**.

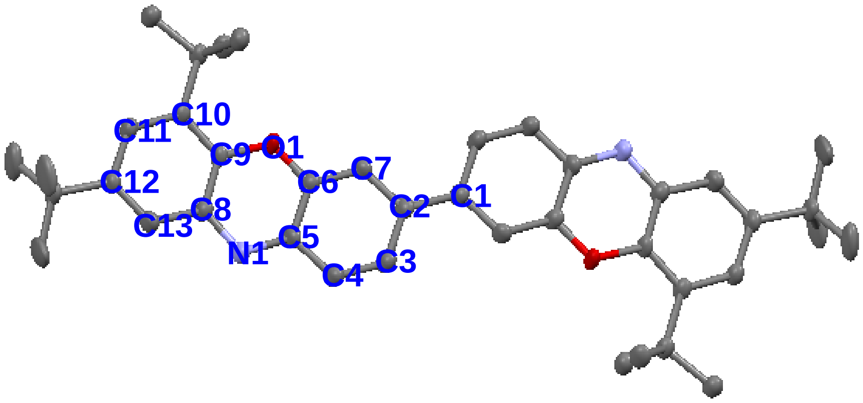

	C1-C2	C2-C3	C3-C4	C4-C5	C5-C6	C6-C7	C7-C2	C5-N1	N1-C8
**1a** (Å)	1.426	1.433	1.352	1.433	1.428	1.353	1.428	1.320	1.393
**1b** (Å)	1.426	1.442	1.356	1.444	1.434	1.354	1.423	1.323	1.387

	C8-C9	C9-O1	O1-C6	C9-C10	C10-C11	C11-C12	C12-C13	C13-C8
**1a** (Å)	1.402	1.377	1.376	1.405	1.390	1.398	1.378	1.401
**1b** (Å)	1.402	1.387	1.381	1.410	1.391	1.394	1.394	1.409

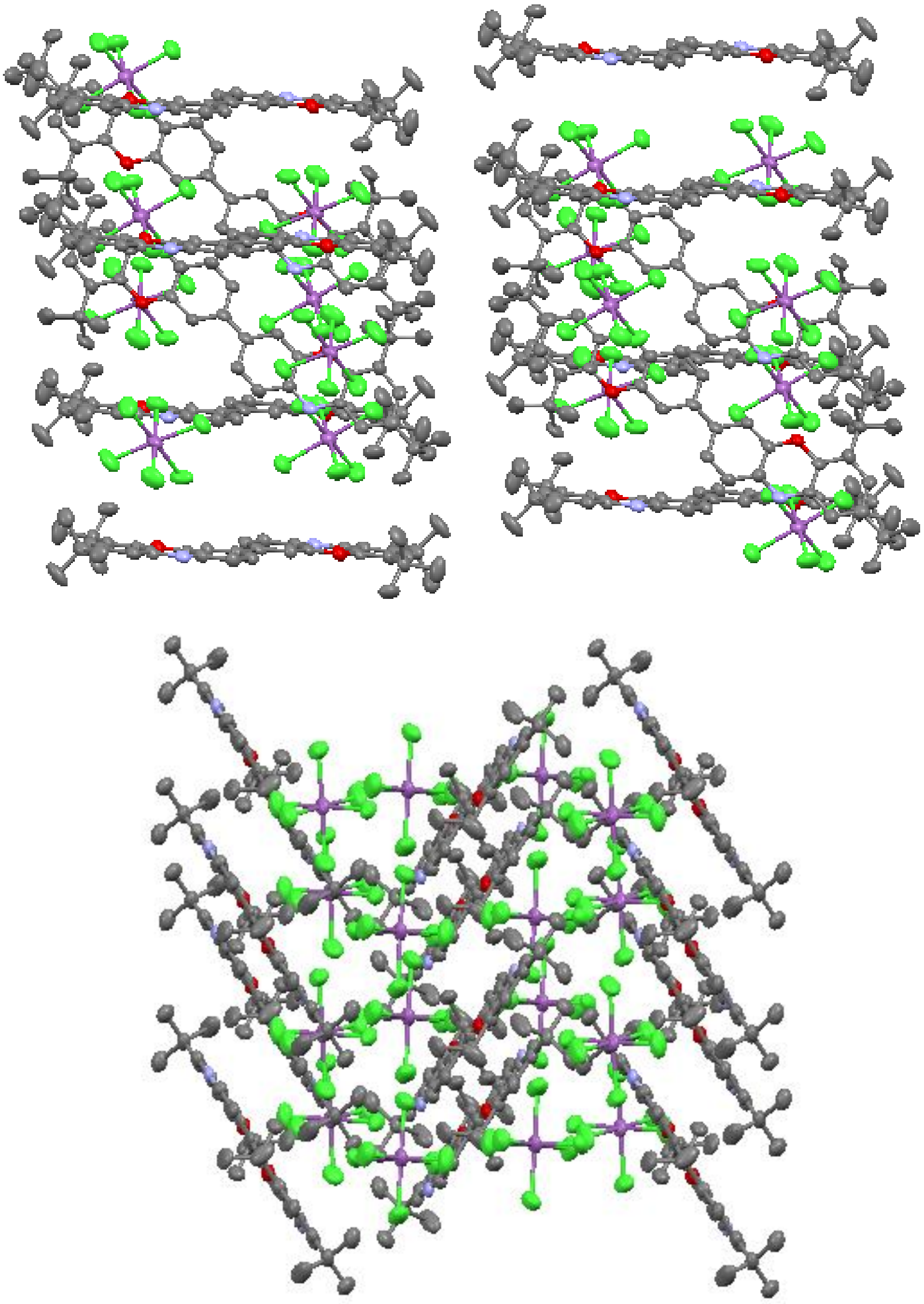

Supplementary Fig. 10: Packing structure of **1a**$^{2+}$ in the crystalline state in the crystalline state. Hydrogen atoms are omitted for clarity.

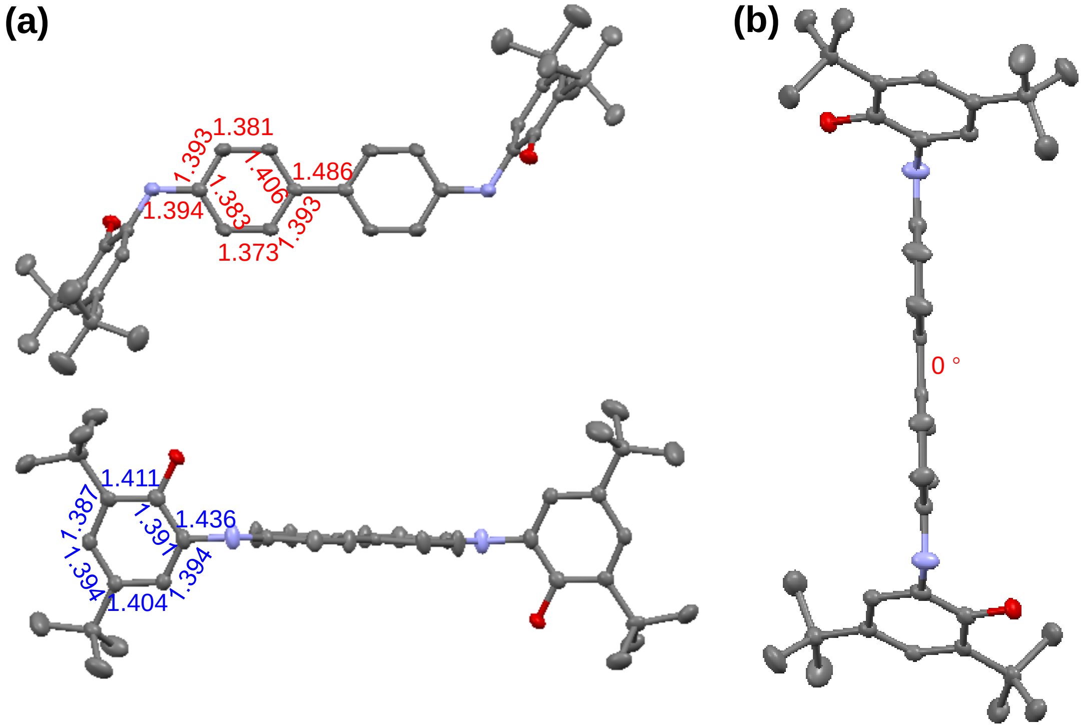

Supplementary Fig. 11: (a) Bond-length analysis of **4a** and (b) torsion angle of two benzene rings in **4a** based on X-ray diffraction data. Hydrogen atoms are omitted for clarity.

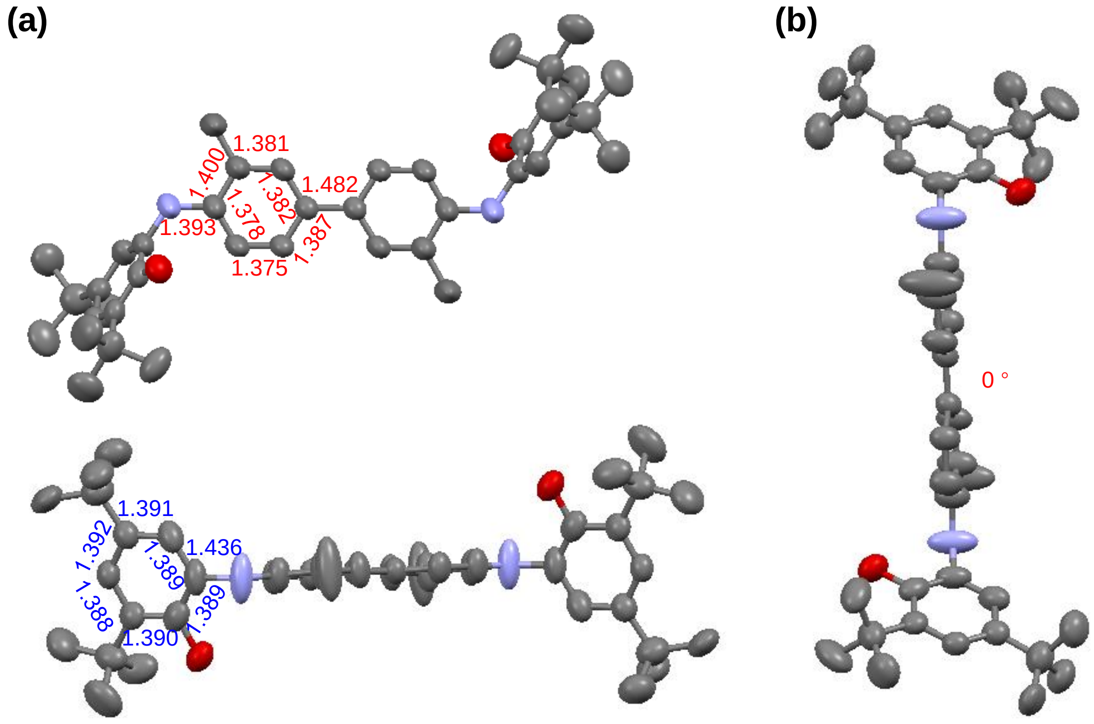

Supplementary Fig. 12: (a) Bond-length analysis of **4b** and (b) torsion angle of two benzene rings in **4b** based on X-ray diffraction data. Hydrogen atoms are omitted for clarity.

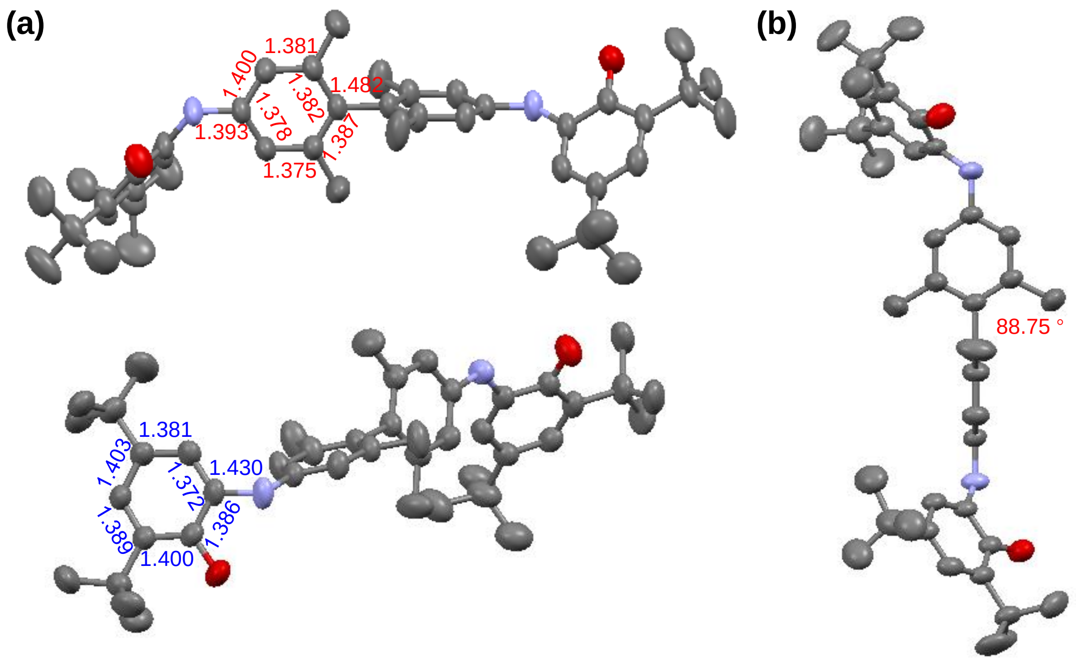

Supplementary Fig. 13: (a) Bond-length analysis of **4d** and (b) torsion angle of two benzene rings in **4d** based on X-ray diffraction data. Hydrogen atoms are omitted for clarity.

Supplementary Table 2: X-ray single crystal data of **4a** and **4b**.

**Compound**	**4a**	**4b**
Empirical formula	$C_{40}H_{50}N_2O_2$	$C_{42}H_{54}N_2O_2$
Formula weight	590.82	618.87
Temperature/K	100.01(10)	293.1(8)
Crystal system	monoclinic	orthorhombic
Space group	$P2_1/c$	Pbca
a / Å	19.1147(4)	9.5096(3)
b / Å	10.0807(2)	12.0422(4)
c / Å	9.0952(2)	34.2936(10)
α / °	90	90
β / °	97.220(2)	90
γ / °	90	90
Volume / $Å^3$	1738.65(6)	3927.2(2)
Z	2	4
ρ_{calc}g / cm^3	1.129	1.047
μ / mm^{-1}	0.527	0.485
F (000)	640.0	1344.0
Crystal size / mm^3	0.42 × 0.26 × 0.18	0.42 × 0.26 × 0.18
Radiation	CuKα (λ = 1.54184)	CuKα (λ = 1.54184)
2θ range for data collection / °	9.938 to 134.156	10.318 to 134.156
Index ranges	-22 ≤ h ≤ 22, -12 ≤ k ≤ 12, -10 ≤ l ≤ 10	-11 ≤ h ≤ 11, -8 ≤ k ≤ 14, -40 ≤ l ≤ 40
Reflections collected	36061	37940
Independent reflections	3032 R_{int} = 0.0709, R_{sigma} = 0.0261	3439 R_{int} = 0.0576, R_{sigma} = 0.0233
Data / restraints / parameters	3032 / 0 / 215	3439/96/284
Goodness-of-fit on F^2	1.116	1.065
Final R indexes [I >= 2σ (I)]	R_1 = 0.1029, wR_2 = 0.2742	R_1 = 0.0986, wR_2 = 0.2733
Final R indexes [all data]	R_1 = 0.1044, wR_2 = 0.2749	R_1 = 0.1141, wR_2 = 0.2865
Largest diff. peak / hole / e $Å^{-3}$	0.65/-0.41	0.34/-0.32
CCDC	2132537	2132539

Supplementary Table 3: X-ray single crystal data of **4d** and **1a**.

**Compound**	**4d**	**1a**
Empirical formula	$C_{44}H_{60}N_2O_2$	$C_{40}H_{46}N_2O_2$
Formula weight	648.94	586.79
Temperature/K	293.36(12)	100.01(10)
Crystal system	triclinic	monoclinic
Space group	P-1	$P2_1/n$
a / Å	10.2479(3)	6.0491(3)
b / Å	12.4899(4)	18.1428(5)
c / Å	21.9716(7)	15.3109(5)
α / °	74.823(3)	90
β / °	79.678(3)	97.068(4)
γ / °	73.455(3)	90
Volume / $Å^3$	2585.11(15)	1667.57(11)
Z	2	2
ρ_{calc}g / cm^3	0.834	1.169
μ / mm^{-1}	0.383	0.549
F (000)	708.0	632.0
Crystal size / mm^3	0.38 × 0.26 × 0.18	0.32 × 0.12 × 0.08
Radiation	CuKα (λ = 1.54184)	CuKα (λ = 1.54184)
2θ range for data collection / °	9.06 to 134.158	7.59 to 134.116
Index ranges	$-12 \le h \le 11$, $-14 \le k \le 14$, $-26 \le l \le 22$	$-5 \le h \le 7$, $-18 \le k \le 21$, $-17 \le l \le 18$
Reflections collected	25519	14335
Independent reflections	8913 R_{int} = 0.0356, R_{sigma} = 0.0379	2965 R_{int} = 0.1332, R_{sigma} = 0.0823
Data / restraints / parameters	8913/102/489	2965/6/205
Goodness-of-fit on F^2	1.058	1.033
Final R indexes [I >= 2σ (I)]	R_1 = 0.0755, wR_2 = 0.2340	R_1 = 0.0758, wR_2 = 0.2092
Final R indexes [all data]	R_1 = 0.0949, wR_2 = 0.2531	R_1 = 0.1024, wR_2 = 0.2387
Largest diff. peak / hole / e $Å^{-3}$	0.47/-0.41	0.35/-0.45
CCDC	2132540	2104545

Supplementary Table 4: X-ray single crystal data of **1b and 1a^{2+}**.

**Compound**	**1b**	**1a^{2+}**
Empirical formula	$C_{42}H_{50}N_2O_2$	$C_{40}H_{46}N_2O_2Sb_2F_{12}$
Formula weight	614.84	627.84
Temperature/K	170.00(10)	170.00(10)
Crystal system	triclinic	monoclinic
Space group	P-1	C2/c
a / Å	5.8080(7)	40.823(2)
b / Å	9.2231(12)	9.4048(4)
c / Å	16.3047(10)	17.7684(7)
α / °	97.258(8)	90
β / °	100.154(7)	98.280(5)
γ / °	90.988(10)	90
Volume / Å^3	852.14(16)	6750.7(5)
Z	1	8
ρ_{calc}g / cm^3	1.198	1.235
μ / mm^{-1}	0.559	10.930
F (000)	332.0	2488.0
Crystal size / mm^3	0.26 × 0.09 × 0.07	0.36 × 0.12 × 0.11
Radiation	CuKα (λ = 1.54184)	CuKα (λ = 1.54184)
2θ range for data collection / °	9.674 to 134.056	8.756 to 134.15
Index ranges	-6 ≤ h ≤ 6, -11 ≤ k ≤ 11, -19 ≤ l ≤ 19	-48 ≤ h ≤ 46, -8 ≤ k ≤ 11, -19 ≤ l ≤ 21
Reflections collected	18349	31910
Independent reflections	2966 R_{int} = 0.1276, R_{sigma} = 0.0780	5979 R_{int} = 0.1603, R_{sigma} = 0.0896
Data / restraints / parameters	2966/0/215	5979/0/268
Goodness-of-fit on F^2	1.093	1.007
Final R indexes [I >= 2σ (I)]	R_1 = 0.1112, wR_2 = 0.3078	R_1 = 0.0825, wR_2 = 0.2163
Final R indexes [all data]	R_1 = 0.1254, wR_2 = 0.3191	R_1 = 0.1068, wR_2 = 0.2312
Largest diff. peak / hole / e Å^{-3}	0.42/-0.30	2.06/-0.73
CCDC	2104547	2132542

4. Lewis acid-base stimuli-responsive properties

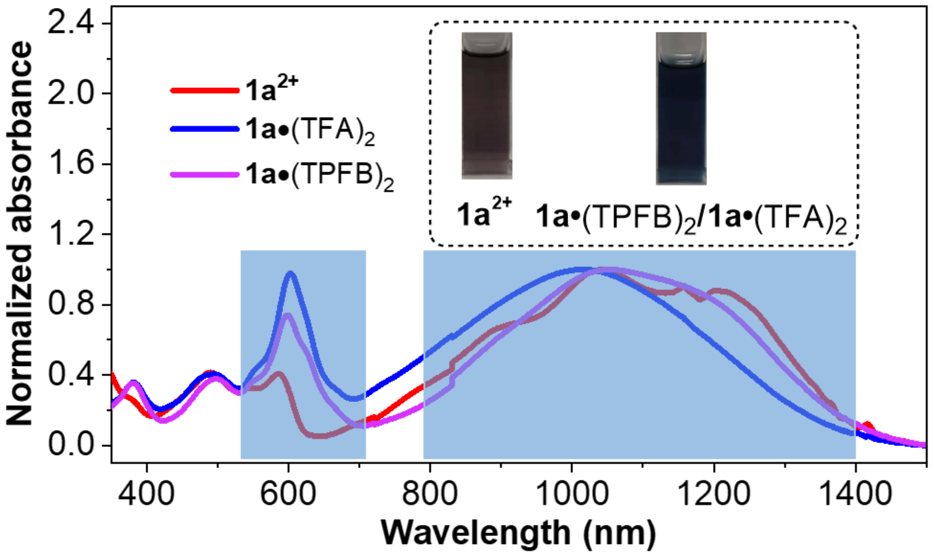

Supplementary Fig. 14: Normalized absorption spectra of $\mathbf{1a^{2+}}$, **1a•**(TPFB)$_2$ and **1a•**(TFA)$_2$. Inserted are the photos of the solutions. The main different parts of the absorption curves of $\mathbf{1a^{2+}}$, **1a•**(TPFB)$_2$ and **1a•**(TFA)$_2$ are marked with blue boxes.

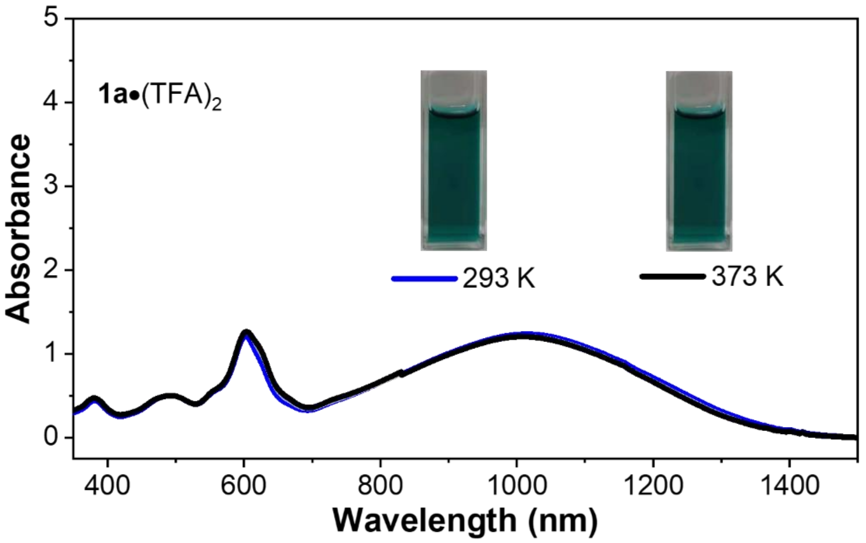

Supplementary Fig. 15: Variable temperature absorption of **1a•**(TFA)$_2$.

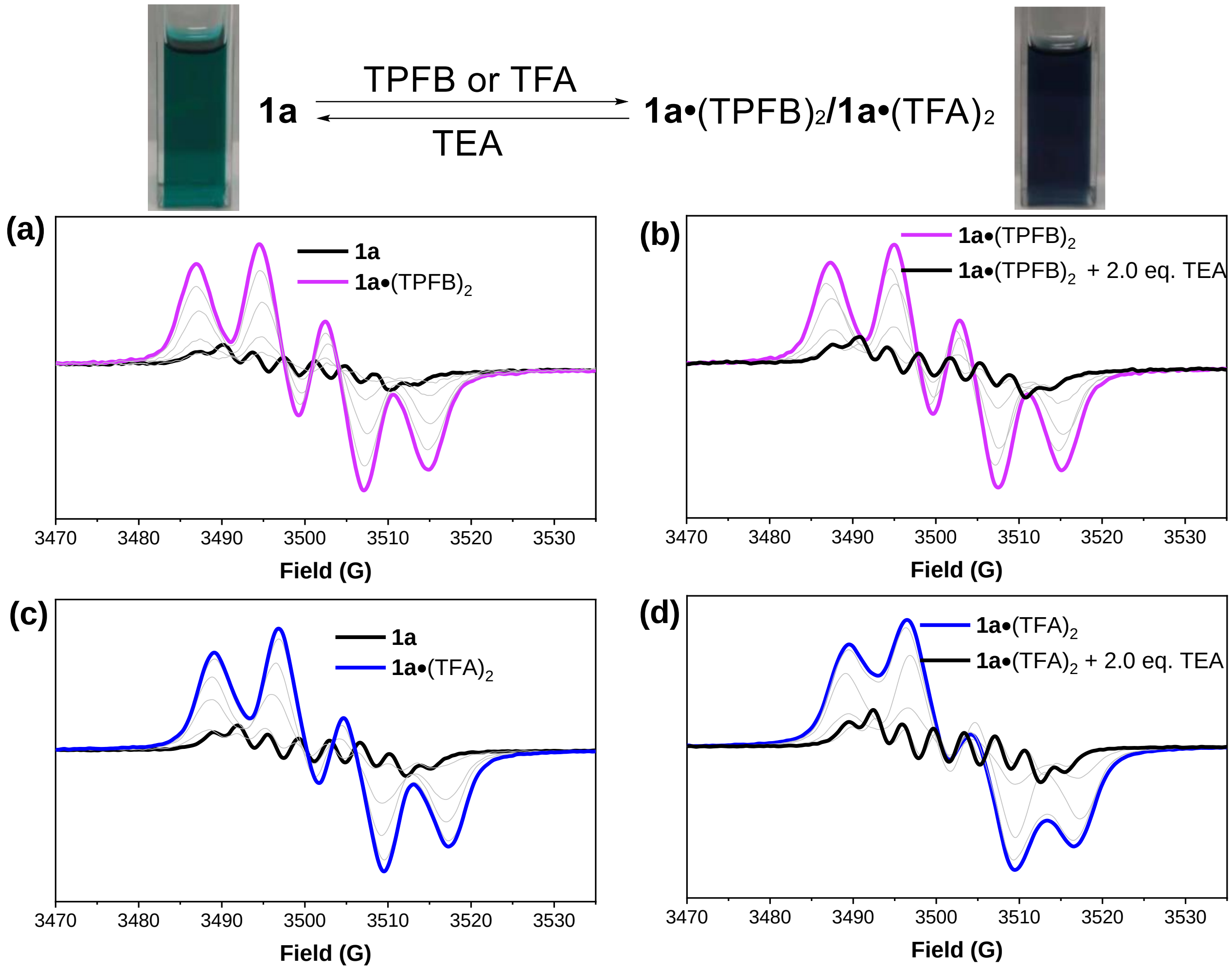

Supplementary Fig. 16: Schematic illustration of the reversible Lewis acid-base reaction between **1a** and corresponding acids. EPR spectra change upon (a) addition of TPFB to a solution of **1a** in CH_2Cl_2, (b) addition of TEA to a solution of **1a•**(TPFB)$_2$ in CH_2Cl_2, (c) addition of TFA to a solution of **1a** in CH_2Cl_2 and (d) addition of TEA to a solution of **1a•**(TFA)$_2$ in CH_2Cl_2 (14 μM).

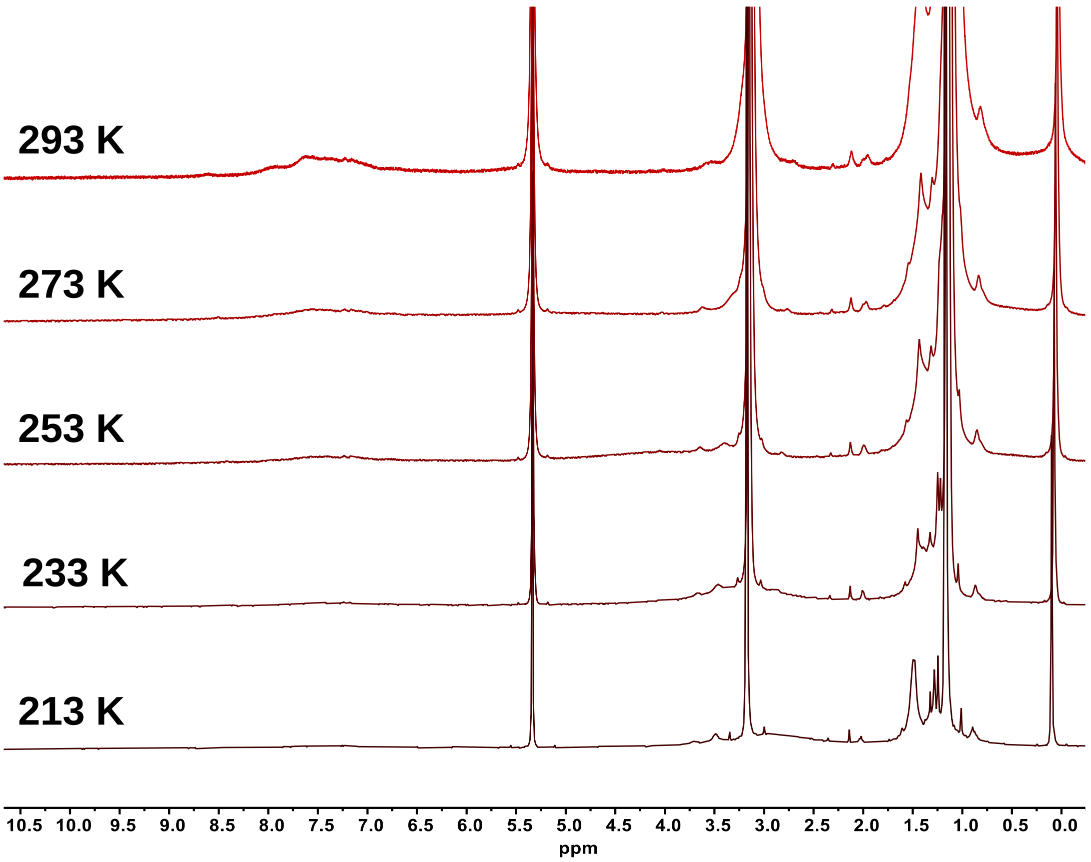

Supplementary Fig. 17: Variable-temperature ^{1}H NMR (600 MHz, CD_2Cl_2) spectra of **1a•**(TPFB)$_2$ from 213 K to 293 K.

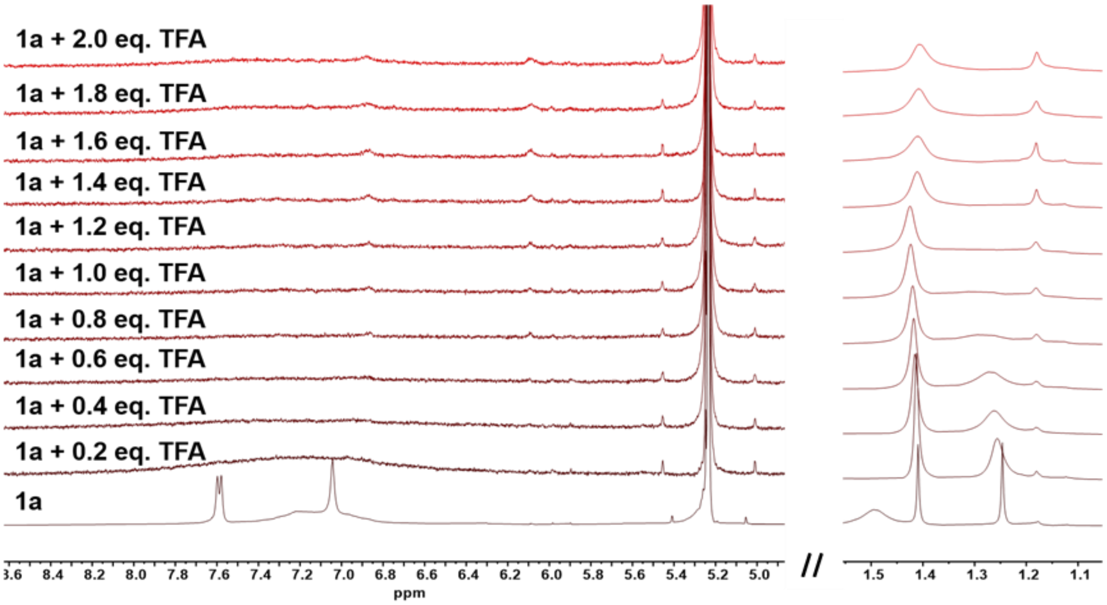

Supplementary Fig. 18: ^{1}H NMR (600 MHz, CD_2Cl_2, 298 K) spectra of titration of trifluoroacetate (TFA) into a solution of **1a** in CD_2Cl_2.

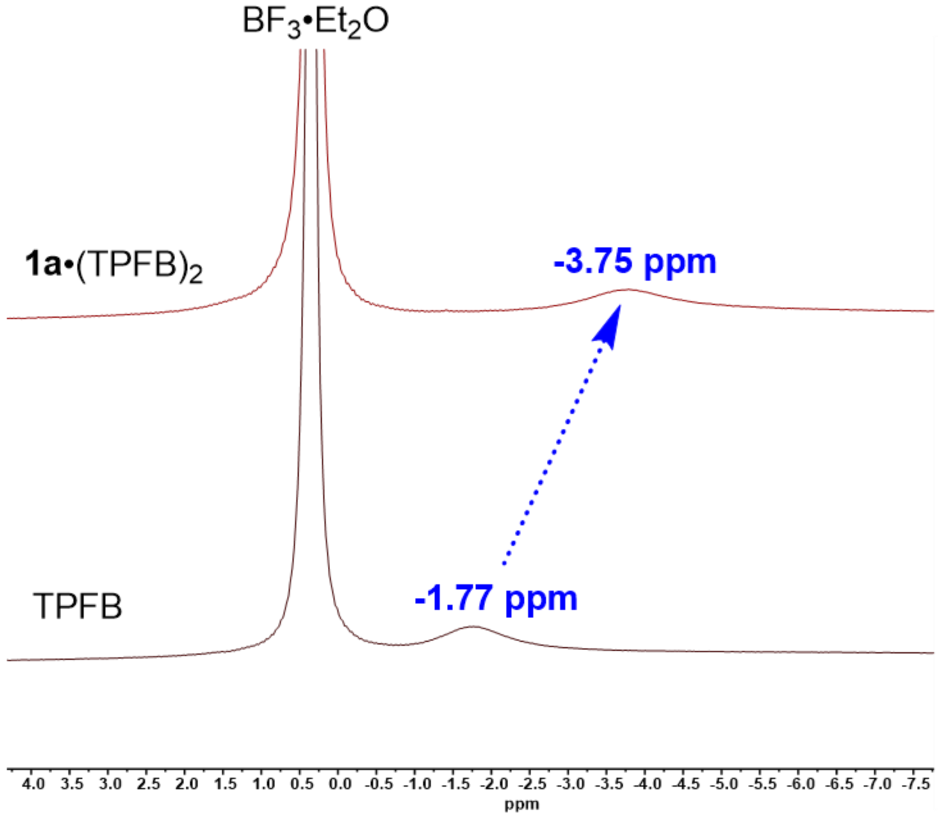

Supplementary Fig. 19: ^{11}B NMR (500 MHz, CD_2Cl_2, 298 K) spectra of **1a•**(TPFB)$_2$ and TPFB.

In-situ ^{11}B NMR spectra showed that after added **1a** to the TPFB in CD_2Cl_2, the boron signal of TPFB shifted from -1.17 ppm to -3.54 ppm, indicating the coordination between **1a** and TPFB. $BF_3 \cdot Et_2O$ as internal standard was used to guarantee the accuracy of the change in chemical shift.

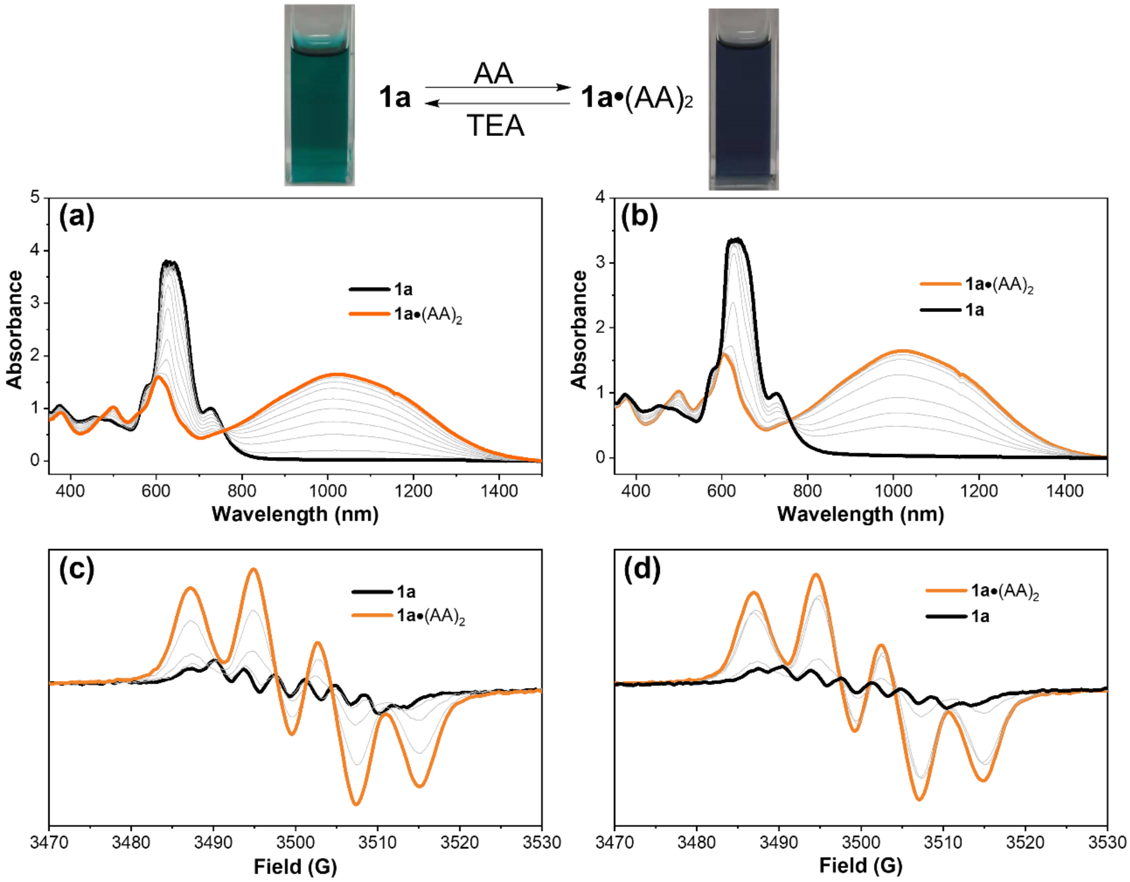

Supplementary Fig. 20: Schematic illustration of the reversible Lewis acid-base reaction between **1a** and glacial acetic acid (AA: glacial acetic acid). Change absorption upon (a) addition of AA to a solution of **1a** in CH_2Cl_2, (b) addition of TEA to a solution of **1a•**$(AA)_2$ in CH_2Cl_2 (14 μM), Lewis acid-base reaction between **1a** and glacial acetic acid (AA: glacial acetic acid). EPR spectra change upon (c) addition of AA to a solution of **1a** in CH_2Cl_2 and (d) addition of TEA to a solution of **1a•**$(TFA)_2$ in CH_2Cl_2 (14 μM).

Supplementary Table 5: Summary for the parameters of Bleaney-Bowers equation fitting.[a]

	$2J/k_B$	ρ	Statistics
	Value	Value	R^2
**1a**	-581.914	0.017	0.99253
**1a•**$(TPFB)_2$	-377.745	0.070	0.99564

[a] $\chi_p = \chi_{mol} - \chi_d^1$, χ_d: diamagnetic susceptibility (χ_d of **1a**: -425×10^{-6} emu mol^{-1}, χ_d of **1a•**$(TPFB)_2$: -846×10^{-6} emu mol^{-1}).

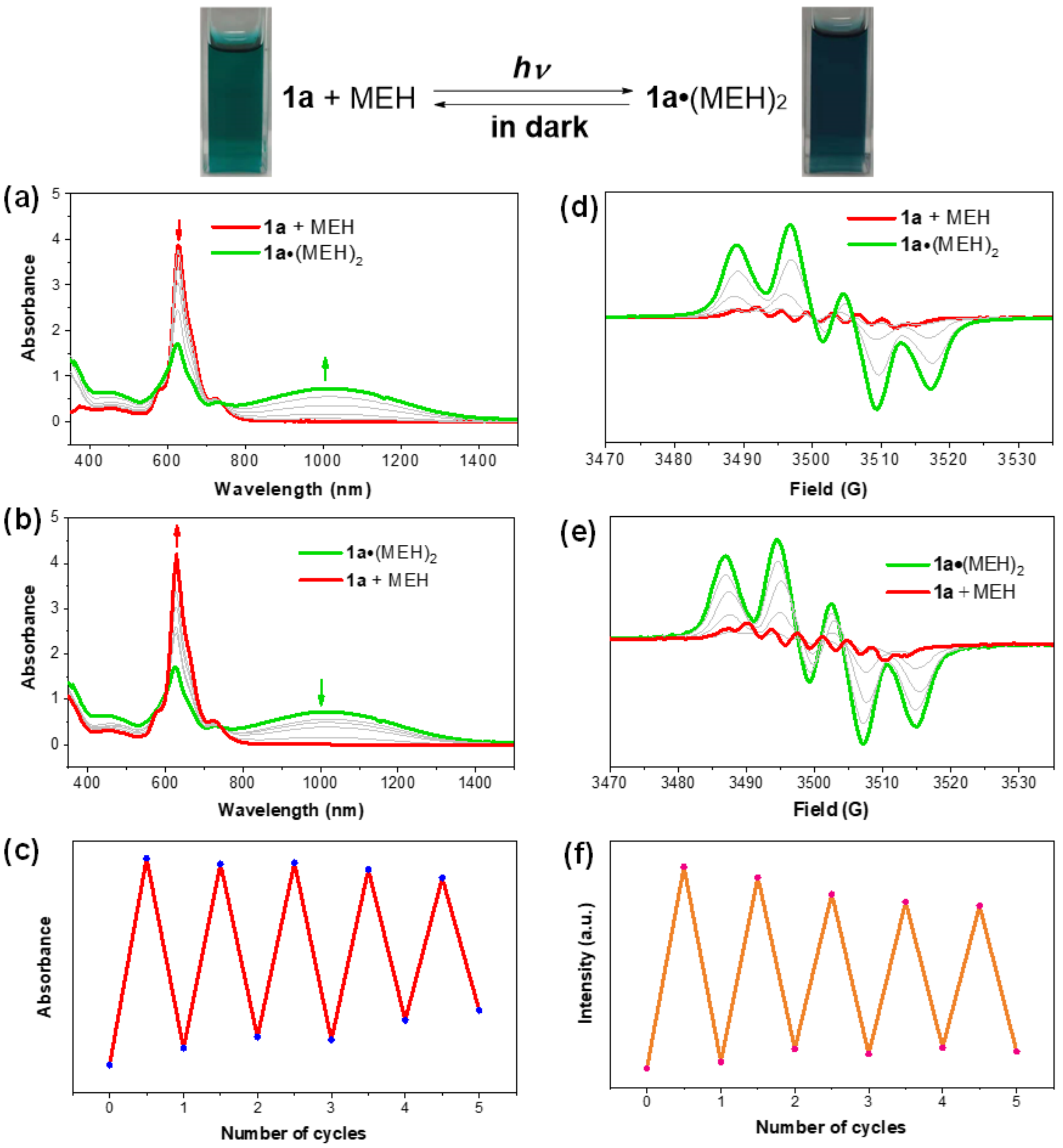

Supplementary Fig. 21: Light-controlled reversible modulation of UV-vis-NIR absorption and diradical character of **1a** (14 μM) by reversible Lewis acid-base reaction. Spectral absorption change of (a) the mixture of **1a** and MEH (mole ratio **1a**: MEH = 1: 5) irradiated by 365 nm light in CH_2Cl_2 for 150 s, then (b) keep the mixture in dark environment for 12 minutes, and (c) cycles under irradiation and in the dark. EPR spectra change of (d) the mixture of **1a** and MEH (mole ratio **1a**: MEH = 1: 5) irradiated by 365 nm light in CH_2Cl_2 for 150 s, then (e) keep the mixture in dark environment for 12 minutes, and (f) cycles under irradiation and in the dark.

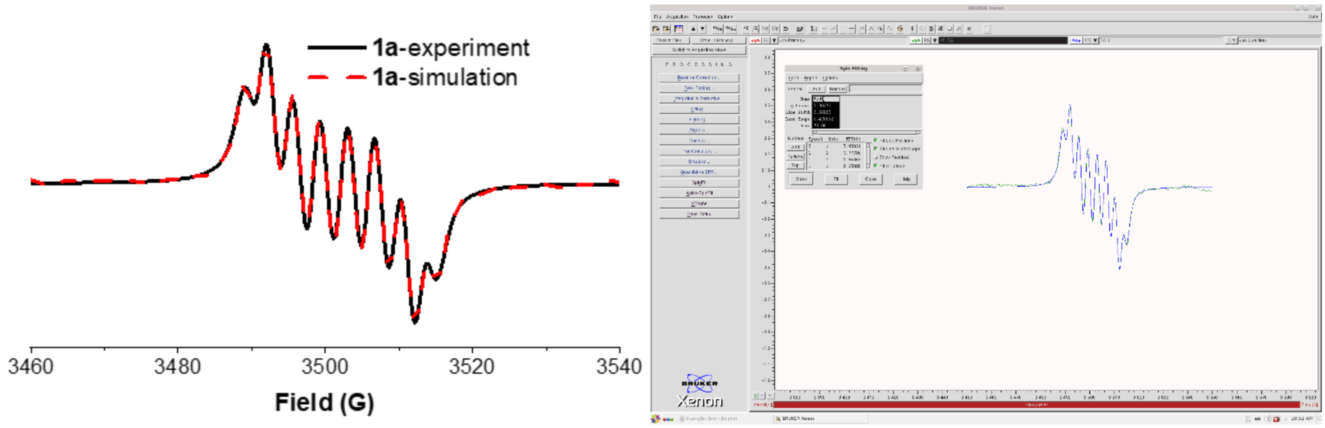

Supplementary Fig. 22: Experimental and simulated EPR spectra of **1a** in CH_2Cl_2 (data processing (left) and screenshot (right) from the Bruker SpinFit software). The fitting parameters for the spectral simulation are: *g* = 2.0024, A_N = 7.408 G, A_{Ha} = 3.777 G, A_{Hb} = 2.853 G and A_{Hc} = 2.688 G.

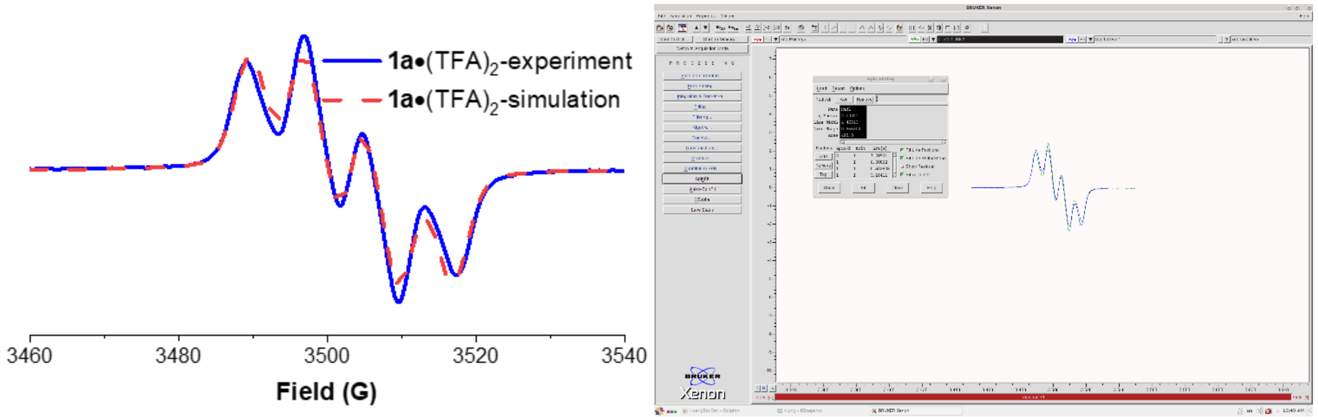

Supplementary Fig. 23: Experimental and simulated EPR spectra of **1a•**(TFA)2 in CH_2Cl_2 (data processing (left) and screenshot (right) from the Bruker SpinFit software). The fitting parameters for the spectral simulation are: *g* = 2.0032, A_N = 7.118 G, A_{Ha} = 6.681 G, A_{Hb} = 0.504 G and A_{Hc} = 3.184 G.

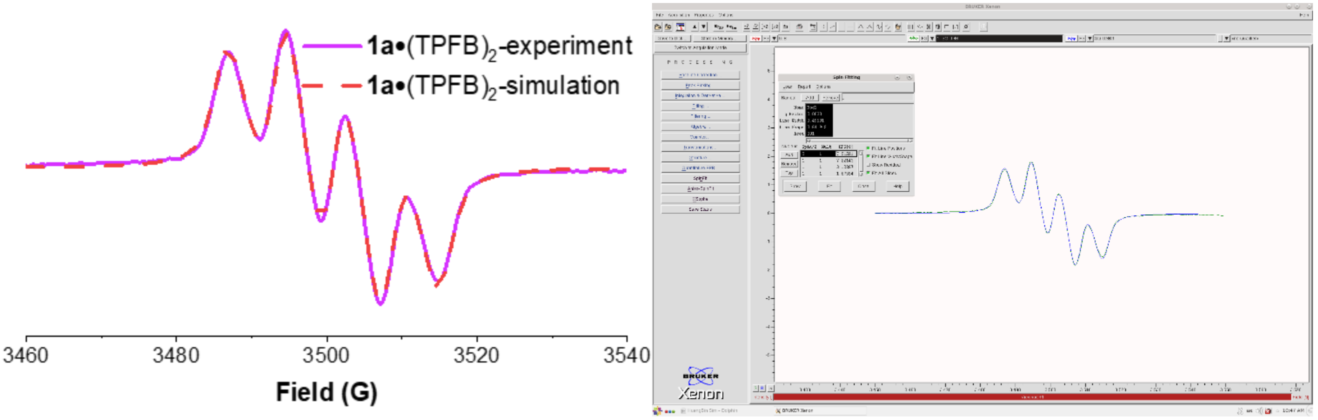

Supplementary Fig. 24: Experimental and simulated EPR spectra of **1a•**(TPFB)2 in CH_2Cl_2 (data processing (left) and screenshot (right) from the Bruker SpinFit software). The fitting parameters for the spectral simulation are: *g* = 2.0033, A_N = 7.215 G, A_{Ha} = 7.221 G, A_{Hb} = 3.123 G and A_{Hc} = 1.676 G.

5. Theoretical calculation

Theoretical calculations were performed with the Gaussian16 program suite[2]. All molecules geometry optimizations were performed by using ωB97XD exchange-correlation functional in conjunction with 6-31G(d,p) basis set[3,4]. Initial guess broken-symmetry (open-shell singlet) wavefunction was found with Guess=Mix, Nosymm and Stable=Opt keywords, then the Guess=Read keyword was used to optimize the broken-symmetry state geometry structure at the UωB97XD/6-31G(d,p) level. Frequency calculations were conducted to ensure that these structures were indeed local minima. Transition state structures were verified by frequency calculations and only one imaginary frequency was found in the transition state. Closed-shell wavefunction has an RHF→UHF instability and open-shell wavefunction is stable under the perturbations considered in stability test of wavefunction[5-7]. Single point energy was performed at the level of ωB97XD/def2-TZVP[8]. Time-dependent density functional theory (TD-DFT) calculations were performed at the level of ωB97XD/6-31G(d,p). Nucleus-independent chemical shift values ($NICS(1)_{ZZ}$) were calculated using the standard GIAO[9,10] at the level of ωB97XD/6-31G(d,p). Spin population calculations based on becke method divide atomic space. Electronic structure analyses were performed with the Multiwfn 3.8 (dev) code. The isosurface maps of spin density and were rendered by means of Visual Molecular Dynamics (VMD 1.9.3) software[11] based on the files exported from Multiwfn.

The singlet-triplet energy gap ΔE_{S-T} was calculated as:

$$\Delta E_{S-T} = E_{BS} - E_T$$

where the E_{BS} and E_T were the energy of local minima structure in broken-symmetry state and triplet state, respectively.

Unrestricted natural orbital (UNO) occupation number calculations were done by unrestricted hartree-fock (UHF) and unrestricted density functional theory (UDFT), the diradical character (y_0) was calculated according to Yamaguchi's scheme as[12]:

$$y_0 = 1 - \frac{2T_0}{1 + T_0^2}$$

$$T_0 = \frac{n_{HONO} - n_{LUNO}}{2}$$

Where n indicates the occupation number of the highest occupied natural orbital (HONO) and the lowest unoccupied natural orbital (LUNO), a molecule with $y_0 = 0$ means a closed-shell structure, whereas a molecule with $y_0 = 1$ means a pure diradical structure.

5.1 Structure simulation

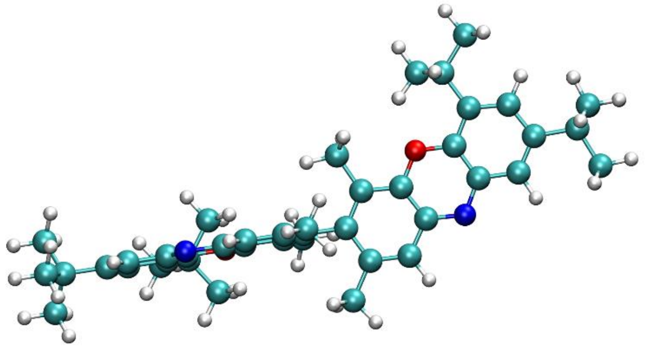

Supplementary Fig. 25: Calculated geometry structure of **1d**.

5.2 Conformational isomer interconversion pathway

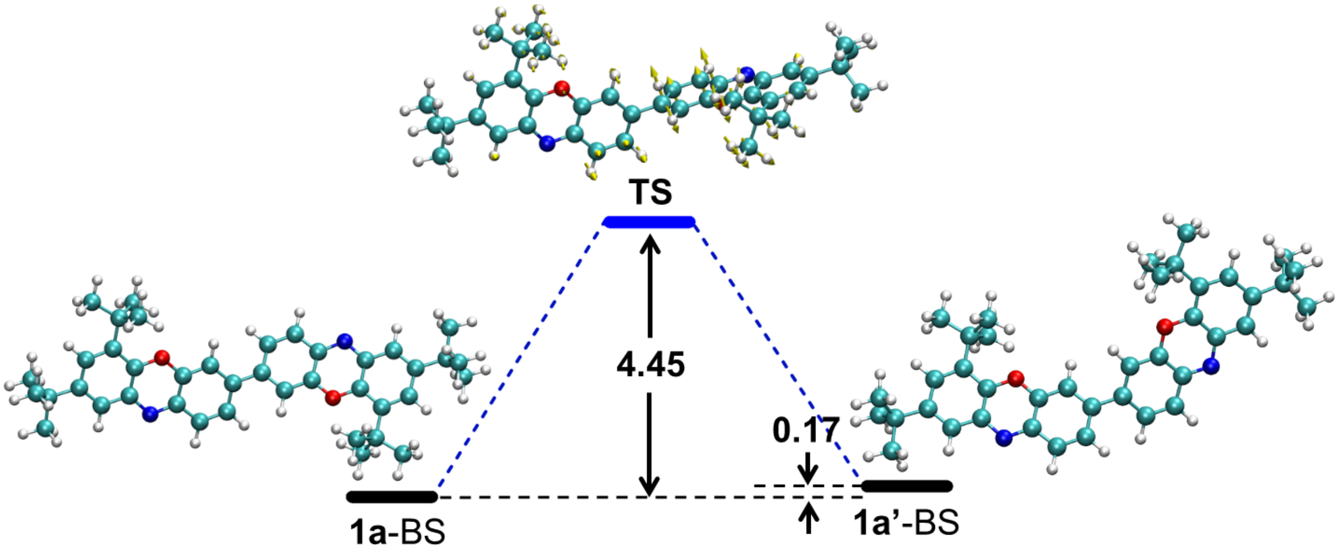

Supplementary Fig. 26: Calculated conformational isomer interconversion pathways of **1a** and **1a'**. Yellow arrow was the imaginary frequency vibration vector of the transition state. All units were in kcal mol^{-1}.

5.3 Nucleus independent chemical shift (NICS) values

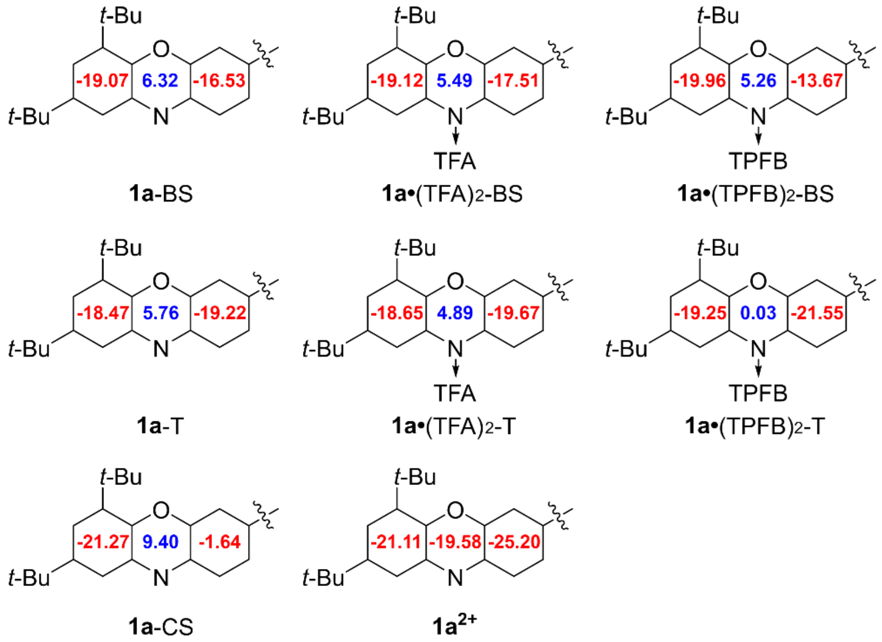

Supplementary Fig. 27 Calculated $NICS(1)_{zz}$ values of **1a**-BS, **1a**-T, **1a**-CS, **1a**$^{2+}$, **1a**•$(TFA)_2$-BS, **1a**•$(TFA)_2$-T, **1a**•$(TPFB)_2$-BS and **1a**•$(TPFB)_2$-T at the level of ωB97XD/6-31G(d,p). Here $NICS(1)_{zz}$ value was the average of $NICS(1)_{zz}$ value and $NICS(-1)_{zz}$ value. Red negative values and blue positive values represent aromaticity and antiaromaticity, respectively.

5.4 Spin density and spin population

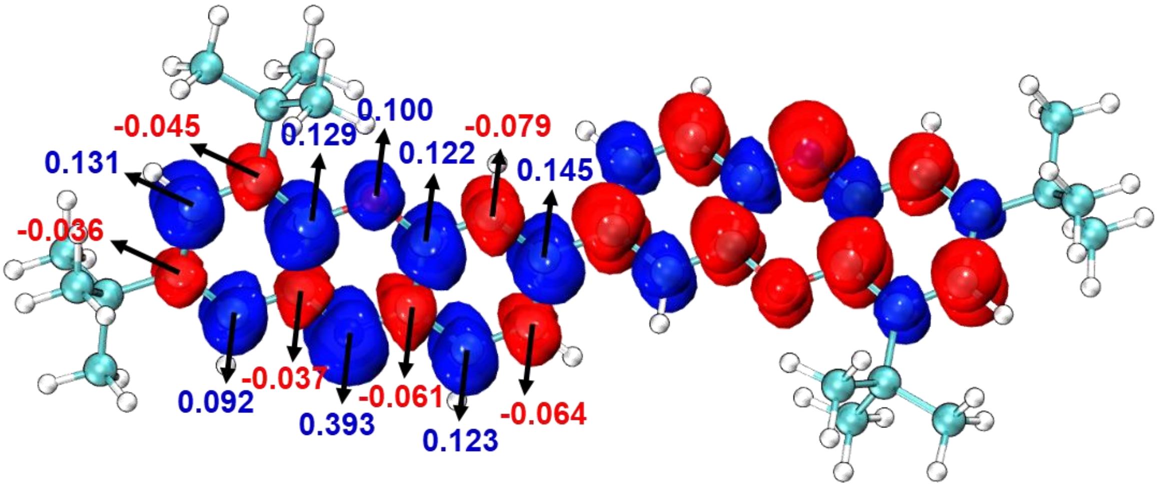

Supplementary Fig. 28: Spin density and spin population of **1a** in broken-symmetry state based on becke method at the level of ωB97XD/def2-TZVP. Isovalue was 0.002 a.u.

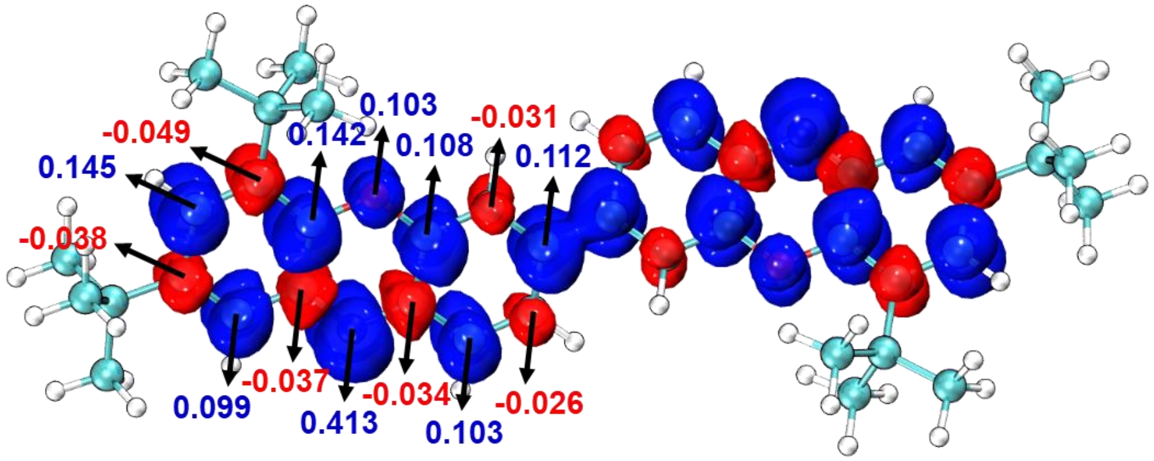

Supplementary Fig. 29: Spin density and spin population of **1a** in triplet state based on becke method at the level of ωB97XD/def2-TZVP. Isovalue was 0.002 a.u.

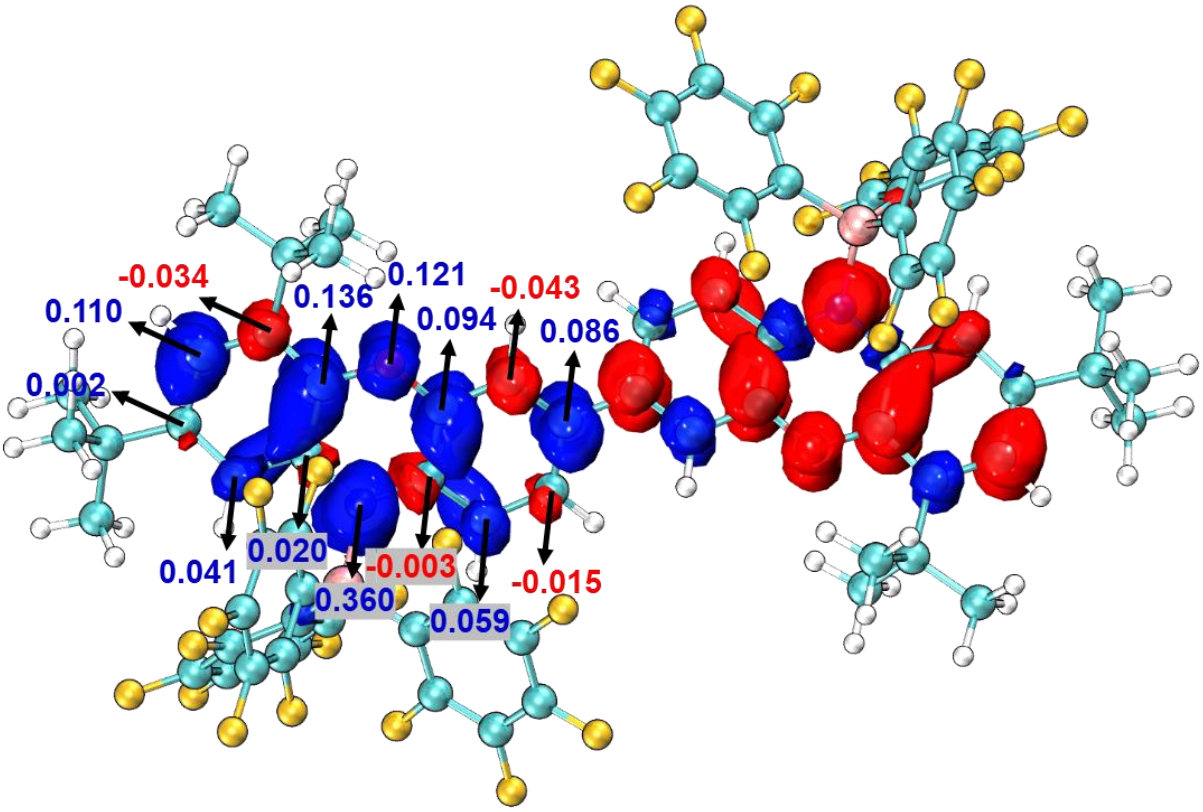

Supplementary Fig. 30: Spin density and spin population of **1a•**(TPFB)$_2$ in broken-symmetry state based on becke method at the level of ωB97XD/def2-TZVP. Isovalue was 0.002 a.u.

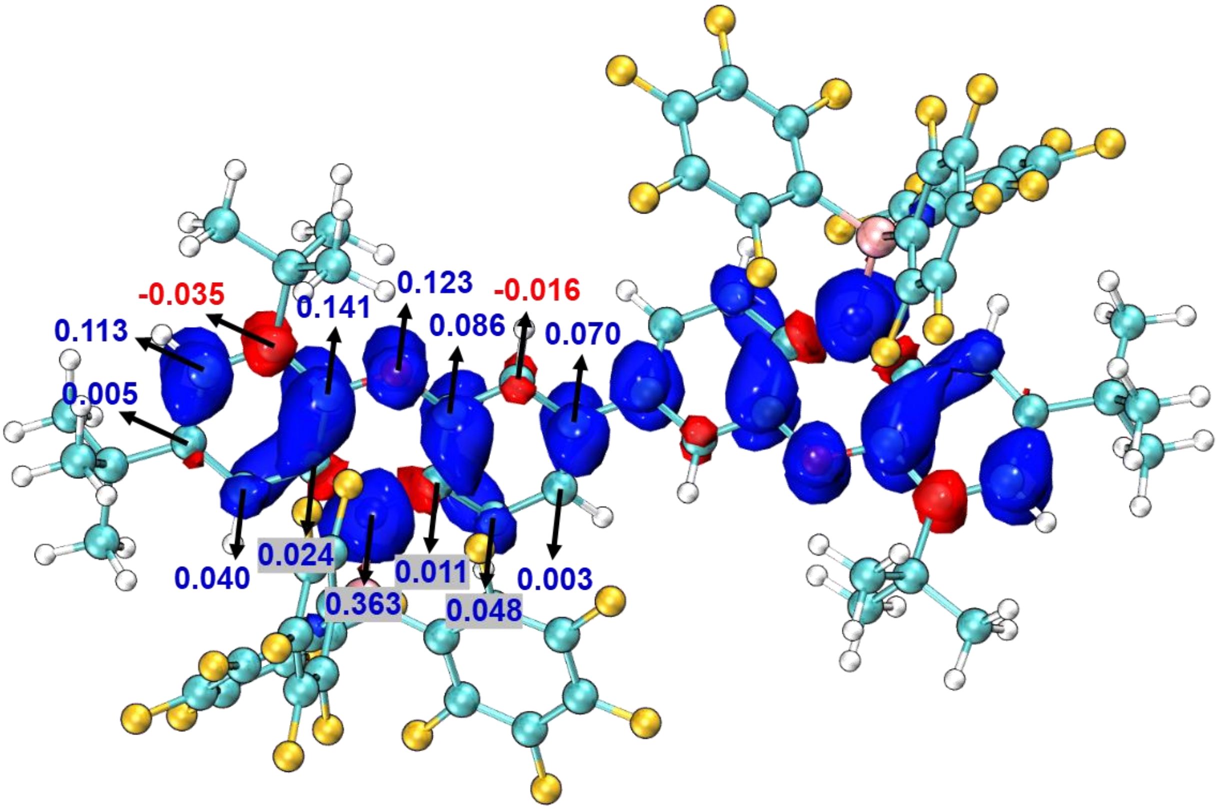

Supplementary Fig. 31: Spin density and spin population of **1a•**(TPFB)$_2$ in triplet state based on becke method at the level of ωB97XD/def2-TZVP. Isovalue was 0.002 a.u.

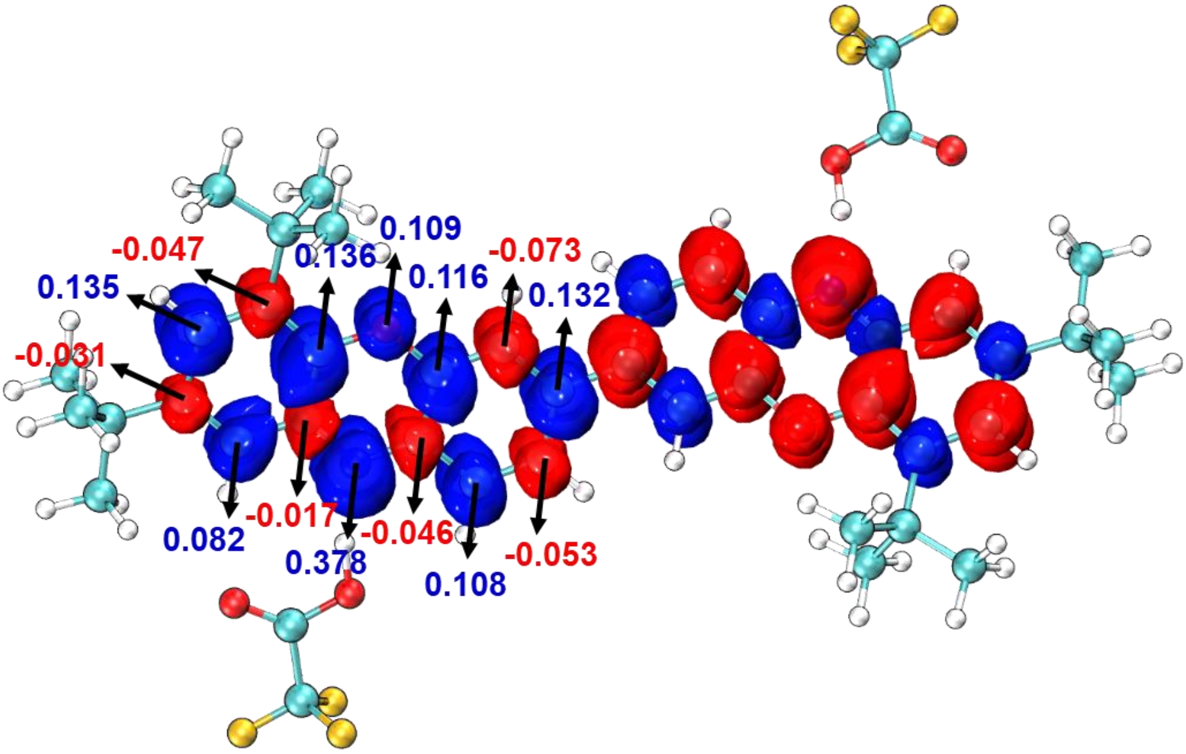

Supplementary Fig. 32: Spin density and spin population of **1a**•$(TFA)_2$ in broken-symmetry state based on becke method at the level of ωB97XD/def2-TZVP. Isovalue was 0.002 a.u.

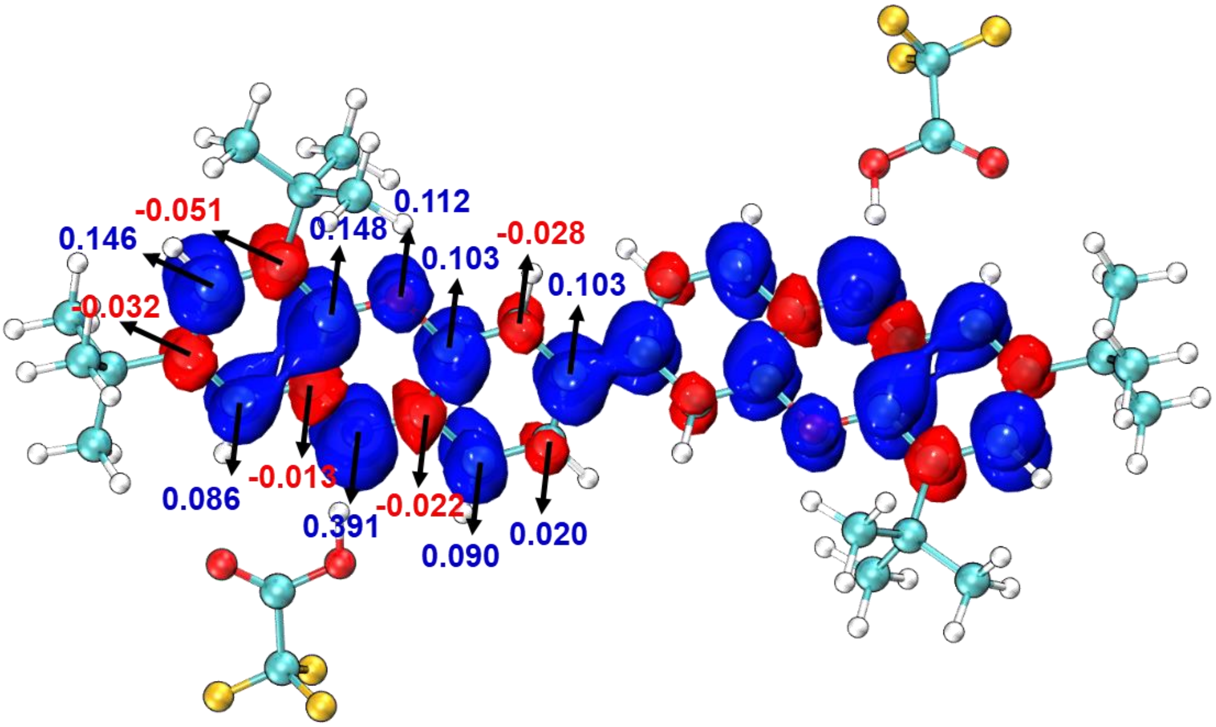

Supplementary Fig. 33: Spin density and spin population of **1a**•$(TFA)_2$ in triplet state based on becke method at the level of ωB97XD/def2-TZVP. Isovalue was 0.002 a.u.

5.5 Molecular thermochemistry properties

Supplementary Table 6: Stability of wavefunction, thermal correction to gibbs free energy (G_{corr}(T)), electronic energy (ε_{ele}), relative gibbs free energy (ΔG), and singlet-triplet energy gap ($\Delta E_{S\text{-}T}$) of all calculated moleculars. Gibbs free energy $G(\mathrm{T}) = \varepsilon_{ele} + G_{corr}(T)$, T = 298.15 K, P = 1 atm.

	**1a**-BS	**1a**-T	**1a**-CS	**1a**$^{2+}$	**1a**•$(TFA)_2$-BS	**1a**•$(TFA)_2$-T	**1a**•$(TPFB)_2$-BS	**1a**•$(TPFB)_2$-T
Stability	stable	stable	RHF→UHF	stable	stable	stable	stable	stable
G_{corr}(T)	0.701111	0.701531	0.702182	0.704692	0.753756	0.754534	0.949648	0.949330
ε_{ele}	-1811.98240977	-1811.97948062	-1811.96718675	-1811.45054163	-2865.79201417	-2865.78963687	-6229.15666562	-6229.15510485
G(T)	-1811.28129877	-1811.27794962	-1811.26500475	-1810.74584963	-2865.03825817	-2865.03510287	-6228.20701762	-6228.20577485
ΔG (a.u.)	0	0.00334915	0.01629402		0	0.00315530	0	0.00124277
ΔG (kcal mol^{-1})	Ground state	2.10	10.22		Ground state	1.98	Ground state	0.78
$\Delta E_{S\text{-}T}$	-2.10				-1.98		-0.78	

5.6 Diradical character

Supplementary Table 7: n_{HONO}, n_{LUNO} and y_0 values of **1a**, **1a•**(TFA)$_2$, **1a•**(TPFB)$_2$ and **1d** at the level of UHF and UDFT (UωB97XD/UCAM-B3LYP).

		**1a**	**1a•**(TFA)$_2$	**1a•**(TPFB)$_2$	**1d**
UHF	n_{HONO}	1.10747	1.10054	1.06987	1.00635
	n_{LUNO}	0.89253	0.89946	0.93013	0.99365
	y_0^{UHF}	0.787	0.801	0.861	0.987
UωB97XD	n_{HONO}	1.19267	1.17013	1.09738	1.01049
	n_{LUNO}	0.80733	0.82987	0.90262	0.98951
	$y_0^{U\omega B97XD}$	0.628	0.669	0.807	0.979
UCAM-B3LYP	n_{HONO}	1.20930	1.18632	1.10932	1.01145
	n_{LUNO}	0.79070	0.81368	0.89068	0.98855
	$y_0^{UCAM-B3LYP}$	0.599	0.640	0.784	0.977

5.7 Theoretical UV-Vis spectrum

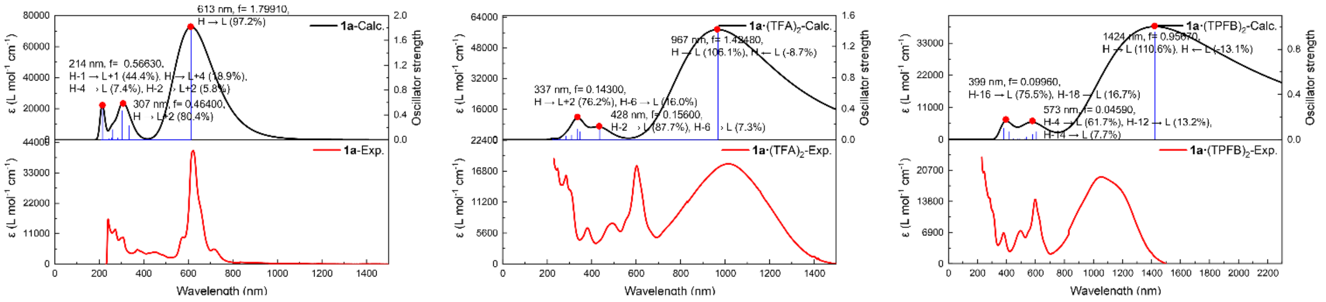

Supplementary Fig. 34: Theoretical (black solid curve) and experimental (red solid curve) UV-vis-NIR spectra of **1a**, **1a•**(TFA)$_2$ and **1a•**(TPFB)$_2$. The blue vertical line represents the oscillator strength.

6. ^{1}H NMR and ^{13}C NMR spectra of new compounds

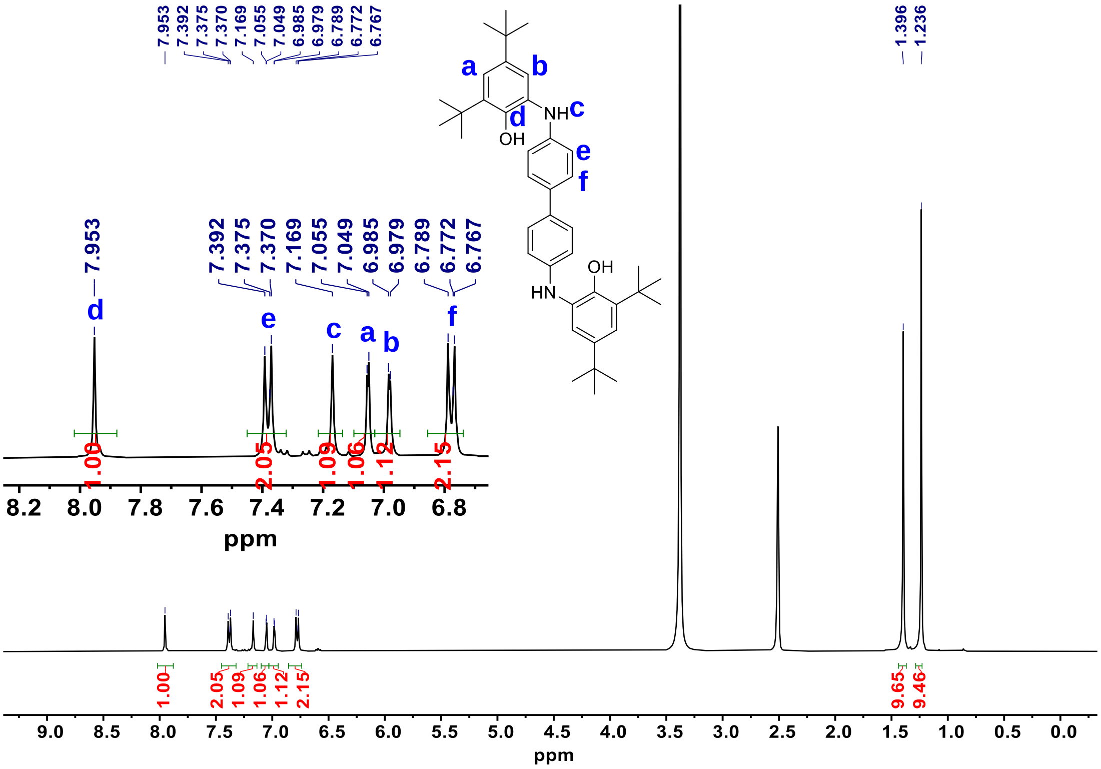

Supplementary Fig. 35: ^{1}H NMR (400 MHz, DMSO-d_6, 298 K) spectrum of **4a**.

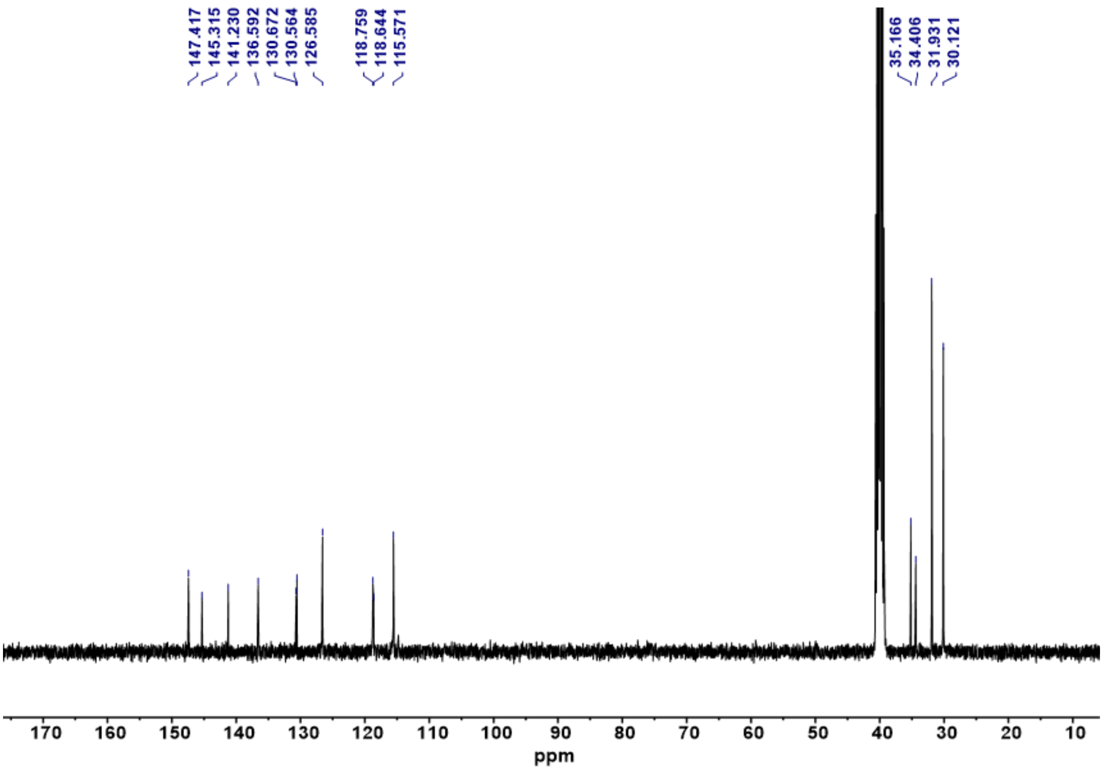

Supplementary Fig. 36: ^{13}C NMR (100 MHz, DMSO-d_6, 298 K) spectrum of **4a**.

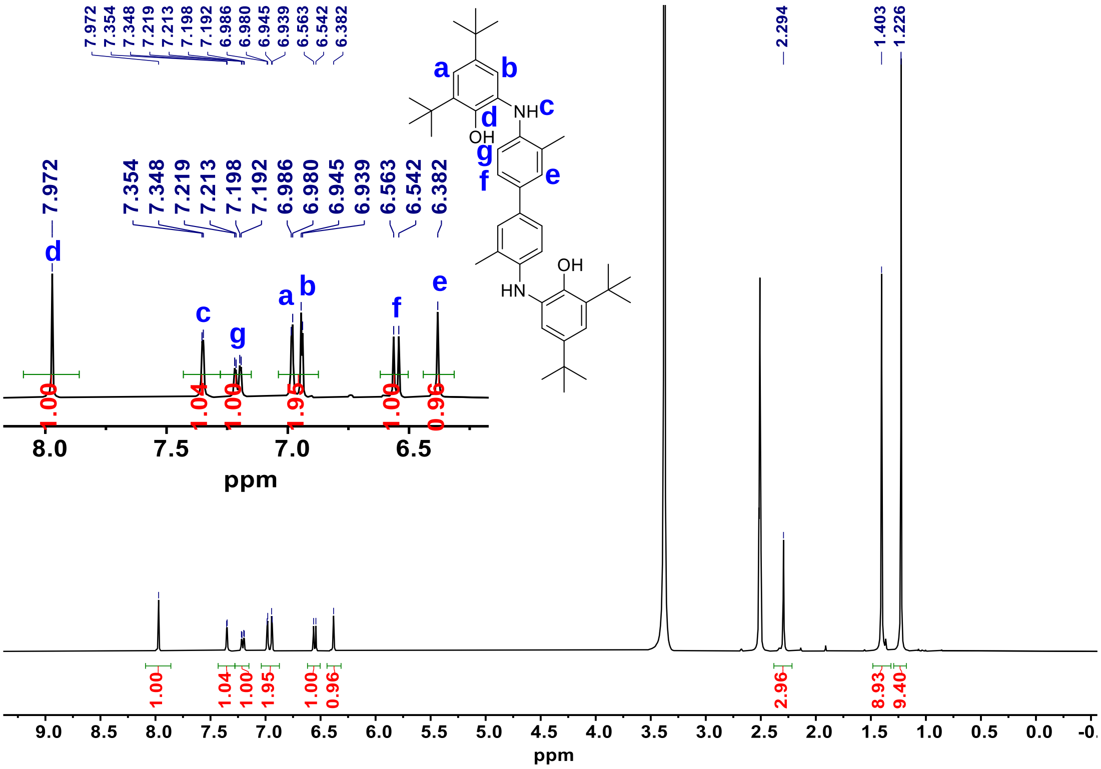

Supplementary Fig. 37: ^{1}H NMR (400 MHz, DMSO-d_6, 298 K) spectrum of **4b**.

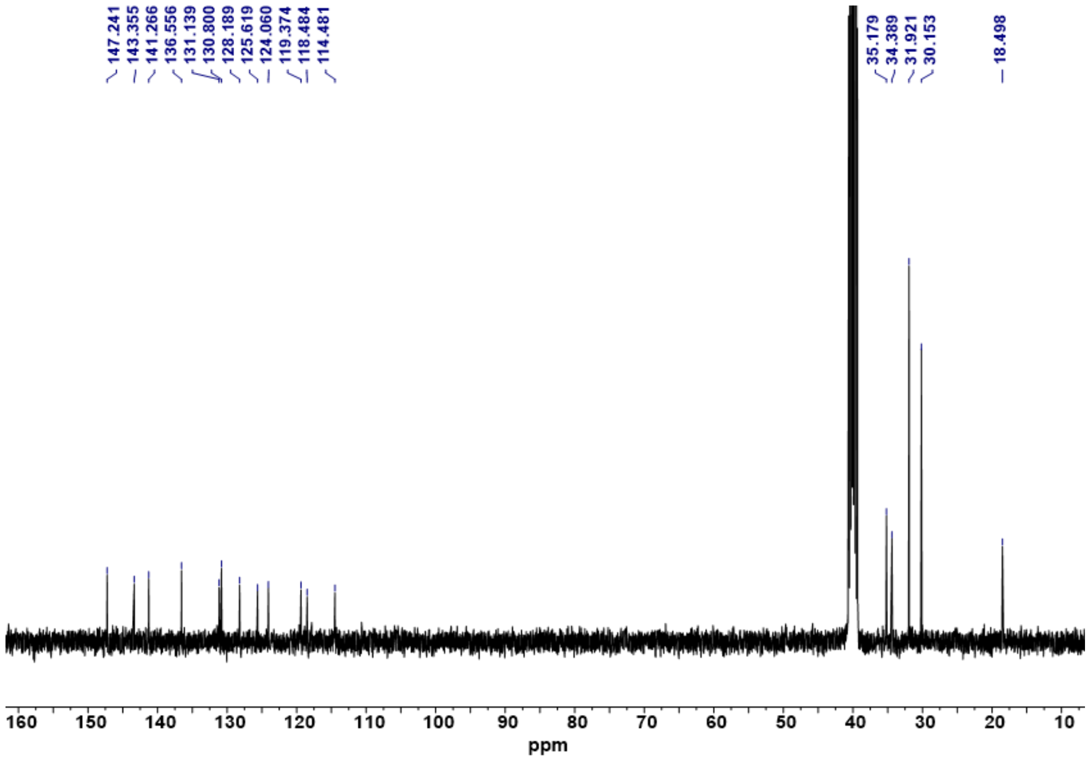

Supplementary Fig. 38: ^{13}C NMR (100 MHz, DMSO-d_6, 298 K) spectrum of **4b**.

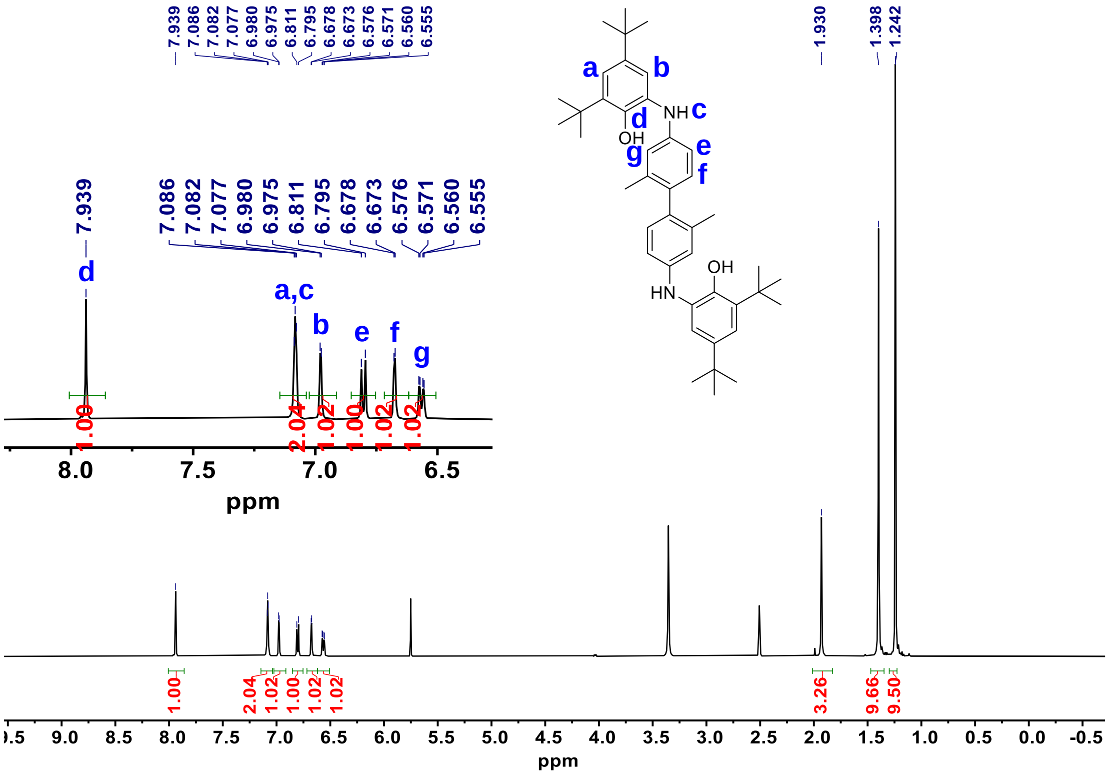

Supplementary Fig. 39: ^{1}H NMR (500 MHz, DMSO-d_6, 298 K) spectrum of **4c**.

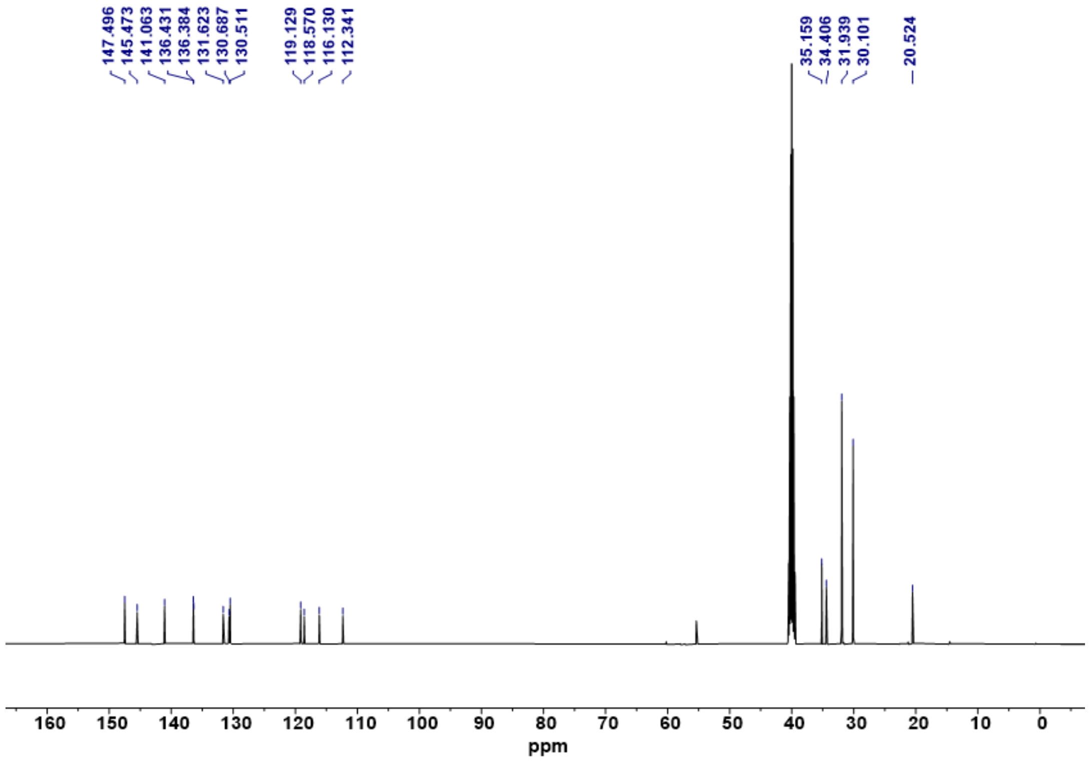

Supplementary Fig. 40: ^{13}C NMR (125 MHz, DMSO-d_6, 298 K) spectrum of **4c**.

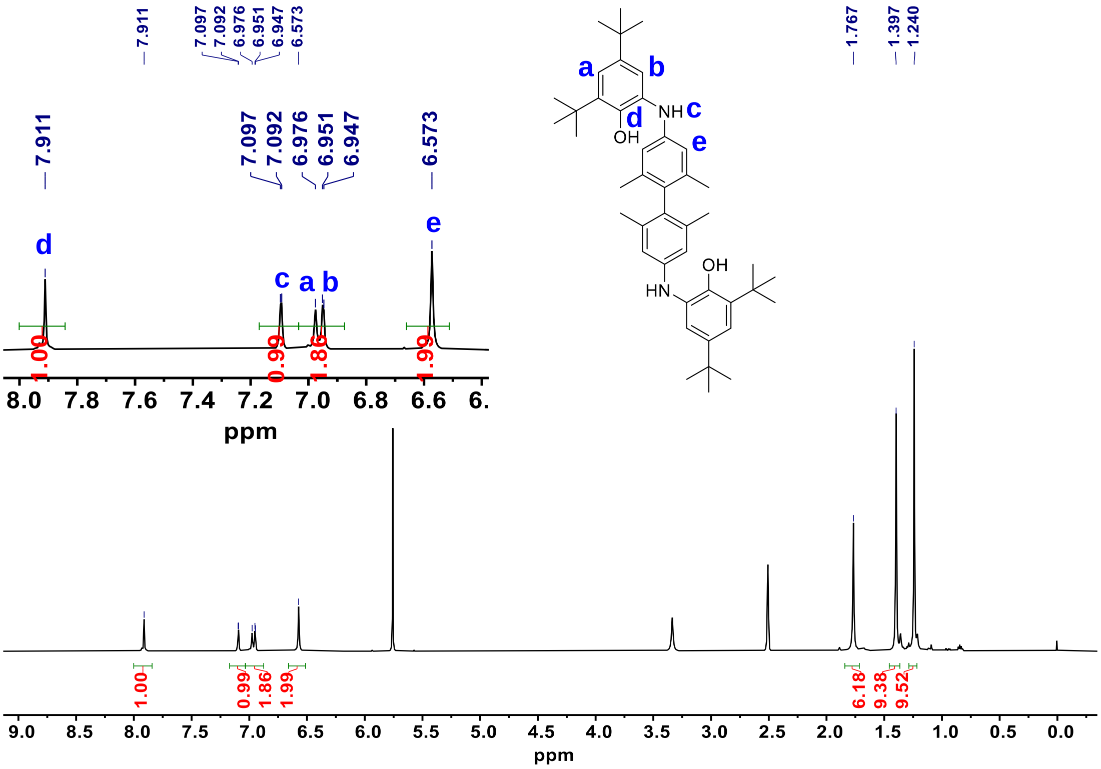

Supplementary Fig. 41: ^{1}H NMR (500 MHz, DMSO-d_6, 298 K) spectrum of **4d**.

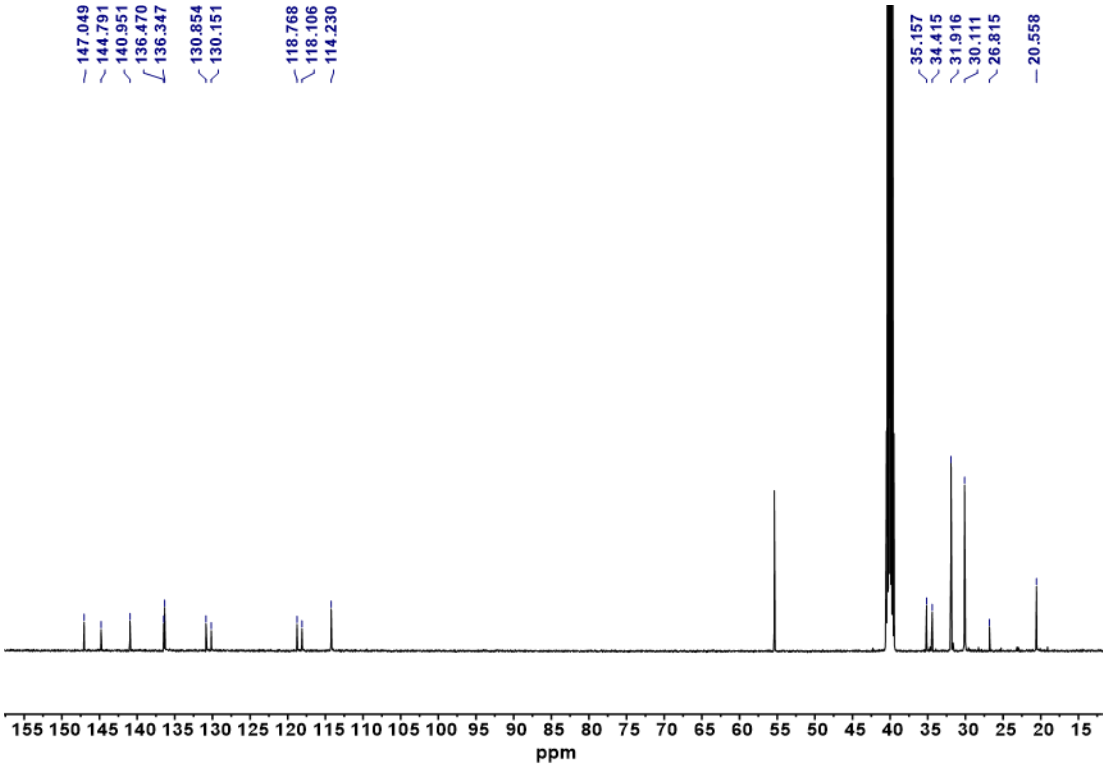

Supplementary Fig. 42: ^{13}C NMR (125 MHz, DMSO-d_6, 298 K) spectrum of **4d**.

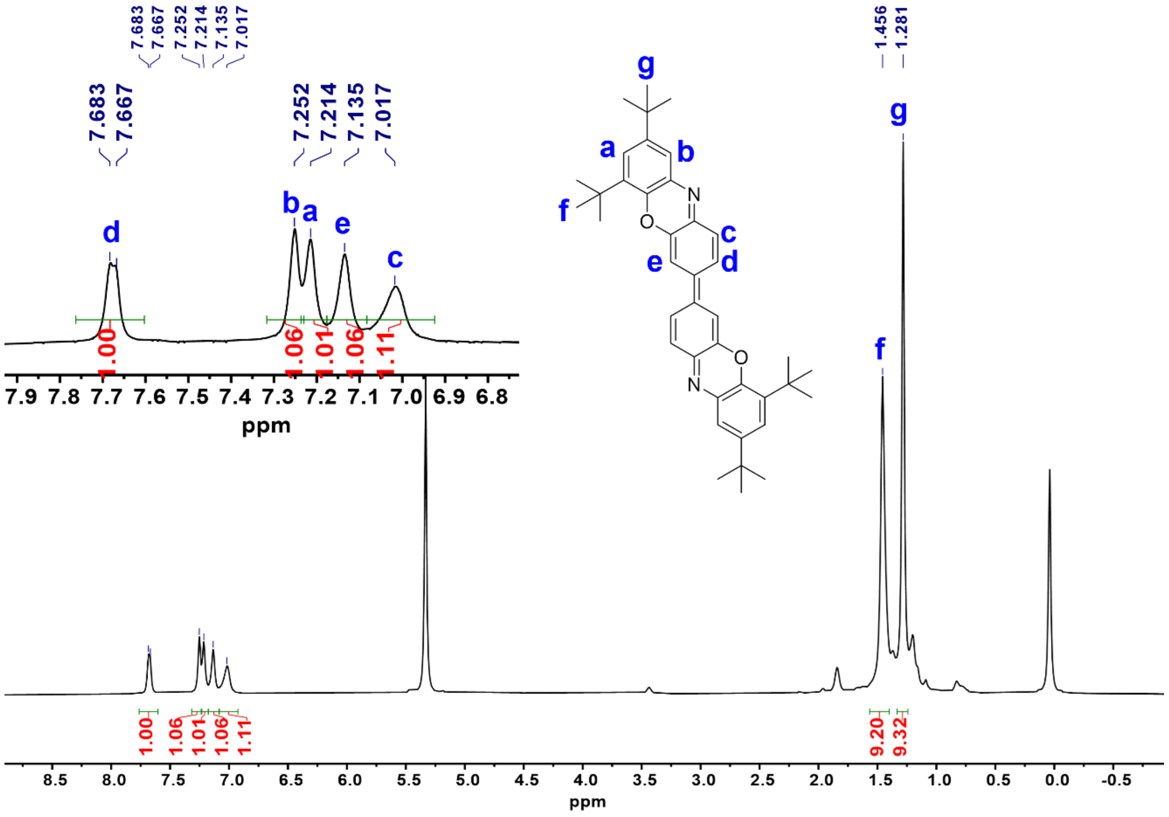

Supplementary Fig. 43: ^{1}H NMR (600 MHz, CD_2Cl_2, 233 K) spectrum of **1a**.

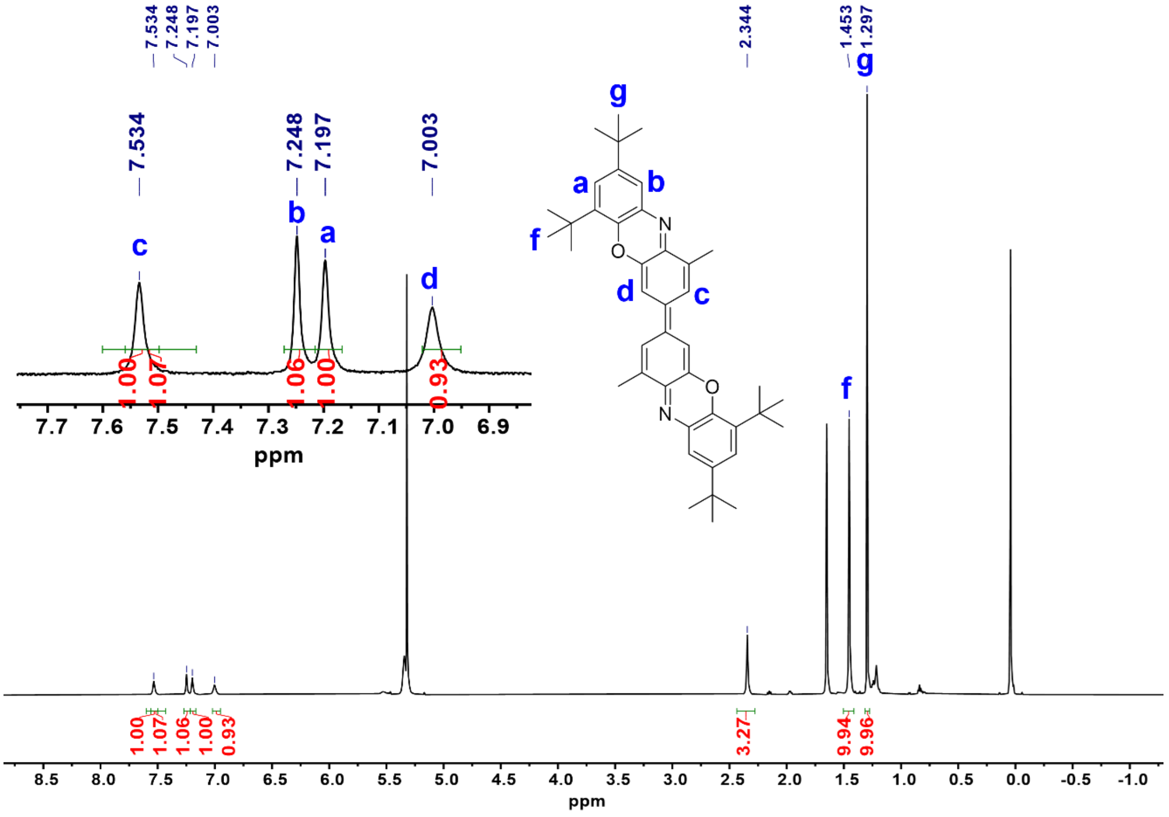

Supplementary Fig. 44: ^{1}H NMR (600 MHz, CD_2Cl_2, 248 K) spectrum of **1b**.

7. Mass spectra of new compounds

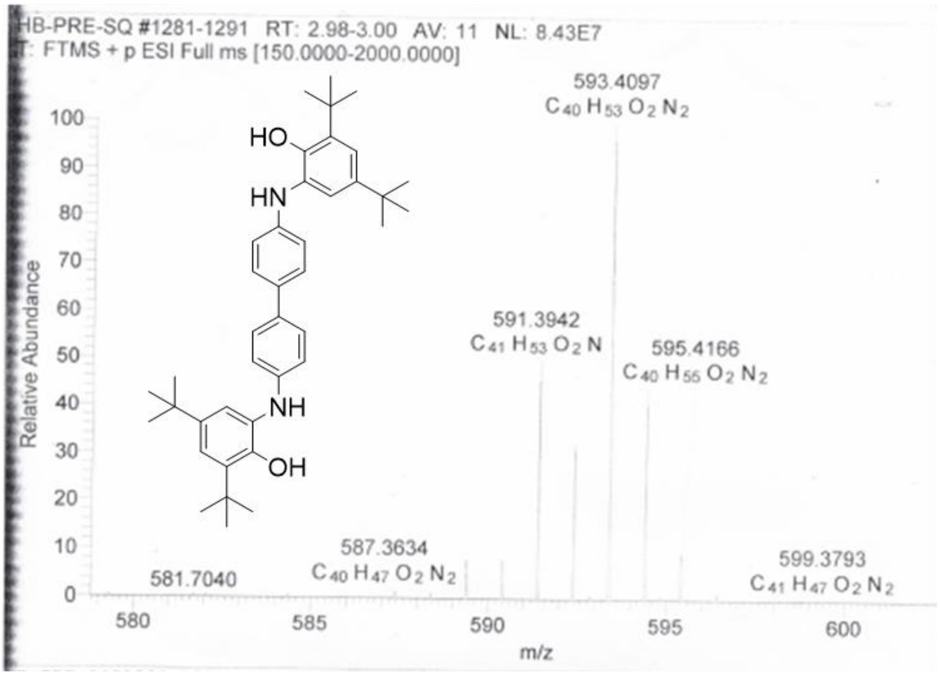

Supplementary Fig. 45: The mass spectrum of compound **4a**.

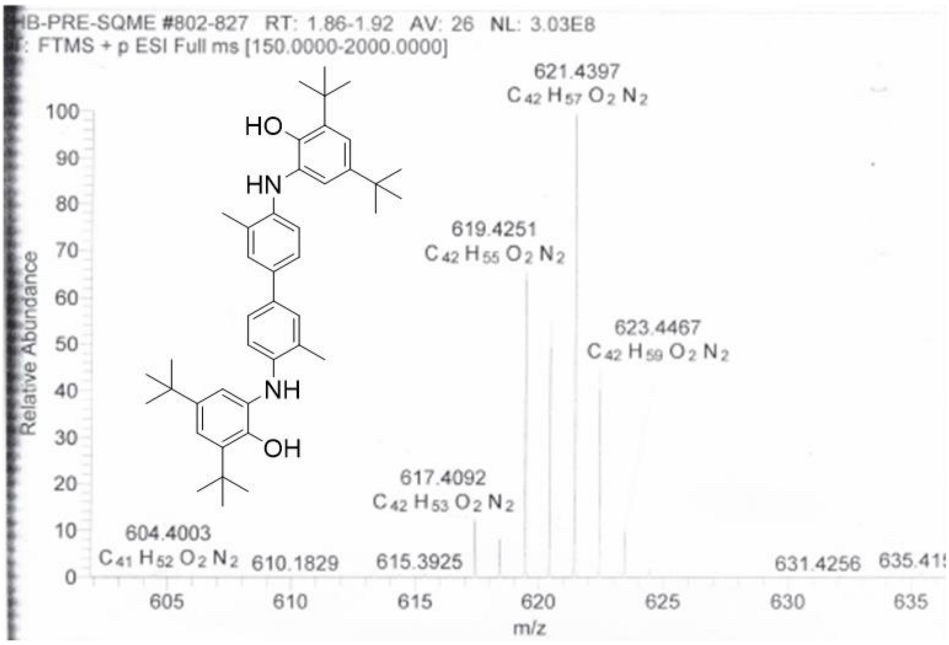

Supplementary Fig. 46: The mass spectrum of compound **4b**.

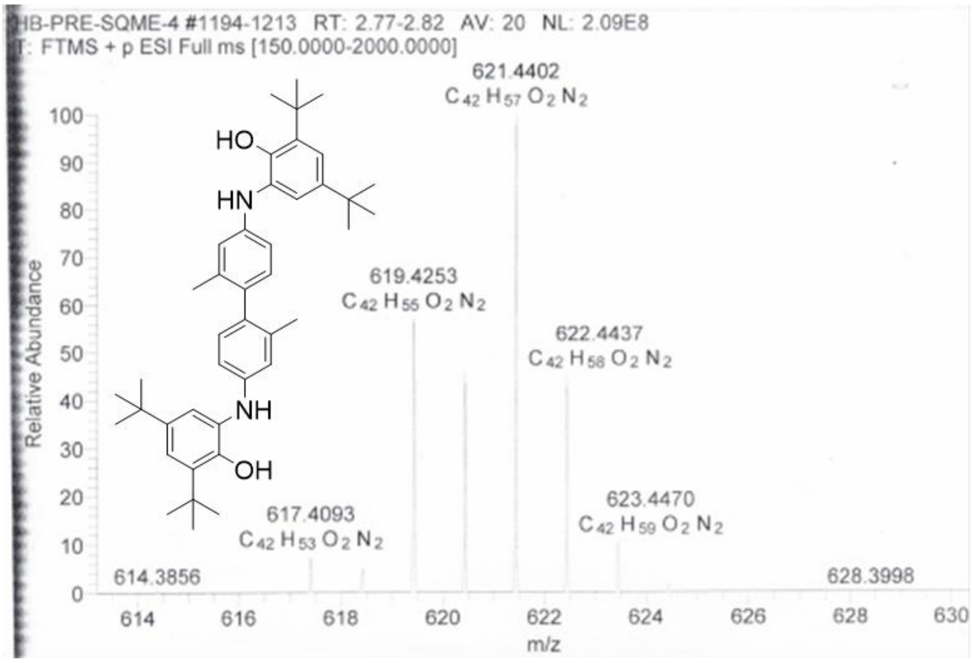

Supplementary Fig. 47: The mass spectrum of compound **4c**.

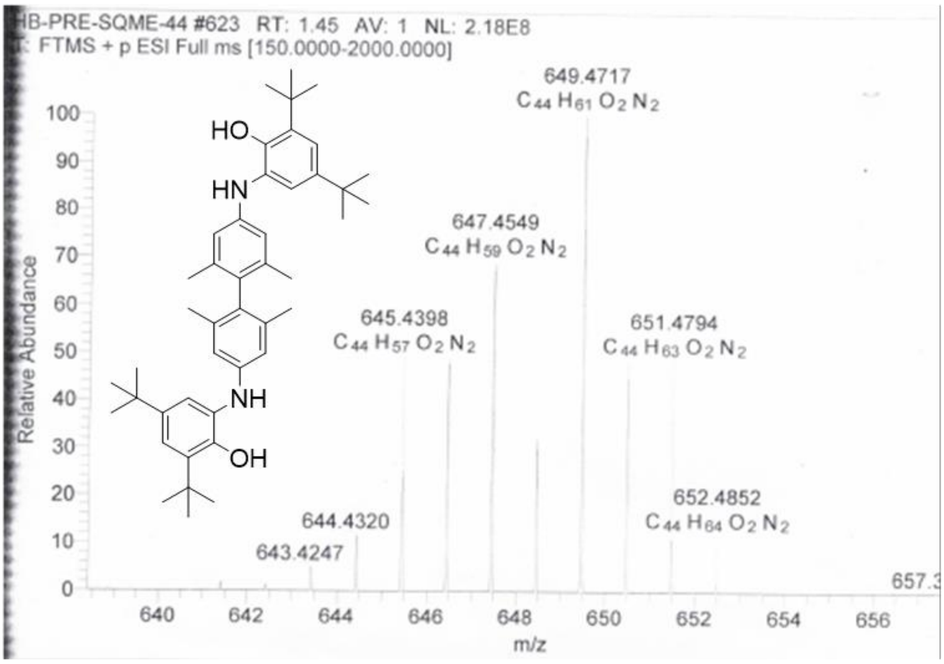

Supplementary Fig. 48: The mass spectrum of compound **4d**.

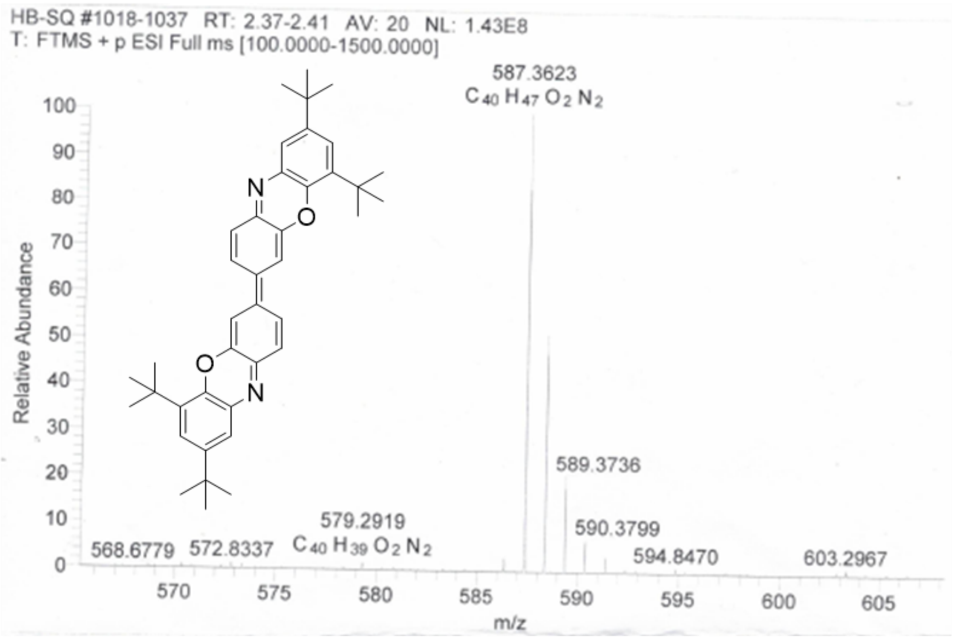

Supplementary Fig. 49: The mass spectrum of compound **1a**.

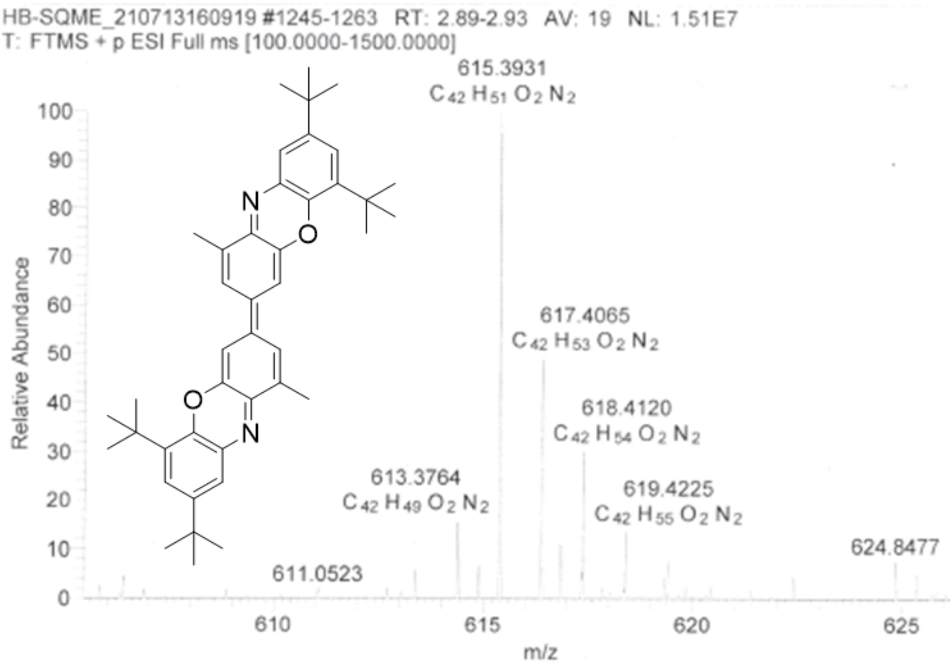

Supplementary Fig. 50: The mass spectrum of compound **1b**.

8. Supplementary references

1. Bain, G. A. & Berry, J. F. Diamagnetic Corrections and Pascal's Constants. *J. Chem. Educ*. **85**, 532-536 (2008).
2. Frisch, M. J. et al. *Gaussian 16, Revision A.03,* Gaussian, Inc., Wallingford, CT, 2016.
3. Chai, J.-D. & Head-Gordon, M. Long-range corrected hybrid density functionals with damped atom-atom dispersion corrections. *Phys. Chem. Chem.* Phys. **10**, 6615-6620 (2008).
4. Hehre, W. J., Ditchfield, R. & J. A. Pople. Self-Consistent Molecular Orbital Methods. XII. Further Extensions of Gaussian-Type Basis Sets for Use in Molecular Orbital Studies of Organic Molecules. *J. Chem. Phys*. **56**, 2257 (1972).
Hariharan, P. C. & Pople, J. A. The influence of polarization functions on molecular orbital hydrogenation energies. *Theoretica chimica acta* **28**, 213-222 (1973).
5. Seeger, R. & Pople, J. A. Self-Consistent Molecular Orbital Methods. 28. Constraints and Stability in Hartree-Fock Theory. *J. Chem. Phys*. **66**, 3045-3050 (1977).
6. Bauernschmitt, R. & Ahlrichs, R. Stability analysis for solutions of the closed shell Kohn-Sham equation. *J. Chem. Phys*. **104**, 9047-9052 (1996).
7. Schlegel, H. B. & McDouall, J. J. in Computational Advances in Organic Chemistry, Ed. Ögretir, C. & Csizmadia, I. G. (Kluwer Academic, The Netherlands) 167-185 (1991).
8. Weigend, F. & Ahlrichs, R. Balanced basis sets of split valence, triple zeta valence and quadruple zeta valence quality for H to Rn: Design and assessment of accuracy. *Phys. Chem. Chem. Phys*. **7**, 3297-3305 (2005).
9. Chen, Z. et al. Nucleus-Independent Chemical Shifts (NICS) as an Aromaticity Criterion. *Chem. Rev*. **105**, 3842-3888 (2015).
10. Fallah-Bagher-Shaidaei, H. et al. Which NICS Aromaticity Index for Planar π Rings Is Best? *Org. Lett*. **8**, 863-866 (2006).
11. Humphrey, W., Dalke, A. & Schulten, K. VMD: Visual molecular dynamics. *J. Mol. Graph*. **14**, 33-38 (1996).
12. Yamaguchi, K. The Electronic Structures of Biradicals in the Unrestricted Hartree-Fock Approximation. *Chem. Phys. Lett*. **33**, 330-335 (1975).
