# Supplementary material for: Design of an open-shell nitrogen-centered diradicaloid with tunable stimuli-responsive electronic properties
Source: Commun Chem. 2022 Oct 14;5:127. doi: 10.1038/s42004-022-00747-8 (PMC9814612; doi:10.1038/s42004-022-00747-8)
Supplement: Supplementary file 3 — Description of Additional Supplementary Files [file 42004_2022_747_MOESM3_ESM.docx]

Description of Additional Supplementary Files

**File name:** Supplementary Data 1

**Description:** Optimized Cartesian coordinates (in Å) of all calculated moleculars in this work at the ωB97XD/6-31G(d,p) level of theory in the gas phase.

**File name:** Supplementary Data 2

**Description:** X-ray of **1a**.

**File name:** Supplementary Data 3

**Description:** X-ray of **1a^2+^**.

**File name:** Supplementary Data 4

**Description:** X-ray of **1b**.

**File name:** Supplementary Data 5

**Description:** X-ray of **4a**.

**File name:** Supplementary Data 6

**Description:** X-ray of **4b**.

**File name:** Supplementary Data 7

**Description:** X-ray of **4d**.
