# Supplementary material for: Design of an open-shell nitrogen-centered diradicaloid with tunable stimuli-responsive electronic properties
Source: Commun Chem. 2022 Oct 14;5:127. doi: 10.1038/s42004-022-00747-8 (PMC9814612; doi:10.1038/s42004-022-00747-8)
Supplement: Supplementary file 4 — Supplementary Data 1 [file 42004_2022_747_MOESM4_ESM.docx]

Cartesian coordinates

Optimized Cartesian coordinates (in Å) of all calculated moleculars at the ωB97XD/6-31G(d,p) level of theory in the gas phase.

**1a**-BS

atom x y z

C 5.36116100 -0.27663900 0.01205600

C 6.42274600 -1.12674200 0.35938200

C 7.69110100 -0.55465300 0.34299200

H 8.53545000 -1.17926500 0.60469600

C 7.94896200 0.78972600 0.00613800

C 6.87384900 1.58643100 -0.32775400

H 6.97903000 2.62950400 -0.59921900

C 5.55978200 1.07631100 -0.33339100

C 3.30025000 1.38047400 -0.66197600

C 2.17294400 2.16937200 -0.99294600

H 2.35214800 3.20404700 -1.26349200

C 0.90527400 1.63621100 -0.98451200

H 0.06519200 2.25830600 -1.27451900

C 0.67980900 0.28142700 -0.64881300

C 1.78411700 -0.51494500 -0.32031900

H 1.66448900 -1.55068800 -0.02169400

C 3.05598600 0.02605000 -0.32921400

C 9.39422600 1.30088800 0.02530300

C 9.97374700 1.14081600 1.44361200

H 11.00535200 1.50734000 1.47491400

H 9.98303300 0.09517700 1.76504300

H 9.38549000 1.71048700 2.16947900

C 10.23806000 0.48376300 -0.97138300

H 11.27333100 0.84116200 -0.97340800

H 9.84032800 0.57986600 -1.98628300

H 10.25447100 -0.57987800 -0.71610700

C 9.48513300 2.78122700 -0.36755200

H 8.92342500 3.41805600 0.32297100

H 9.10863700 2.95450000 -1.38052100

H 10.53024100 3.10453400 -0.34183300

C 6.19636200 -2.60053800 0.73513000

C 5.29287400 -2.69310000 1.98131700

H 5.75123600 -2.17103800 2.82713800

H 5.15729400 -3.74255400 2.26389800

H 4.30803300 -2.25977100 1.80353100

C 5.55319500 -3.35345900 -0.44703900

H 4.57646900 -2.94402900 -0.70738100

H 5.42195000 -4.40916000 -0.18653400

H 6.19596600 -3.30016300 -1.33140500

C 7.51760000 -3.31022200 1.06958600

H 8.20680700 -3.31613200 0.21905100

H 7.30728200 -4.35187600 1.32950700

H 8.02329800 -2.85334300 1.92629100

N 4.53867500 1.92049200 -0.67298000

O 4.09130900 -0.78776300 0.00963900

C -5.36114300 0.27690900 0.01172100

C -6.42274700 1.12706200 0.35885700

C -7.69111100 0.55499500 0.34238700

H -8.53547800 1.17964600 0.60393900

C -7.94896000 -0.78941300 0.00563600

C -6.87382600 -1.58616600 -0.32807700

H -6.97899700 -2.62926100 -0.59946200

C -5.55975100 -1.07606600 -0.33363200

C -3.30019800 -1.38027200 -0.66203300

C -2.17287600 -2.16919800 -0.99288700

H -2.35206900 -3.20389200 -1.26336600

C -0.90520600 -1.63603800 -0.98443100

H -0.06511100 -2.25815700 -1.27435200

C -0.67975400 -0.28123000 -0.64882200

C -1.78407700 0.51516800 -0.32043900

H -1.66446000 1.55093200 -0.02188100

C -3.05594600 -0.02582400 -0.32935600

C -9.39423400 -1.30054800 0.02469900

C -10.23795500 -0.48348500 -0.97213400

H -11.27323300 -0.84086200 -0.97423100

H -10.25436800 0.58017700 -0.71694300

H -9.84012600 -0.57967600 -1.98698800

C -9.97389300 -1.14035500 1.44293800

H -11.00550600 -1.50686100 1.47416700

H -9.38571700 -1.70997800 2.16890800

H -9.98319600 -0.09469000 1.76428600

C -9.48513000 -2.78091600 -0.36804900

H -9.10854900 -2.95427400 -1.38097200

H -8.92349200 -3.41770000 0.32257200

H -10.53024500 -3.10420500 -0.34239700

C -6.19635700 2.60088800 0.73448400

C -5.55287600 3.35362200 -0.44763300

H -6.19546300 3.30028100 -1.33213000

H -5.42159500 4.40934100 -0.18721900

H -4.57613000 2.94407500 -0.70772100

C -5.29312700 2.69353000 1.98085100

H -4.30828100 2.26010600 1.80332400

H -5.15752700 3.74300700 2.26333800

H -5.75170800 2.17160100 2.82663600

C -7.51761300 3.31071100 1.06857300

H -8.02352900 2.85397100 1.92522500

H -7.30727200 4.35237800 1.32841800

H -8.20663700 3.31657400 0.21789000

N -4.53862400 -1.92028700 -0.67306600

O -4.09128300 0.78801300 0.00939900

**1a**-T

C 5.33353700 -0.28439800 0.01871600

C 6.37431700 -1.12406900 0.44629800

C 7.64873900 -0.56645700 0.43838400

H 8.47764400 -1.18309600 0.76104000

C 7.93282600 0.75565300 0.03523800

C 6.87976600 1.54301400 -0.37641500

H 7.00549000 2.56808800 -0.70210900

C 5.55753000 1.04770200 -0.39627000

C 3.30981600 1.34847400 -0.81185200

C 2.20670900 2.12571500 -1.22307200

H 2.40482100 3.14247100 -1.54391100

C 0.93043900 1.60363300 -1.22588400

H 0.10132800 2.21278300 -1.57087300

C 0.68716400 0.27769400 -0.81660200

C 1.76445100 -0.50832400 -0.40741700

H 1.62002300 -1.52589100 -0.06004000

C 3.04523900 0.02275600 -0.40924700

C 9.38302500 1.25134800 0.07274900

C 9.91483200 1.16474100 1.51597100

H 10.94936100 1.52149800 1.56032600

H 9.90097300 0.13874100 1.89529500

H 9.31084200 1.78043500 2.18942300

C 10.24759000 0.37025600 -0.84887100

H 11.28685000 0.71563600 -0.83701000

H 9.88379700 0.41372200 -1.87998800

H 10.24236100 -0.67752400 -0.53440700

C 9.50527400 2.70615300 -0.39885600

H 8.93042100 3.38686300 0.23677600

H 9.16286800 2.82641900 -1.43140200

H 10.55314700 3.01870800 -0.35775600

C 6.11962000 -2.57242900 0.89590300

C 5.17387500 -2.58785200 2.11379000

H 5.60811600 -2.02201200 2.94403500

H 5.01968400 -3.61869400 2.45017000

H 4.19939100 -2.15863300 1.87844100

C 5.50901900 -3.38466400 -0.26397200

H 4.54600400 -2.98225000 -0.58007900

H 5.35780700 -4.42297100 0.05006800

H 6.18184700 -3.38637400 -1.12731400

C 7.42125100 -3.27385300 1.31428500

H 8.13855700 -3.33368700 0.48945500

H 7.19109900 -4.29739200 1.62445600

H 7.90277700 -2.77418300 2.16097700

N 4.56302300 1.87980900 -0.81377700

O 4.05939000 -0.78196900 0.00600600

C -5.33354100 0.28441000 0.01862600

C -6.37432700 1.12409200 0.44617300

C -7.64874300 0.56646500 0.43830000

H -8.47765000 1.18310900 0.76093600

C -7.93282000 -0.75566500 0.03521100

C -6.87975600 -1.54303200 -0.37641800

H -7.00547200 -2.56812300 -0.70206500

C -5.55752500 -1.04771000 -0.39630000

C -3.30980500 -1.34849200 -0.81184700

C -2.20669100 -2.12574900 -1.22301800

H -2.40479700 -3.14251900 -1.54381600

C -0.93042100 -1.60366600 -1.22583300

H -0.10130500 -2.21282900 -1.57078200

C -0.68715500 -0.27770900 -0.81660200

C -1.76444900 0.50832400 -0.40746600

H -1.62002700 1.52590600 -0.06013000

C -3.04523600 -0.02275700 -0.40929400

C -9.38301400 -1.25137200 0.07275200

C -10.24759000 -0.37033100 -0.84890600

H -11.28684700 -0.71572000 -0.83702700

H -10.24236900 0.67746400 -0.53449100

H -9.88380000 -0.41384100 -1.88002300

C -9.91481700 -1.16470400 1.51597100

H -10.94934300 -1.52146900 1.56034600

H -9.31081900 -1.78036000 2.18945000

H -9.90096900 -0.13868600 1.89524800

C -9.50525000 -2.70620100 -0.39878400

H -9.16284800 -2.82651100 -1.43132700

H -8.93038500 -3.38687400 0.23687600

H -10.55311800 -3.01876500 -0.35766300

C -6.11964300 2.57247200 0.89572100

C -5.50931000 3.38474900 -0.26426700

H -6.18228900 3.38639700 -1.12749100

H -5.35813000 4.42307000 0.04973900

H -4.54631700 2.98241500 -0.58054000

C -5.17367600 2.58795900 2.11343300

H -4.19921100 2.15879100 1.87790800

H -5.01947900 3.61881300 2.44977600

H -5.60773200 2.02210300 2.94376400

C -7.42124400 3.27381100 1.31433600

H -7.90257300 2.77412500 2.16112900

H -7.19110700 4.29737400 1.62444200

H -8.13871100 3.33357600 0.48964100

N -4.56301100 -1.87983100 -0.81376400

O -4.05939500 0.78198500 0.00590800

**1a**-CS

C 5.41200900 -0.29342400 -0.00014700

C 6.51181200 -1.16388800 -0.00000800

C 7.76640500 -0.55769000 0.00006600

H 8.64012500 -1.19688200 0.00016700

C 7.97177200 0.83117000 0.00001300

C 6.85139100 1.64523600 -0.00010900

H 6.91504700 2.72648800 -0.00014000

C 5.56453600 1.09746100 -0.00018700

C 3.29534000 1.42737000 -0.00026800

C 2.10491400 2.24847500 -0.00030800

H 2.25454300 3.32226000 -0.00038500

C 0.87128500 1.70234500 -0.00024200

H 0.02093300 2.37207500 -0.00028300

C 0.64372900 0.26749800 -0.00014200

C 1.83144000 -0.55423300 -0.00014600

H 1.77131700 -1.63463900 -0.00010600

C 3.06787200 -0.00830400 -0.00020700

C 9.40323900 1.38084600 0.00007200

C 10.14145800 0.88571900 1.25816700

H 11.16506700 1.27507800 1.27562200

H 10.20049300 -0.20614000 1.29212300

H 9.63086100 1.22294900 2.16538200

C 10.14154900 0.88567100 -1.25795400

H 11.16516500 1.27501300 -1.27534800

H 9.63102000 1.22287700 -2.16521600

H 10.20056200 -0.20619100 -1.29186500

C 9.42875400 2.91499400 0.00004900

H 8.93908700 3.32773400 0.88767400

H 8.93920100 3.32770900 -0.88764900

H 10.46551200 3.26517500 0.00010700

C 6.34219200 -2.69190400 0.00005600

C 5.58034500 -3.13677600 1.26484400

H 6.12337900 -2.83473100 2.16593800

H 5.48536400 -4.22791400 1.27611800

H 4.57864000 -2.70765800 1.30785000

C 5.58079900 -3.13692300 -1.26495200

H 4.57910400 -2.70782100 -1.30836800

H 5.48583100 -4.22806300 -1.27614400

H 6.12415100 -2.83497000 -2.16588500

C 7.69840600 -3.41407300 0.00034600

H 8.29072900 -3.17475600 -0.88865300

H 7.52740900 -4.49466200 0.00037300

H 8.29039900 -3.17465500 0.88953600

N 4.47581800 1.96634400 -0.00028300

O 4.15013900 -0.83218700 -0.00027200

C -5.41202500 0.29341400 0.00013300

C -6.51182000 1.16389000 0.00013100

C -7.76641900 0.55770400 0.00005200

H -8.64013500 1.19689800 0.00004900

C -7.97179200 -0.83115700 -0.00002500

C -6.85141800 -1.64523100 -0.00001900

H -6.91508600 -2.72648300 -0.00008000

C -5.56455800 -1.09746800 0.00006000

C -3.29536500 -1.42738400 0.00006000

C -2.10494000 -2.24849100 0.00001700

H -2.25457200 -3.32227600 0.00001300

C -0.87131000 -1.70236600 -0.00003500

H -0.02095900 -2.37209900 -0.00008000

C -0.64374900 -0.26752000 -0.00005300

C -1.83145800 0.55421400 0.00002400

H -1.77133400 1.63462100 0.00006600

C -3.06789100 0.00828900 0.00009300

C -9.40326300 -1.38082400 -0.00008500

C -10.14152700 -0.88555800 -1.25809900

H -11.16514100 -1.27490500 -1.27555400

H -10.20055300 0.20630500 -1.29193800

H -9.63096900 -1.22269700 -2.16537000

C -10.14151900 -0.88577600 1.25802200

H -11.16513800 -1.27511100 1.27541400

H -9.63095800 -1.22308100 2.16522900

H -10.20052200 0.20608400 1.29204900

C -9.42879100 -2.91497300 -0.00022400

H -8.93915700 -3.32762200 -0.88790800

H -8.93921400 -3.32778600 0.88741500

H -10.46555300 -3.26514300 -0.00028500

C -6.34214000 2.69190200 0.00021500

C -5.58048900 3.13687100 -1.26465500

H -6.12370500 2.83495100 -2.16568100

H -5.48545200 4.22800500 -1.27583100

H -4.57881500 2.70770300 -1.30789100

C -5.58049600 3.13672700 1.26513800

H -4.57882700 2.70754300 1.30833600

H -5.48544800 4.22785900 1.27643400

H -6.12372200 2.83471400 2.16612700

C -7.69830600 3.41416500 0.00025000

H -8.29048100 3.17479300 0.88933200

H -7.52724100 4.49474300 0.00030400

H -8.29048000 3.17488100 -0.88885700

N -4.47584300 -1.96635700 0.00005900

O -4.15015500 0.83217800 0.00024100

**1a^2+^**

O 4.05017600 0.75504600 0.01651000

N 4.54094400 -1.82905800 -0.82419700

C 1.74476200 0.53100600 -0.38335900

H 1.59787700 1.54393900 -0.02561400

C 5.30572100 0.29857700 0.02875800

C 0.69216600 -0.26854900 -0.80072300

C 6.11429300 2.57747600 0.94215200

C 3.03104700 -0.00910800 -0.39477900

C 5.53646100 -1.05619600 -0.41680700

C 6.34558500 1.13423900 0.46565700

C 2.19631600 -2.12720700 -1.24697700

H 2.40043000 -3.13666400 -1.58495800

C 6.86371500 -1.57542100 -0.41823500

H 6.98618000 -2.59496900 -0.76085500

C 0.93216100 -1.60384400 -1.23327700

H 0.10073600 -2.20343100 -1.58644600

C 7.59751500 0.54149600 0.42809200

H 8.43618400 1.14353900 0.75480800

C 7.90174100 -0.79277100 -0.00049500

C 3.29038600 -1.33157700 -0.81962700

C 5.50626700 3.41179500 -0.20390800

H 6.16160700 3.41206700 -1.07966800

H 5.38948700 4.44755200 0.12595700

H 4.52157900 3.05098500 -0.50886800

C 5.17904800 2.57615400 2.16874300

H 4.18375800 2.19061900 1.93749600

H 5.05870600 3.60017800 2.53225300

H 5.60094100 1.98018200 2.98317200

C 9.35613500 -1.25829800 0.03431400

C 9.49575800 -2.70490500 -0.45498400

H 8.93690700 -3.40505800 0.17414400

H 10.54665400 -3.00021600 -0.41439200

H 9.16601600 -2.81925900 -1.49251200

C 10.19510300 -0.34101600 -0.87853900

H 9.83962500 -0.37889000 -1.91231600

H 11.23705500 -0.67143000 -0.86717700

H 10.18396800 0.70260800 -0.54944400

C 9.87250800 -1.17157200 1.48496000

H 9.85242400 -0.15024500 1.87745800

H 10.91106900 -1.51063000 1.52210700

H 9.28492200 -1.80784500 2.15310700

C 7.43671300 3.23963400 1.35756900

H 7.91547800 2.72005500 2.19359400

H 7.23150600 4.26018200 1.68858300

H 8.14526200 3.30728600 0.52601800

O -4.05017800 -0.75503300 0.01649900

N -4.54095500 1.82909000 -0.82414600

C -1.74476600 -0.53097900 -0.38337100

H -1.59788000 -1.54392100 -0.02565300

C -5.30572300 -0.29856900 0.02876000

C -0.69217200 0.26859000 -0.80071400

C -6.11426800 -2.57749000 0.94211000

C -3.03105200 0.00913400 -0.39477300

C -5.53647000 1.05621200 -0.41677800

C -6.34558200 -1.13424700 0.46564200

C -2.19632700 2.12725800 -1.24691100

H -2.40044200 3.13672500 -1.58486000

C -6.86372800 1.57542500 -0.41820600

H -6.98620100 2.59497900 -0.76080500

C -0.93217000 1.60389700 -1.23322500

H -0.10074700 2.20349800 -1.58637400

C -7.59751800 -0.54151600 0.42807400

H -8.43618400 -1.14356800 0.75477600

C -7.90175100 0.79275600 -0.00049600

C -3.29039500 1.33161300 -0.81958500

C -5.17909300 -2.57617700 2.16875500

H -5.60104800 -1.98023800 2.98317600

H -5.05874700 -3.60020800 2.53224300

H -4.18380000 -2.19061100 1.93757200

C -5.50613800 -3.41174200 -0.20394300

H -4.52145400 -3.05086500 -0.50884100

H -5.38931700 -4.44750200 0.12589700

H -6.16143100 -3.41202100 -1.07973700

C -9.35615000 1.25826700 0.03430300

C -9.49578100 2.70488800 -0.45495200

H -9.16601900 2.81927900 -1.49247000

H -10.54668100 3.00018500 -0.41437100

H -8.93695000 3.40502800 0.17421000

C -9.87255000 1.17148900 1.48493600

H -9.28498500 1.80775000 2.15311400

H -10.91111700 1.51053100 1.52207300

H -9.85245900 0.15015000 1.87740300

C -10.19509100 0.34100500 -0.87859500

H -10.18395400 -0.70262800 -0.54953100

H -11.23704700 0.67140900 -0.86724500

H -9.83959200 0.37891400 -1.91236400

C -7.43668400 -3.23971800 1.35742900

H -8.14517600 -3.30739300 0.52583100

H -7.23145200 -4.26026300 1.68844000

H -7.91552900 -2.72017400 2.19343100

**1a•**(TFA)_2_-BS

C -5.06226900 -1.77237100 -0.47755500

C -5.83240800 -2.88885000 -0.83853500

C -7.21021800 -2.70208200 -0.82562200

H -7.83936400 -3.53992100 -1.09707000

C -7.84611200 -1.49064800 -0.48034500

C -7.05315900 -0.41845800 -0.13176200

H -7.46682000 0.54440400 0.14750400

C -5.64892200 -0.53885300 -0.12366200

C -3.54699100 0.39767600 0.22567500

C -2.69448200 1.47104800 0.57282400

H -3.14517000 2.41641700 0.85467800

C -1.32850300 1.31142100 0.56560400

H -0.69803200 2.14109800 0.86646200

C -0.73251700 0.07858600 0.21652400

C -1.56382300 -0.99145000 -0.12604300

H -1.15842900 -1.94907000 -0.43346000

C -2.93821400 -0.82950600 -0.11898100

C -9.37627400 -1.41396400 -0.50399200

C -9.87899200 -1.72816700 -1.92593900

H -10.97181800 -1.66971500 -1.95956400

H -9.59078100 -2.73234200 -2.25170400

H -9.47660900 -1.01039400 -2.64719700

C -9.95105000 -2.44511300 0.48583000

H -11.04496400 -2.39843400 0.48434100

H -9.60226400 -2.24267200 1.50301400

H -9.66243000 -3.46845800 0.22693700

C -9.88491100 -0.02246600 -0.10590800

H -9.52497300 0.75475900 -0.78601000

H -9.57550000 0.25320600 0.90615500

H -10.97868000 -0.01205700 -0.13560800

C -5.19117200 -4.23252900 -1.22274700

C -4.29274600 -4.05228000 -2.46301900

H -4.87558000 -3.67275300 -3.30806400

H -3.86504900 -5.01764900 -2.75312100

H -3.47017300 -3.36104800 -2.27540700

C -4.36681500 -4.78090600 -0.04043300

H -3.54575700 -4.11549800 0.22922600

H -3.94128900 -5.75412100 -0.30652100

H -5.00198700 -4.91864700 0.84014500

C -6.25413000 -5.28573900 -1.57184800

H -6.91733200 -5.49558800 -0.72664400

H -5.75432900 -6.22182200 -1.83707400

H -6.86417300 -4.98479900 -2.42946700

N -4.89497800 0.54688400 0.22709800

O -3.70040600 -1.89534300 -0.47031700

C 5.06225300 1.77239300 -0.47747200

C 5.83242500 2.88878700 -0.83863500

C 7.21023700 2.70204500 -0.82547600

H 7.83941100 3.53981300 -1.09708000

C 7.84610100 1.49070100 -0.47983500

C 7.05310600 0.41856200 -0.13118300

H 7.46673600 -0.54434200 0.14796100

C 5.64887000 0.53895900 -0.12323500

C 3.54690900 -0.39747400 0.22617300

C 2.69436500 -1.47075700 0.57351800

H 3.14503400 -2.41604700 0.85566900

C 1.32838700 -1.31114200 0.56610100

H 0.69788400 -2.14074200 0.86710500

C 0.73243400 -0.07840100 0.21662400

C 1.56377300 0.99154600 -0.12613700

H 1.15840700 1.94908400 -0.43384700

C 2.93816400 0.82961600 -0.11887500

C 9.37626200 1.41398800 -0.50341300

C 9.95115600 2.44613200 0.48529800

H 11.04506100 2.39927700 0.48386900

H 9.66274300 3.46925700 0.22531900

H 9.60233500 2.24484800 1.50270000

C 9.87885200 1.72672400 -1.92573300

H 10.97168500 1.66840400 -1.95939200

H 9.47650700 1.00811900 -2.64618100

H 9.59041900 2.73050600 -2.25252000

C 9.88489800 0.02291100 -0.10386600

H 9.57538600 -0.25160700 0.90849200

H 9.52506900 -0.75509700 -0.78311500

H 10.97867100 0.01251100 -0.13342700

C 5.19122100 4.23236200 -1.22325600

C 4.36676200 4.78101900 -0.04114400

H 5.00186100 4.91899300 0.83945000

H 3.94123000 5.75415600 -0.30750900

H 3.54569900 4.11565500 0.22861700

C 4.29288900 4.05180200 -2.46355100

H 3.47029500 3.36062900 -2.27582500

H 3.86522600 5.01710100 -2.75393400

H 4.87578400 3.67205100 -3.30845400

C 6.25419800 5.28549200 -1.57254000

H 6.86430000 4.98434400 -2.43004500

H 5.75441100 6.22150900 -1.83802800

H 6.91734200 5.49555000 -0.72734300

N 4.89489100 -0.54667700 0.22778800

O 3.70038800 1.89537600 -0.47036700

F -8.27635400 5.15823100 1.55817000

C -6.99807400 4.80947200 1.45915400

C -6.86285600 3.33495800 1.01970700

F -6.40463600 5.61689400 0.57030500

F -6.41250400 4.99793100 2.64873500

O -5.60363000 2.99616200 0.93282400

O -7.83407400 2.65023700 0.80259500

H -5.47384700 2.02384200 0.64772700

F 8.27644800 -5.15979200 1.55245300

C 6.99817600 -4.80950800 1.45889400

C 6.86284000 -3.33528700 1.01849700

F 6.39962400 -5.61722900 0.57376600

F 6.41783900 -4.99577500 2.65139600

O 5.60363300 -2.99549800 0.93514100

O 7.83396400 -2.65150500 0.79803500

H 5.47386000 -2.02342600 0.64923900

**1a•**(TFA)_2_-T

C -5.04343900 1.75133700 0.54764600

C -5.80234600 2.85056500 0.97973100

C -7.18070800 2.67167800 0.97678200

H -7.80117400 3.49621500 1.30313500

C -7.82894500 1.48274100 0.57524700

C -7.04869100 0.42724600 0.15827600

H -7.47181000 -0.51741300 -0.16541700

C -5.64193300 0.53906000 0.13580200

C -3.54602400 -0.38343600 -0.29047800

C -2.70630800 -1.43812100 -0.70538100

H -3.16482100 -2.36617700 -1.02923200

C -1.33674400 -1.28425900 -0.70828800

H -0.70928100 -2.09841500 -1.05518700

C -0.73706800 -0.07663700 -0.29860100

C -1.55290900 0.97548500 0.11168100

H -1.13481300 1.91421700 0.45881900

C -2.93133600 0.81798500 0.11211200

C -9.35898200 1.41372200 0.61766200

C -9.83826500 1.65221400 2.06227900

H -10.93084500 1.59879000 2.10908200

H -9.53827600 2.63505600 2.43854700

H -9.43040000 0.89316100 2.73667500

C -9.94138000 2.50112000 -0.30532200

H -11.03540600 2.46028200 -0.29010600

H -9.60875500 2.35300600 -1.33717500

H -9.64284100 3.50716600 0.00502000

C -9.88283200 0.04931300 0.15145700

H -9.51803500 -0.76649400 0.78188900

H -9.59070400 -0.17159300 -0.87900000

H -10.97607300 0.04386100 0.19738000

C -5.14743200 4.16766300 1.42765300

C -4.23106600 3.91439400 2.64188000

H -4.80294500 3.49076800 3.47335000

H -3.79408300 4.86021600 2.97870400

H -3.41493400 3.23120200 2.40330800

C -4.33820700 4.77632500 0.26455200

H -3.52633300 4.12192300 -0.05522200

H -3.90157900 5.73041800 0.57765500

H -4.98637800 4.96710500 -0.59643000

C -6.19886800 5.20509700 1.85096500

H -6.87363000 5.46459200 1.02900200

H -5.68972200 6.12258100 2.15975800

H -6.79755000 4.86005500 2.69996200

N -4.90271300 -0.52741100 -0.28176000

O -3.68238200 1.86821700 0.52842000

C 5.04357000 -1.75148600 0.54652600

C 5.80257100 -2.85075200 0.97835500

C 7.18092700 -2.67183500 0.97522900

H 7.80146300 -3.49639900 1.30138400

C 7.82908000 -1.48283100 0.57375100

C 7.04873800 -0.42730300 0.15703200

H 7.47178200 0.51741700 -0.16657200

C 5.64197800 -0.53914800 0.13474700

C 3.54599600 0.38338000 -0.29107700

C 2.70620300 1.43811600 -0.70569100

H 3.16465400 2.36621600 -1.02950700

C 1.33663800 1.28425100 -0.70837000

H 0.70911300 2.09844800 -1.05506100

C 0.73703900 0.07657800 -0.29872200

C 1.55295700 -0.97559400 0.11127800

H 1.13492800 -1.91437400 0.45836800

C 2.93138500 -0.81809400 0.11147300

C 9.35912500 -1.41378100 0.61596900

C 9.94143500 -2.50110400 -0.30715100

H 11.03546200 -2.46026400 -0.29203300

H 9.64293400 -3.50717700 0.00314100

H 9.60871400 -2.35291500 -1.33896300

C 9.83857300 -1.65237800 2.06051300

H 10.93115800 -1.59895200 2.10720400

H 9.43077300 -0.89338000 2.73500900

H 9.53861900 -2.63525100 2.43673000

C 9.88291500 -0.04933600 0.14978900

H 9.59061900 0.17164700 -0.88060600

H 9.51823100 0.76643500 0.78032900

H 10.97616300 -0.04389900 0.19553400

C 5.14776200 -4.16791200 1.42623500

C 4.33861000 -4.77663400 0.26311200

H 4.98681200 -4.96734400 -0.59786200

H 3.90206800 -5.73077200 0.57619700

H 3.52667700 -4.12231000 -0.05666800

C 4.23135900 -3.91473300 2.64045200

H 3.41518800 -3.23158600 2.40188100

H 3.79443100 -4.86059200 2.97724400

H 4.80319600 -3.49109000 3.47194300

C 6.19929800 -5.20524500 1.84954100

H 6.79798300 -4.86012100 2.69850300

H 5.69024100 -6.12276200 2.15838200

H 6.87405200 -5.46470800 1.02756200

N 4.90268700 0.52735800 -0.28258900

O 3.68251000 -1.86838600 0.52749200

F -8.32064000 -5.05763600 -1.77356300

C -7.03984500 -4.71361000 -1.69093900

C -6.89155000 -3.26372100 -1.17957100

F -6.42345100 -5.56597600 -0.86166500

F -6.48670900 -4.83961400 -2.90412400

O -5.63024800 -2.93132100 -1.10343400

O -7.85657400 -2.58988300 -0.90704000

H -5.49113800 -1.97489500 -0.77014000

F 8.32016100 5.05815600 -1.77387600

C 7.03940400 4.71398200 -1.69129200

C 6.89125000 3.26408400 -1.17990300

F 6.42289700 5.56625700 -0.86200500

F 6.48627700 4.83995400 -2.90448100

O 5.62999700 2.93132400 -1.10445900

O 7.85632500 2.59042300 -0.90711600

H 5.49099200 1.97489500 -0.77119100

**1a•**(TPFB)_2_-BS

C 4.47925400 -2.69626000 -0.10446700

C 4.94634500 -4.01059000 0.07135400

C 6.30485500 -4.19642400 -0.11001600

H 6.70769600 -5.19301600 0.00833500

C 7.19980800 -3.15694500 -0.43822300

C 6.70444900 -1.87689300 -0.54675100

H 7.36740000 -1.06083400 -0.76129500

C 5.33000500 -1.60780100 -0.39881100

C 3.44579300 -0.21935400 -0.64761800

C 2.78237900 0.92053400 -1.15442200

H 3.35922000 1.73892300 -1.54617000

C 1.40971500 0.98496900 -1.19143100

H 0.93962900 1.86964700 -1.60696500

C 0.60674500 -0.08231300 -0.74353800

C 1.23689200 -1.24356900 -0.32201000

H 0.67917000 -2.10537500 0.02736200

C 2.62439800 -1.30863500 -0.30374800

C 8.68477800 -3.48378000 -0.62771000

C 9.27377600 -3.87061400 0.74247100

H 10.33699000 -4.11108600 0.64194100

H 8.77015600 -4.74490600 1.16674400

H 9.17444300 -3.04498100 1.45360100

C 8.84011500 -4.65900300 -1.61208500

H 9.90226100 -4.87126200 -1.76763500

H 8.39657300 -4.41703400 -2.58280300

H 8.37590300 -5.57920200 -1.24649900

C 9.47276200 -2.29286700 -1.18753300

H 9.48612900 -1.44488600 -0.50075400

H 9.06304700 -1.95660900 -2.14607900

H 10.51123300 -2.58994400 -1.35810900

C 4.00224300 -5.16321700 0.45306800

C 3.35504400 -4.87954700 1.82442400

H 4.12319000 -4.74407200 2.59167500

H 2.72870600 -5.72822700 2.11690700

H 2.72769200 -3.98788200 1.81012700

C 2.91888700 -5.34902700 -0.62880900

H 2.27930800 -4.47116600 -0.72851600

H 2.28200300 -6.19983000 -0.36719800

H 3.37558600 -5.55550400 -1.60179700

C 4.76660200 -6.49140700 0.56997400

H 5.23874200 -6.77959300 -0.37480500

H 4.06239600 -7.28327400 0.83935200

H 5.53307900 -6.45654700 1.35051900

N 4.81870800 -0.31682500 -0.46691100

O 3.16005400 -2.47712300 0.09877700

C -4.72603900 2.67971000 -0.22477200

C -5.18850800 4.00476300 -0.14122200

C -6.54945400 4.17956300 -0.31431700

H -6.94949600 5.18311500 -0.26581800

C -7.45079300 3.12033100 -0.54826300

C -6.95885500 1.83453700 -0.56903900

H -7.62730600 1.00682100 -0.70910100

C -5.58214600 1.57410300 -0.42486200

C -3.70245800 0.16794100 -0.60151400

C -3.04702500 -1.00673000 -1.03391700

H -3.63016800 -1.85116300 -1.35430200

C -1.67522500 -1.07529800 -1.08898500

H -1.21219300 -1.98865900 -1.44633100

C -0.86492900 0.02107300 -0.73433000

C -1.48822600 1.21040200 -0.38764800

H -0.92497200 2.09485500 -0.11141200

C -2.87526400 1.27815100 -0.35116500

C -8.93808500 3.43652000 -0.73757900

C -9.10664400 4.53252500 -1.80744500

H -10.17054000 4.73818200 -1.95986700

H -8.63148800 5.47543800 -1.52278800

H -8.68205300 4.21327100 -2.76418800

C -9.50325400 3.93078000 0.60787200

H -10.56749900 4.16626200 0.50646600

H -9.39376900 3.16269800 1.37941500

H -8.99088200 4.83377000 0.95440200

C -9.73803400 2.20875700 -1.19023000

H -9.34460600 1.79690800 -2.12584200

H -9.74245800 1.41708700 -0.43918100

H -10.77831500 2.49562400 -1.36716700

C -4.23659300 5.18073500 0.13565000

C -3.17043300 5.27970800 -0.97441400

H -3.64234300 5.41215600 -1.95302100

H -2.52802500 6.14625300 -0.78910800

H -2.53399600 4.39495000 -1.01654100

C -3.56786600 5.00180500 1.51427000

H -2.94215200 4.11001300 1.55867000

H -2.93560900 5.86873800 1.73057000

H -4.32376900 4.92792100 2.30181600

C -4.99663700 6.51628000 0.16176600

H -5.74999900 6.54470800 0.95522600

H -4.28654200 7.32443200 0.35718800

H -5.48380100 6.73184100 -0.79466500

N -5.07261000 0.28067300 -0.40901500

O -3.40395400 2.47373600 -0.02666600

B 5.79614200 0.97296900 -0.12491000

C 4.90321200 2.32619000 0.16249700

C 3.93986200 2.31389500 1.17429700

C 3.17459300 3.41150000 1.53420400

C 3.38963200 4.62431200 0.89411700

C 4.36231200 4.70377100 -0.08906800

C 5.09989600 3.57232800 -0.42868200

C 6.82520700 1.10704700 -1.41639500

C 6.31425300 1.29352000 -2.69940100

C 7.07982600 1.39236400 -3.85132400

C 8.45997800 1.29440600 -3.75963700

C 9.02949700 1.09725400 -2.51303600

C 8.21706300 1.01591900 -1.38776500

C 6.53510400 0.75244600 1.33789600

C 7.44055500 1.73885900 1.73926400

C 8.08203100 1.76069800 2.96677300

C 7.79756000 0.77134000 3.89816100

C 6.86563100 -0.19959600 3.57657200

C 6.24208000 -0.17711400 2.33212000

F 5.29460500 -1.11633300 2.16869400

F 6.54656400 -1.13934500 4.46742300

F 8.39794800 0.76761400 5.08451400

F 8.95268800 2.72443000 3.26279600

F 7.72813500 2.75125400 0.90717300

F 8.87774800 0.74254300 -0.25018800

F 10.34862900 0.95617200 -2.39586900

F 9.21818400 1.37230500 -4.84763400

F 6.50023500 1.57810300 -5.03608800

F 4.98647700 1.40292400 -2.88433900

F 6.01973700 3.75870500 -1.38116700

F 4.58989100 5.86344200 -0.70283000

F 2.67002800 5.69389100 1.22043900

F 2.24133700 3.31233800 2.48036000

F 3.67554600 1.17163100 1.82957300

B -6.04735400 -0.98122200 0.02996000

C -7.08948200 -1.20207300 -1.23903100

C -6.59242900 -1.47872200 -2.51116700

C -7.37020500 -1.65469800 -3.64561100

C -8.74908100 -1.54655800 -3.54694900

C -9.30516400 -1.26216600 -2.31117300

C -8.48076600 -1.10578400 -1.20266600

C -6.77140600 -0.66123600 1.48184700

C -6.46785500 0.33305400 2.40799800

C -7.07885700 0.43991400 3.65439200

C -8.00784800 -0.50626200 4.05006000

C -8.30206500 -1.55586700 3.19049000

C -7.67323900 -1.61735500 1.95786600

C -5.15336100 -2.31295700 0.40113700

C -5.35967600 -3.59710300 -0.09797400

C -4.62187800 -4.70409000 0.31423000

C -3.63884200 -4.55789600 1.27931200

C -3.41321700 -3.30316800 1.82867200

C -4.17903600 -2.23149700 1.39900000

F -3.90443200 -1.04552600 1.96670800

F -2.46966000 -3.14024100 2.75553600

F -2.91893100 -5.60389800 1.67416500

F -4.85905500 -5.90394000 -0.21245900

F -6.28958500 -3.84705200 -1.02581700

F -7.97009900 -2.68305700 1.19882600

F -9.16992900 -2.49665900 3.55943700

F -8.59655600 -0.42188400 5.23927100

F -6.75108400 1.43753000 4.47655600

F -5.52253400 1.25870400 2.17127200

F -9.12900900 -0.75460900 -0.07942900

F -10.62275800 -1.11007100 -2.19032000

F -9.51870400 -1.69747600 -4.61914400

F -6.80334400 -1.92380000 -4.82050400

F -5.26704700 -1.60577600 -2.70169100

**1a•**(TPFB)_2_-T

C 4.33915900 -2.62703400 -0.05046300

C 4.71756800 -3.95256500 0.22761400

C 6.06999200 -4.22394800 0.13321200

H 6.40788900 -5.23199400 0.33149400

C 7.04046500 -3.25668100 -0.20556200

C 6.62846900 -1.96060300 -0.41730300

H 7.34898500 -1.19843400 -0.64439400

C 5.26593800 -1.60598900 -0.36193900

C 3.47816700 -0.13697500 -0.79816900

C 2.90932300 1.00242300 -1.40753400

H 3.55150800 1.76913400 -1.80227100

C 1.54473700 1.13355900 -1.53572000

H 1.14262900 2.01795300 -2.01831300

C 0.66782900 0.13199500 -1.08423200

C 1.20335600 -1.02794500 -0.54894500

H 0.57349200 -1.82964100 -0.17810900

C 2.58245900 -1.15758300 -0.43688000

C 8.51122300 -3.67696600 -0.29356700

C 9.00262100 -4.01963200 1.12601000

H 10.05335600 -4.32543300 1.09787400

H 8.42614300 -4.83883800 1.56709900

H 8.91556600 -3.15124600 1.78597000

C 8.64778700 -4.91213500 -1.20458700

H 9.70187000 -5.19393400 -1.28767800

H 8.27299400 -4.69918800 -2.21044100

H 8.10897100 -5.78190200 -0.81850300

C 9.39752000 -2.56553600 -0.86951500

H 9.42355300 -1.68256100 -0.22869000

H 9.06123600 -2.26090900 -1.86646400

H 10.42427500 -2.92966100 -0.96424000

C 3.68613600 -5.02322200 0.62287100

C 2.98923400 -4.62076400 1.93920600

H 3.72484300 -4.47996600 2.73685300

H 2.30146600 -5.41471200 2.24682400

H 2.41415400 -3.69989700 1.83914900

C 2.64746600 -5.21023900 -0.50203000

H 2.06979700 -4.30374600 -0.68654600

H 1.94502900 -6.00214800 -0.22400600

H 3.13704500 -5.50443800 -1.43571800

C 4.36134700 -6.38409700 0.85527300

H 4.86381800 -6.75363100 -0.04451300

H 3.59715400 -7.11687000 1.12823700

H 5.08665100 -6.34815200 1.67424800

N 4.83518800 -0.29702100 -0.52966300

O 3.02850700 -2.32326800 0.06918700

C -4.70980400 2.76476000 -0.27099200

C -5.22332500 4.05192100 -0.03273100

C -6.60079700 4.17121400 -0.08516400

H -7.03872800 5.14563100 0.08411600

C -7.47180500 3.09145800 -0.34265600

C -6.92637100 1.84065000 -0.52175600

H -7.56072900 0.99460700 -0.70528500

C -5.53331700 1.64046700 -0.50744400

C -3.61916400 0.36420300 -0.97950400

C -2.96692700 -0.72173000 -1.59796200

H -3.55081500 -1.52802000 -2.00644000

C -1.59437600 -0.76419600 -1.67176300

H -1.11445300 -1.62102800 -2.13202400

C -0.80131500 0.27864100 -1.15866200

C -1.42685600 1.42249500 -0.69002000

H -0.86469800 2.27038800 -0.31408700

C -2.81636900 1.46408800 -0.63341700

C -8.98210000 3.34365300 -0.39062900

C -9.28652200 4.48375500 -1.38130600

H -10.36686500 4.65051200 -1.43232800

H -8.82442000 5.43007300 -1.08628100

H -8.93367500 4.23243400 -2.38622700

C -9.45517900 3.73979700 1.02119600

H -10.53359900 3.92750800 1.01799000

H -9.24869600 2.93926400 1.73784400

H -8.95827400 4.64815300 1.37583900

C -9.76199700 2.10202400 -0.84163200

H -9.44363900 1.76713600 -1.83475500

H -9.65366800 1.26941500 -0.14360600

H -10.82730100 2.34155100 -0.90063800

C -4.30645800 5.24482900 0.28572900

C -3.32250800 5.49072600 -0.87595700

H -3.86412700 5.68237400 -1.80754900

H -2.70821000 6.36935500 -0.65565300

H -2.65113900 4.64624700 -1.03574100

C -3.54009600 4.97919300 1.59807400

H -2.87624200 4.11755000 1.52304300

H -2.93170900 5.85329700 1.85090500

H -4.23792000 4.80105900 2.42173000

C -5.11962300 6.53456200 0.47888900

H -5.81571100 6.45934100 1.32016300

H -4.43302800 7.35714200 0.69675400

H -5.68174600 6.80563600 -0.42064100

N -4.96996000 0.38070800 -0.65171500

O -3.37035800 2.60125900 -0.17095600

B 5.86193400 0.95480700 -0.19964800

C 5.02856600 2.36496100 -0.03497100

C 4.00915200 2.45372300 0.91602900

C 3.28142300 3.60468500 1.17160900

C 3.59590900 4.76929300 0.48489500

C 4.62702500 4.74862300 -0.44010100

C 5.32368800 3.56596700 -0.67610800

C 6.96737000 0.96348400 -1.43401000

C 6.54083600 1.10790300 -2.75273700

C 7.37399400 1.09854100 -3.86119500

C 8.73839600 0.92669800 -3.68362900

C 9.22486200 0.76718300 -2.39708300

C 8.34761600 0.79529400 -1.31886200

C 6.50890900 0.77945200 1.31216300

C 7.44493800 1.73892900 1.70864200

C 8.02146900 1.79814700 2.96666800

C 7.63537400 0.87954200 3.93323900

C 6.67021200 -0.05909600 3.61383400

C 6.11443400 -0.07575200 2.33750200

F 5.12773300 -0.97345800 2.17321700

F 6.25511900 -0.92924500 4.53540100

F 8.17208500 0.91264100 5.14940400

F 8.92675500 2.73117700 3.25751100

F 7.83122500 2.68521200 0.83948300

F 8.92657800 0.54987800 -0.13137600

F 10.52466300 0.55778700 -2.19533200

F 9.55917700 0.90036500 -4.72791900

F 6.87347000 1.25087800 -5.08611200

F 5.23469200 1.28282100 -3.02109700

F 6.30559800 3.65465400 -1.57944800

F 4.94894700 5.86136700 -1.09709700

F 2.91455200 5.88850200 0.71221700

F 2.28966600 3.60215000 2.06226300

F 3.64950400 1.36245500 1.61160200

B -5.79414400 -0.96926000 -0.15179800

C -7.00798900 -1.18534400 -1.25468200

C -6.70082100 -1.37164300 -2.60094000

C -7.63603800 -1.52817200 -3.61253700

C -8.98654400 -1.49192600 -3.29906200

C -9.35711200 -1.29798100 -1.97895800

C -8.37948400 -1.15543800 -1.00065800

C -6.29805400 -0.78110700 1.40948200

C -5.90710300 0.17095400 2.34598100

C -6.32136800 0.16294100 3.67505600

C -7.12834000 -0.86105900 4.13873300

C -7.49877500 -1.87140500 3.26147600

C -7.06632600 -1.81771000 1.94681000

C -4.76676500 -2.25233800 -0.02478100

C -4.94809800 -3.50513400 -0.60812500

C -4.04126700 -4.55291200 -0.46794600

C -2.89814400 -4.37883300 0.29568200

C -2.69360800 -3.16371300 0.93264700

C -3.63678600 -2.15842100 0.79177700

F -3.36620500 -1.00916100 1.43168300

F -1.58308000 -2.96381700 1.64718300

F -2.00760000 -5.35956700 0.41568400

F -4.26421500 -5.72016200 -1.06852500

F -6.01440900 -3.77607600 -1.36725400

F -7.42271600 -2.84911400 1.16619100

F -8.25005700 -2.88432700 3.69030800

F -7.52870500 -0.88719100 5.40642000

F -5.92090800 1.12599500 4.50614900

F -5.05709100 1.16487000 2.03431800

F -8.85942400 -0.88272700 0.22405000

F -10.64552500 -1.21181400 -1.65274100

F -9.90548700 -1.62524300 -4.24898700

F -7.24737200 -1.70855400 -4.87351000

F -5.41567900 -1.42121900 -2.99582000

**1d**-BS

C -5.16958900 0.19665500 -0.25468100

C -6.07703800 1.04011300 -0.91625100

C -7.39650700 0.97820800 -0.48011700

H -8.12521900 1.61418100 -0.96603500

C -7.84814200 0.13685000 0.55807100

C -6.92209200 -0.67403800 1.17799500

H -7.18094300 -1.35051800 1.98312300

C -5.56475600 -0.66639000 0.79057600

C -3.40664200 -1.44437300 1.03065000

C -2.43849100 -2.26272700 1.64867700

H -2.77820900 -2.91352300 2.44798200

C -1.11548400 -2.23956400 1.25687100

C -0.71584400 -1.37302200 0.20723900

C -1.64318600 -0.54517400 -0.43377800

C -2.97038700 -0.59912500 -0.00612400

C -9.33114000 0.16080200 0.94590300

C -9.71644800 1.58037400 1.40354900

H -10.77315100 1.61249300 1.68981300

H -9.56416800 2.31967200 0.61170100

H -9.11789300 1.88496000 2.26751000

C -10.18432000 -0.23658600 -0.27362600

H -11.24755600 -0.22959900 -0.01080500

H -9.92310600 -1.24171800 -0.61850000

H -10.04464100 0.45303800 -1.11130000

C -9.64188000 -0.81427800 2.08890100

H -9.08145500 -0.56605200 2.99562000

H -9.41068800 -1.84773200 1.81259900

H -10.70746300 -0.76619700 2.33335100

C -5.63841500 1.97442100 -2.05597400

C -4.58474100 2.97529800 -1.54131700

H -4.99487200 3.57820200 -0.72501200

H -4.29004400 3.65330300 -2.34944800

H -3.68939600 2.46988500 -1.17811900

C -5.06319100 1.14603500 -3.22203400

H -4.18821600 0.57102800 -2.91715500

H -4.76681800 1.81093300 -4.04037000

H -5.81555500 0.44979700 -3.60574500

C -6.81722900 2.79057100 -2.60848000

H -7.59980100 2.14942100 -3.02666600

H -6.45703500 3.43751500 -3.41390100

H -7.26421500 3.43440600 -1.84421500

N -4.70107700 -1.49361100 1.44501400

O -3.85860600 0.21652800 -0.64167900

C 5.16983300 0.19221100 0.25784300

C 6.07770100 1.02746000 0.92918600

C 7.39667300 0.97189200 0.49068500

H 8.12557400 1.60196500 0.98395800

C 7.84748400 0.14424800 -0.55881500

C 6.92105900 -0.65898900 -1.18807000

H 7.17928700 -1.32484200 -2.00220100

C 5.56417400 -0.65696100 -0.79902500

C 3.40596900 -1.43207900 -1.04732700

C 2.43728300 -2.24196200 -1.67557600

H 2.77639400 -2.88218800 -2.48362100

C 1.11445100 -2.22347600 -1.28295000

C 0.71556400 -1.37068800 -0.22184000

C 1.64352900 -0.55187200 0.42980100

C 2.97051700 -0.60056300 0.00083800

C 9.33001100 0.17380300 -0.94806100

C 10.18478600 -0.23887600 0.26525500

H 11.24771100 -0.22801800 0.00130300

H 10.04579600 0.43981400 1.11192300

H 9.92442000 -1.24849400 0.59743000

C 9.71425500 1.59925400 -1.38797800

H 10.77058400 1.63539300 -1.67514400

H 9.11451500 1.91463200 -2.24723200

H 9.56276200 2.32835000 -0.58657600

C 9.63976400 -0.78645900 -2.10381100

H 9.40925500 -1.82344500 -1.84047300

H 9.07820700 -0.52687200 -3.00664200

H 10.70504800 -0.73487400 -2.34885100

C 5.64026900 1.94642200 2.08176900

C 5.06886400 1.10210200 3.23822700

H 5.82316100 0.40221900 3.61136700

H 4.77308500 1.75587300 4.06568800

H 4.19420000 0.52958200 2.92784800

C 4.58402100 2.95245100 1.58266300

H 3.68826400 2.45057200 1.21559600

H 4.29080700 3.61983300 2.40011600

H 4.99120200 3.56613200 0.77293700

C 6.81925000 2.75691200 2.64217900

H 7.26351000 3.41132800 1.88535200

H 6.46010600 3.39271700 3.45688100

H 7.60370400 2.11142500 3.05003900

N 4.70008500 -1.47589500 -1.46327300

O 3.85930400 0.20637500 0.64664600

C -0.09784800 -3.11896000 1.93786300

H 0.42917200 -3.75200300 1.21677100

H 0.66451200 -2.52028800 2.44725400

H -0.57453900 -3.76472500 2.67839400

C 1.27285400 0.37284800 1.55898700

H 0.21598600 0.28370400 1.81193500

H 1.86310400 0.15426400 2.45430100

H 1.47507000 1.41512900 1.29251900

C -1.27182600 0.39381300 -1.55089500

H -0.21323300 0.31300800 -1.79938600

H -1.85618500 0.18160900 -2.45165400

H -1.48129200 1.43198500 -1.27436200

C 0.09622700 -3.09340500 -1.97514500

H -0.43054200 -3.73601000 -1.26237200

H -0.66627700 -2.48777400 -2.47601500

H 0.57237900 -3.72924400 -2.72456000

**1d**-T

C -5.16601900 0.19857700 -0.25372400

C -6.07081400 1.04759100 -0.91183300

C -7.39117000 0.98531500 -0.47846300

H -8.11794900 1.62549900 -0.96172400

C -7.84616100 0.13832600 0.55370300

C -6.92275300 -0.67816000 1.17014100

H -7.18432100 -1.35931900 1.97043300

C -5.56465300 -0.67056000 0.78522700

C -3.40842700 -1.45443700 1.02359700

C -2.44309900 -2.27908600 1.63745500

H -2.78533600 -2.93410000 2.43223000

C -1.11943700 -2.25625800 1.24770300

C -0.71636900 -1.38303200 0.20512500

C -1.64054900 -0.54823400 -0.43110200

C -2.96872800 -0.60271000 -0.00627300

C -9.32980400 0.16259600 0.93902500

C -9.71328200 1.57999200 1.40487900

H -10.77043400 1.61230300 1.68945200

H -9.55819600 2.32393000 0.61793700

H -9.11570700 1.87805200 2.27178900

C -10.18153300 -0.22550700 -0.28450400

H -11.24521900 -0.21826700 -0.02352000

H -9.92151300 -1.22889800 -0.63529800

H -10.03912800 0.46917600 -1.11752700

C -9.64440000 -0.81905500 2.07531800

H -9.08514900 -0.57755100 2.98457300

H -9.41461000 -1.85118500 1.79295900

H -10.71033100 -0.77053200 2.31815900

C -5.62858400 1.98767700 -2.04539100

C -4.57379500 2.98363300 -1.52349900

H -4.98375600 3.58211900 -0.70386000

H -4.27731400 3.66618600 -2.32713700

H -3.67952700 2.47467000 -1.16266600

C -5.05318600 1.16476300 -3.21523400

H -4.18046600 0.58548600 -2.91194000

H -4.75327700 1.83371700 -4.02896200

H -5.80662600 0.47287000 -3.60465600

C -6.80496100 2.80924100 -2.59507200

H -7.58797300 2.17204300 -3.01843800

H -6.44218100 3.46030100 -3.39600400

H -7.25209600 3.44932700 -1.82775000

N -4.70382200 -1.50371600 1.43562100

O -3.85420000 0.21899600 -0.63776500

C 5.16637800 0.19302000 0.25762500

C 6.07181700 1.03257100 0.92690800

C 7.39140600 0.97794600 0.49017700

H 8.11844900 1.61145300 0.98178100

C 7.84516000 0.14699900 -0.55547500

C 6.92120400 -0.66076000 -1.18249000

H 7.18183200 -1.32950400 -1.99348100

C 5.56377600 -0.65989400 -0.79515100

C 3.40742300 -1.44055800 -1.04260600

C 2.44129900 -2.25529900 -1.66831000

H 2.78261700 -2.89806700 -2.47340200

C 1.11790000 -2.23778600 -1.27739900

C 0.71594200 -1.38048900 -0.22128300

C 1.64104400 -0.55635400 0.42737800

C 2.96890600 -0.60481800 0.00079400

C 9.32808500 0.17802700 -0.94306500

C 10.18225500 -0.22782900 0.27297700

H 11.24547000 -0.21588900 0.01024500

H 10.04085900 0.45410400 1.11663500

H 9.92356300 -1.23658500 0.60905600

C 9.70989000 1.60243800 -1.38843400

H 10.76647600 1.63957700 -1.67451700

H 9.11050700 1.91301500 -2.24968500

H 9.55595000 2.33452700 -0.59023300

C 9.64120000 -0.78638100 -2.09444600

H 9.41250700 -1.82273800 -1.82707700

H 9.08018200 -0.53170300 -2.99900800

H 10.70666700 -0.73361300 -2.33843300

C 5.63138200 1.95453200 2.07594700

C 5.06181800 1.11253200 3.23499500

H 5.81824400 0.41677700 3.61152800

H 4.76274300 1.76828200 4.05970100

H 4.18962800 0.53559100 2.92580600

C 4.57267300 2.95612800 1.57314200

H 3.67773500 2.45088400 1.20876600

H 4.27853100 3.62630400 2.38797000

H 4.97811000 3.56730000 0.76064600

C 6.80797900 2.77005100 2.63408300

H 7.25097600 3.42285900 1.87512600

H 6.44675600 3.40786000 3.44629600

H 7.59386500 2.12808100 3.04471800

N 4.70234700 -1.48352600 -1.45678400

O 3.85523900 0.20658100 0.64431600

C -0.10433600 -3.14127400 1.92520700

H 0.42015100 -3.77406000 1.20199400

H 0.66047500 -2.54670300 2.43565900

H -0.58270000 -3.78775300 2.66403600

C 1.26636100 0.37380400 1.55074900

H 0.21203600 0.27478800 1.81055000

H 1.86433000 0.17003000 2.44431300

H 1.45472800 1.41604300 1.27383900

C -1.26480700 0.39842600 -1.54024100

H -0.20773700 0.31095000 -1.79292700

H -1.85385500 0.20023600 -2.44105200

H -1.46421100 1.43564300 -1.25268100

C 0.10195500 -3.11172500 -1.96791600

H -0.42241400 -3.75544000 -1.25432500

H -0.66287000 -2.50895600 -2.46863900

H 0.57959600 -3.74687700 -2.71696600

**1d**-CS

C -5.33335800 0.24718100 0.14940900

C -6.36633200 1.01245500 0.71184800

C -7.65142700 0.49296100 0.57544800

H -8.47547200 1.05743300 0.99285600

C -7.94737100 -0.71825300 -0.07266300

C -6.89193200 -1.43648200 -0.60611700

H -7.02662700 -2.38140700 -1.11789400

C -5.57508000 -0.96789900 -0.50580700

C -3.34280100 -1.26188300 -0.94834300

C -2.22655800 -2.00028200 -1.45808300

H -2.46096800 -2.92265100 -1.97957300

C -0.94038700 -1.56551100 -1.34704100

C -0.66181600 -0.26586300 -0.75249700

C -1.76492700 0.49998800 -0.22508800

C -3.03299400 0.00311700 -0.33019000

C -9.40472300 -1.18731900 -0.15901800

C -9.97105400 -1.36248400 1.26280600

H -11.01086600 -1.70381000 1.21779200

H -9.95301000 -0.42531000 1.82685100

H -9.39117600 -2.10269200 1.82255300

C -10.23606800 -0.13581300 -0.91810800

H -11.27984000 -0.45924800 -0.99335700

H -9.84776100 0.00780800 -1.93106700

H -10.22424000 0.83454800 -0.41309500

C -9.53443900 -2.52660800 -0.89639200

H -8.98283900 -3.32332400 -0.38755600

H -9.16996100 -2.45817000 -1.92615100

H -10.58682600 -2.82412600 -0.93564100

C -6.09810000 2.34344100 1.43290100

C -5.18948000 2.10158900 2.65423700

H -5.66821500 1.41354500 3.35807600

H -5.00468300 3.04712500 3.17523300

H -4.22671900 1.67960800 2.36412800

C -5.43699000 3.34486400 0.46477600

H -4.47813100 2.97906200 0.09620400

H -5.26594500 4.29840100 0.97583800

H -6.08729600 3.53279700 -0.39539000

C -7.39650600 2.98647300 1.94416600

H -8.08812700 3.21855700 1.12796100

H -7.15459600 3.92699200 2.44813900

H -7.91265200 2.34853600 2.66869100

N -4.55782800 -1.73691000 -1.04605500

O -4.04570600 0.71051000 0.24122600

C 5.33339000 -0.24722500 0.14925000

C 6.36642400 -1.01233400 0.71180700

C 7.65146300 -0.49269500 0.57544100

H 8.47555000 -1.05703400 0.99294600

C 7.94729700 0.71851000 -0.07274000

C 6.89180500 1.43656500 -0.60631800

H 7.02642300 2.38146800 -1.11815700

C 5.57500200 0.96782700 -0.50605600

C 3.34270500 1.26156100 -0.94867500

C 2.22640800 1.99981500 -1.45849700

H 2.46073100 2.92212600 -1.98012400

C 0.94026500 1.56498200 -1.34732700

C 0.66179200 0.26541000 -0.75259000

C 1.76496700 -0.50033200 -0.22515800

C 3.03300000 -0.00339600 -0.33037500

C 9.40459200 1.18777000 -0.15900200

C 10.23613300 0.13638700 -0.91804500

H 11.27986200 0.45997700 -0.99323500

H 10.22442400 -0.83397700 -0.41303500

H 9.84790600 -0.00729100 -1.93102700

C 9.97079900 1.36299800 1.26286300

H 11.01057600 1.70444400 1.21792800

H 9.39079400 2.10313500 1.82257200

H 9.95282000 0.42581800 1.82690100

C 9.53417200 2.52708400 -0.89635500

H 9.16972500 2.45861700 -1.92612400

H 8.98246900 3.32373000 -0.38752200

H 10.58652600 2.82472400 -0.93557300

C 6.09832700 -2.34328300 1.43298000

C 5.43735200 -3.34489800 0.46496400

H 6.08766700 -3.53281600 -0.39519900

H 5.26647000 -4.29841100 0.97612500

H 4.47842600 -2.97929100 0.09637800

C 5.18966800 -2.10140500 2.65428000

H 4.22687300 -1.67954200 2.36411000

H 5.00494600 -3.04690800 3.17536300

H 5.66832300 -1.41324800 3.35806300

C 7.39680500 -2.98610900 1.94433000

H 7.91286400 -2.34804400 2.66880400

H 7.15499800 -3.92660900 2.44838800

H 8.08846500 -3.21818500 1.12815500

N 4.55769700 1.73667200 -1.04642900

O 4.04578400 -0.71069500 0.24102600

C 0.13241300 -2.39143000 -2.02067200

H 0.99242700 -1.79044800 -2.31876800

H 0.49317600 -3.18621500 -1.36214900

H -0.27700300 -2.87050400 -2.91279700

C 1.57243400 -1.73645300 0.61916200

H 0.51716500 -1.96080700 0.77365100

H 2.05445200 -2.61316500 0.17657300

H 2.03365800 -1.58831800 1.60075200

C -1.57229300 1.73618400 0.61910300

H -0.51700400 1.96058000 0.77339900

H -2.05439900 2.61284400 0.17651000

H -2.03335900 1.58811500 1.60077500

C -0.13263700 2.39075400 -2.02099100

H -0.99264500 1.78965200 -2.31887300

H -0.49338300 3.18560800 -1.36254000

H 0.27667000 2.86971400 -2.91322100

**TS** (**1a**-BS↔**1a’**-BS)

C 5.12916500 -0.14602100 3.75432100

C 6.01845400 -0.78274100 4.63439900

C 7.33346500 -0.32967200 4.60482100

H 8.04883200 -0.79635000 5.26965200

C 7.79688200 0.70218500 3.76176900

C 6.88771700 1.29836800 2.91496500

H 7.15633400 2.09740100 2.23511600

C 5.53571300 0.89304800 2.88810400

C 3.39869000 1.09415200 2.04457400

C 2.44164400 1.68488200 1.19180800

H 2.78057900 2.48074100 0.53776300

C 1.12840200 1.26105900 1.19677100

H 0.40294200 1.72437200 0.53614700

C 0.70981300 0.22698000 2.05357300

C 1.63502000 -0.37296800 2.90425900

H 1.34197500 -1.17265300 3.57620400

C 2.95362700 0.05881000 2.89428400

C 9.27335500 1.10994700 3.82390200

C 9.61004000 1.59335100 5.24729400

H 10.66150400 1.89409400 5.30628200

H 9.44621700 0.81040100 5.99356200

H 8.99166400 2.45361700 5.52114500

C 10.15346000 -0.10394700 3.47035200

H 11.21246500 0.17327700 3.50455100

H 9.92667600 -0.46722600 2.46335800

H 10.00450100 -0.93304300 4.16833500

C 9.59809600 2.24257400 2.84132200

H 9.01948600 3.14556200 3.05969300

H 9.40076200 1.94834700 1.80577700

H 10.65863500 2.50144800 2.91673600

C 5.56488300 -1.91241900 5.57383900

C 4.47739200 -1.39295000 6.53586400

H 4.85922600 -0.55800700 7.13184500

H 4.17855900 -2.19148500 7.22333800

H 3.58876400 -1.05533300 6.00133800

C 5.02502100 -3.09850800 4.74941800

H 4.15467900 -2.81829300 4.15500400

H 4.73157700 -3.91309000 5.42017100

H 5.79687700 -3.47832600 4.07260100

C 6.72663900 -2.43769700 6.43143800

H 7.53139900 -2.85734800 5.81931600

H 6.35751400 -3.23653100 7.08139900

H 7.14689500 -1.65806900 7.07489800

N 4.68893100 1.52532800 2.02697600

O 3.82314900 -0.55294200 3.74184500

C -5.12925800 0.14678600 3.75415500

C -6.01857500 0.78361900 4.63412400

C -7.33358500 0.33054900 4.60456000

H -8.04897200 0.79731100 5.26931000

C -7.79697800 -0.70141300 3.76162300

C -6.88778700 -1.29770400 2.91492200

H -7.15638400 -2.09682000 2.23516400

C -5.53578100 -0.89238900 2.88805400

C -3.39873300 -1.09360000 2.04461200

C -2.44166300 -1.68443400 1.19194500

H -2.78058000 -2.48037200 0.53798800

C -1.12842200 -1.26061100 1.19689300

H -0.40294300 -1.72400400 0.53634600

C -0.70985700 -0.22642800 2.05358100

C -1.63508700 0.37362400 2.90416900

H -1.34206100 1.17339100 3.57602400

C -2.95369400 -0.05815500 2.89420900

C -9.27345300 -1.10916600 3.82376000

C -10.15354600 0.10468700 3.47004000

H -11.21255300 -0.17253100 3.50424000

H -10.00460700 0.93386700 4.16792900

H -9.92673200 0.46784700 2.46301000

C -9.61018100 -1.59240000 5.24719900

H -10.66164700 -1.89313400 5.30619200

H -8.99181400 -2.45263400 5.52117000

H -9.44637800 -0.80936200 5.99337900

C -9.59816600 -2.24190900 2.84130500

H -9.40079800 -1.94780400 1.80573100

H -9.01956600 -3.14487200 3.05980100

H -10.65870800 -2.50077000 2.91671600

C -5.56503500 1.91341700 5.57343400

C -5.02513500 3.09939700 4.74888000

H -5.79696400 3.47913100 4.07198400

H -4.73171400 3.91406500 5.41953900

H -4.15477100 2.81910200 4.15453600

C -4.47758200 1.39406900 6.53556800

H -3.58893200 1.05638500 6.00112100

H -4.17877700 2.19269100 7.22295400

H -4.85944100 0.55920100 7.13163900

C -6.72682100 2.43881200 6.43092100

H -7.14709900 1.65927200 7.07447300

H -6.35771800 3.23773400 7.08078700

H -7.53155900 2.85838000 5.81871300

N -4.68897300 -1.52477800 2.02703000

O -3.82323900 0.55370000 3.74167100

**1a’**-BS

C 4.88595500 0.06330200 0.13163100

C 5.57284400 1.25605900 0.40667100

C 6.95404000 1.21472400 0.24291800

H 7.51831000 2.11599700 0.44539400

C 7.66835100 0.07210300 -0.17105900

C 6.94997500 -1.07651300 -0.42953400

H 7.41604300 -1.99955900 -0.75135100

C 5.54815300 -1.10997400 -0.28551800

C 3.55508900 -2.25938400 -0.39949500

C 2.78612200 -3.42190700 -0.64471300

H 3.31675800 -4.30922900 -0.97168200

C 1.41993700 -3.41743600 -0.48599700

H 0.85661500 -4.31617500 -0.71305900

C 0.73149000 -2.25213000 -0.07728300

C 1.47657600 -1.09192500 0.16931900

H 1.00262800 -0.18123200 0.51915000

C 2.84918100 -1.10211500 0.01009300

C 9.19252200 0.15490500 -0.31391100

C 9.54998400 1.23142200 -1.35624800

H 10.63679000 1.29852300 -1.47405500

H 9.18609400 2.22041700 -1.06252100

H 9.11407100 0.98829700 -2.33006800

C 9.81338500 0.52800100 1.04568000

H 10.90382400 0.58556500 0.96108900

H 9.56762600 -0.22237000 1.80329700

H 9.45660200 1.49775600 1.40492600

C 9.79986500 -1.17791000 -0.77064900

H 9.41447600 -1.48408400 -1.74819800

H 9.59922300 -1.98028700 -0.05383800

H 10.88574400 -1.07553500 -0.85908100

C 4.84231700 2.53053500 0.86104900

C 3.84157200 2.97943200 -0.22292200

H 4.36037700 3.17620500 -1.16643200

H 3.34570800 3.90431800 0.09066300

H 3.07329900 2.22691200 -0.40400500

C 4.10656700 2.27014500 2.19098100

H 3.34787300 1.49317800 2.09104300

H 3.61232500 3.18789700 2.52720600

H 4.81427900 1.96261400 2.96726300

C 5.82053400 3.69220100 1.09489900

H 6.54871800 3.46375700 1.87973700

H 5.25747800 4.57363700 1.41570000

H 6.36249200 3.96229300 0.18288300

N 4.89674300 -2.28192000 -0.55589000

O 3.52469600 0.04789200 0.27439400

C -4.88596400 0.06330500 -0.13162800

C -5.57284700 1.25606300 -0.40667400

C -6.95404400 1.21473300 -0.24292300

H -7.51831200 2.11600600 -0.44540100

C -7.66835800 0.07211300 0.17105200

C -6.94998700 -1.07650500 0.42952900

H -7.41605900 -1.99954900 0.75134400

C -5.54816400 -1.10997000 0.28551800

C -3.55510100 -2.25938000 0.39950200

C -2.78613600 -3.42190400 0.64471800

H -3.31677300 -4.30922600 0.97168500

C -1.41995100 -3.41743500 0.48600300

H -0.85663000 -4.31617500 0.71306400

C -0.73150200 -2.25212900 0.07729200

C -1.47658700 -1.09192200 -0.16930800

H -1.00263700 -0.18123000 -0.51913700

C -2.84919100 -1.10211000 -0.01008200

C -9.19253000 0.15491900 0.31389800

C -9.81338700 0.52801900 -1.04569300

H -10.90382600 0.58558800 -0.96110600

H -9.45659900 1.49777400 -1.40493700

H -9.56762800 -0.22235000 -1.80331200

C -9.54999200 1.23143500 1.35623700

H -10.63679900 1.29853900 1.47403900

H -9.11408300 0.98830600 2.33005700

H -9.18609800 2.22042900 1.06251200

C -9.79988000 -1.17789500 0.77063200

H -9.59923700 -1.98027100 0.05382000

H -9.41449500 -1.48407300 1.74818100

H -10.88575800 -1.07551600 0.85905900

C -4.84229600 2.53052700 -0.86104700

C -4.10659600 2.27013200 -2.19100400

H -4.81434500 1.96264800 -2.96727200

H -3.61232200 3.18786800 -2.52722600

H -3.34793300 1.49312800 -2.09110500

C -3.84149400 2.97935200 0.22290000

H -3.07325700 2.22678600 0.40394500

H -3.34559100 3.90421700 -0.09068400

H -4.36026100 3.17613500 1.16642900

C -5.82046900 3.69224200 -1.09484200

H -6.36238600 3.96234000 -0.18280300

H -5.25738500 4.57366000 -1.41564300

H -6.54868900 3.46384800 -1.87966200

N -4.89675600 -2.28191600 0.55589500

O -3.52470400 0.04789800 -0.27437900

**1a’**-T

C 4.90558400 0.05813000 0.16351000

C 5.60686000 1.22828100 0.49467300

C 6.98262700 1.19170900 0.29215000

H 7.55743800 2.07568900 0.53651100

C 7.67974400 0.07359000 -0.21282100

C 6.94967000 -1.05252900 -0.52485300

H 7.40183900 -1.95473900 -0.91761000

C 5.54942200 -1.09166500 -0.34656600

C 3.54099800 -2.21582500 -0.48379500

C 2.76046300 -3.35003300 -0.79128500

H 3.27601800 -4.21765600 -1.18803100

C 1.39418000 -3.34550300 -0.60404500

H 0.81202300 -4.22113300 -0.87195700

C 0.73452900 -2.20783500 -0.09879600

C 1.48809500 -1.07607000 0.21261000

H 1.02482700 -0.18810800 0.62973200

C 2.86090400 -1.08707100 0.01921300

C 9.20006100 0.16013600 -0.38986600

C 9.53586500 1.30073600 -1.36938000

H 10.61960000 1.37151700 -1.51081200

H 9.18487000 2.26997600 -1.00317400

H 9.07387300 1.12255000 -2.34521700

C 9.85743300 0.44215500 0.97450800

H 10.94560600 0.50126500 0.86550300

H 9.62733600 -0.35497000 1.68801200

H 9.51504000 1.38780100 1.40493400

C 9.78858900 -1.14226200 -0.94778100

H 9.37627000 -1.38297500 -1.93261100

H 9.60275300 -1.98886000 -0.27951900

H 10.87228400 -1.03787900 -1.05796500

C 4.89602100 2.47456200 1.04789400

C 3.86746700 2.99338000 0.02272300

H 4.36056500 3.24689100 -0.92103900

H 3.38526100 3.89849700 0.40720300

H 3.09067600 2.25602100 -0.18290600

C 4.19678400 2.13483700 2.37959000

H 3.43039900 1.36934600 2.25357300

H 3.71832600 3.03210900 2.78648500

H 4.92432200 1.77604200 3.11442200

C 5.88654200 3.61609800 1.32520200

H 6.63463200 3.33731400 2.07423500

H 5.33729000 4.47833900 1.71470800

H 6.40489200 3.93933700 0.41675700

N 4.88863900 -2.23697500 -0.67474200

O 3.54981000 0.03919600 0.34382200

C -4.90555200 0.05813400 -0.16349300

C -5.60684200 1.22828700 -0.49464700

C -6.98261000 1.19169100 -0.29214700

H -7.55742600 2.07566800 -0.53651300

C -7.67972100 0.07355900 0.21280000

C -6.94963900 -1.05255600 0.52482600

H -7.40180400 -1.95477700 0.91756100

C -5.54938900 -1.09167500 0.34655800

C -3.54096400 -2.21583600 0.48379600

C -2.76043000 -3.35004700 0.79127700

H -3.27598800 -4.21767700 1.18800400

C -1.39414500 -3.34551300 0.60404800

H -0.81199000 -4.22114700 0.87195000

C -0.73449300 -2.20783600 0.09882100

C -1.48805800 -1.07606800 -0.21257300

H -1.02478900 -0.18810000 -0.62968100

C -2.86086900 -1.08707400 -0.01918900

C -9.20004300 0.16007800 0.38981500

C -9.85738300 0.44217100 -0.97456000

H -10.94555800 0.50127600 -0.86557800

H -9.51497800 1.38784100 -1.40492500

H -9.62726800 -0.35491500 -1.68810200

C -9.53588700 1.30061700 1.36938800

H -10.61962600 1.37136800 1.51080600

H -9.07390800 1.12238400 2.34522300

H -9.18490600 2.26988500 1.00324500

C -9.78857200 -1.14235800 0.94763800

H -9.60272800 -1.98891100 0.27932200

H -9.37626000 -1.38313500 1.93245600

H -10.87226800 -1.03798700 1.05781900

C -4.89607100 2.47461800 -1.04785100

C -4.19680500 2.13499100 -2.37956100

H -4.92430300 1.77608600 -3.11437900

H -3.71849400 3.03233700 -2.78646900

H -3.43029800 1.36962000 -2.25356400

C -3.86759400 2.99356400 -0.02266200

H -3.09069700 2.25631900 0.18296200

H -3.38551800 3.89875800 -0.40712300

H -4.36073500 3.24699100 0.92110100

C -5.88671600 3.61604200 -1.32515400

H -6.40508800 3.93922700 -0.41670300

H -5.33756300 4.47834000 -1.71467500

H -6.63478800 3.33716500 -2.07417100

N -4.88860700 -2.23699000 0.67472400

O -3.54977500 0.03919300 -0.34379300

**1a’**-CS

C -4.85782900 0.08000100 0.00018900

C -5.52507600 1.31367700 0.00010300

C -6.91699300 1.25138100 -0.00002400

H -7.46784600 2.18345100 -0.00012000

C -7.65342400 0.05579300 -0.00003800

C -6.94487100 -1.13410100 0.00010400

H -7.42867700 -2.10309800 0.00013300

C -5.54614800 -1.13816900 0.00022000

C -3.59078000 -2.33617800 0.00037400

C -2.82011400 -3.55929800 0.00053100

H -3.38067100 -4.48726800 0.00079400

C -1.47064700 -3.54432700 0.00037700

H -0.95686500 -4.49693200 0.00059000

C -0.69730300 -2.31507500 0.00010500

C -1.46453500 -1.09239600 0.00012400

H -0.97945100 -0.12510300 0.00000100

C -2.81625600 -1.10584100 0.00024500

C -9.18567700 0.11556800 -0.00020000

C -9.66859700 0.86199100 1.25778900

H -10.76278500 0.90813700 1.27518800

H -9.29203400 1.88854400 1.29167600

H -9.33256700 0.35073200 2.16507500

C -9.66833400 0.86188000 -1.25835600

H -10.76251800 0.90803300 -1.27597900

H -9.33212700 0.35053400 -2.16552700

H -9.29175800 1.88842700 -1.29226400

C -9.81470200 -1.28394500 -0.00020700

H -9.52753500 -1.85634300 0.88744100

H -9.52730900 -1.85643200 -0.88772500

H -10.90550900 -1.19669800 -0.00034700

C -4.76647700 2.65087100 0.00011300

C -3.89108800 2.75976300 1.26493200

H -4.50909100 2.69559000 2.16607500

H -3.37442900 3.72550600 1.27630800

H -3.13873200 1.97139500 1.30777100

C -3.89097800 2.75970300 -1.26463000

H -3.13864500 1.97130800 -1.30738000

H -3.37428900 3.72543000 -1.27599600

H -4.50890500 2.69551600 -2.16582500

C -5.72844300 3.84902300 0.00005700

H -6.36695600 3.86229200 -0.88908600

H -5.14551300 4.77483400 0.00007600

H -6.36704200 3.86229400 0.88913700

N -4.88838700 -2.36562900 0.00037100

O -3.48569200 0.07717400 0.00025800

C 4.85782600 0.08000000 -0.00020700

C 5.52507400 1.31367600 -0.00011500

C 6.91699200 1.25137600 0.00004000

H 7.46784600 2.18344600 0.00014500

C 7.65342100 0.05578800 0.00007200

C 6.94486700 -1.13410500 -0.00008000

H 7.42867100 -2.10310200 -0.00009700

C 5.54614300 -1.13817000 -0.00022100

C 3.59077500 -2.33617900 -0.00039500

C 2.82011000 -3.55929900 -0.00055500

H 3.38066500 -4.48726900 -0.00081300

C 1.47064200 -3.54432800 -0.00041000

H 0.95686000 -4.49693300 -0.00062600

C 0.69729900 -2.31507500 -0.00014200

C 1.46453100 -1.09239700 -0.00016500

H 0.97944700 -0.12510300 -0.00005200

C 2.81625100 -1.10584200 -0.00027800

C 9.18567500 0.11556200 0.00026300

C 9.66830800 0.86188000 1.25842400

H 10.76249200 0.90803300 1.27606800

H 9.29173200 1.88842700 1.29232100

H 9.33208400 0.35053800 2.16559100

C 9.66861900 0.86197900 -1.25772100

H 10.76280700 0.90812300 -1.27510000

H 9.33260600 0.35071600 -2.16501000

H 9.29205800 1.88853200 -1.29161900

C 9.81469800 -1.28395200 0.00028900

H 9.52728800 -1.85643400 0.88780400

H 9.52754800 -1.85635300 -0.88736300

H 10.90550500 -1.19670600 0.00044900

C 4.76648500 2.65087500 -0.00014100

C 3.89097900 2.75972600 1.26459500

H 4.50889700 2.69553300 2.16579500

H 3.37430400 3.72546100 1.27595300

H 3.13863100 1.97134400 1.30734500

C 3.89110800 2.75977000 -1.26496800

H 3.13874000 1.97141500 -1.30780700

H 3.37446500 3.72552200 -1.27635600

H 4.50911700 2.69558100 -2.16610500

C 5.72846400 3.84901600 -0.00008500

H 6.36706600 3.86227800 -0.88916200

H 5.14554400 4.77483300 -0.00011000

H 6.36697300 3.86228300 0.88906100

N 4.88838200 -2.36563000 -0.00037800

O 3.48568800 0.07717200 -0.00029900

**1a’^2+^**

C 4.93982900 0.08576600 0.16752200

C 5.68348700 1.21704500 0.53833400

C 7.04414300 1.09875500 0.30386500

H 7.66407200 1.94488100 0.57291600

C 7.72137100 -0.03197800 -0.26014200

C 6.96416200 -1.11385200 -0.60803000

H 7.37762200 -2.01648100 -1.03964400

C 5.55379900 -1.08934700 -0.40509600

C 3.50434200 -2.12029600 -0.54758500

C 2.70191300 -3.23954200 -0.88875000

H 3.18998700 -4.10508200 -1.32179500

C 1.35228200 -3.19683200 -0.66905800

H 0.73720700 -4.04597600 -0.94532300

C 0.73389300 -2.04078400 -0.11275300

C 1.49337400 -0.93084800 0.22122600

H 1.05814200 -0.04703800 0.67400400

C 2.87109200 -0.98362000 0.00446100

C 9.23698400 0.03643600 -0.43741800

C 9.57675000 1.21636300 -1.37057400

H 10.65962700 1.26925800 -1.51033700

H 9.25667300 2.18004300 -0.96251800

H 9.11477000 1.08921300 -2.35390400

C 9.89077000 0.25184100 0.94266000

H 10.97702800 0.29432100 0.82786900

H 9.65474300 -0.56974100 1.62514000

H 9.57895500 1.18950800 1.41296900

C 9.79052300 -1.25523700 -1.05125700

H 9.37649700 -1.44522100 -2.04663100

H 9.59882800 -2.12527600 -0.41522800

H 10.87367700 -1.16729500 -1.16213000

C 5.04514400 2.47393400 1.15151000

C 4.03202100 3.07986400 0.15880500

H 4.51545200 3.33423200 -0.78889100

H 3.61941300 4.00023700 0.58083500

H 3.19567000 2.40875500 -0.04859600

C 4.35217100 2.10941400 2.48038900

H 3.52607800 1.40814200 2.34463400

H 3.94346400 3.01568300 2.93537800

H 5.06385300 1.67308200 3.18710300

C 6.10804000 3.54179400 1.45113000

H 6.84900000 3.19560500 2.17851800

H 5.61746900 4.41655700 1.88400900

H 6.62517400 3.87696100 0.54651800

N 4.83500300 -2.14886300 -0.74496100

O 3.61483400 0.07678800 0.34233500

C -4.93982700 0.08576900 -0.16751700

C -5.68348300 1.21704800 -0.53833100

C -7.04414100 1.09875700 -0.30387000

H -7.66406900 1.94488400 -0.57292300

C -7.72137200 -0.03197900 0.26012900

C -6.96416400 -1.11385300 0.60801800

H -7.37762700 -2.01648400 1.03962700

C -5.55380000 -1.08934700 0.40509300

C -3.50434400 -2.12029600 0.54759200

C -2.70191600 -3.23954400 0.88875500

H -3.18999200 -4.10508500 1.32179400

C -1.35228400 -3.19683300 0.66906900

H -0.73721100 -4.04597900 0.94533100

C -0.73389300 -2.04078300 0.11277000

C -1.49337200 -0.93084500 -0.22120600

H -1.05813800 -0.04703400 -0.67397800

C -2.87109000 -0.98361800 -0.00444600

C -9.23698600 0.03643400 0.43739500

C -9.89076300 0.25183900 -0.94268700

H -10.97702200 0.29431800 -0.82790300

H -9.57894700 1.18950800 -1.41299200

H -9.65473000 -0.56974100 -1.62516700

C -9.57676000 1.21635900 1.37055000

H -10.65963800 1.26925300 1.51030600

H -9.11478700 1.08920800 2.35388400

H -9.25668100 2.18004000 0.96249700

C -9.79052800 -1.25524100 1.05122900

H -9.59882700 -2.12527900 0.41520100

H -9.37650800 -1.44522600 2.04660600

H -10.87368300 -1.16730000 1.16209400

C -5.04514000 2.47394000 -1.15150000

C -4.35219800 2.10943700 -2.48040000

H -5.06389700 1.67311700 -3.18710400

H -3.94350000 3.01571200 -2.93538600

H -3.52610400 1.40816200 -2.34467400

C -4.03199300 3.07984500 -0.15880500

H -3.19564500 2.40872400 0.04857300

H -3.61938200 4.00022000 -0.58082800

H -4.51540400 3.33420400 0.78890500

C -6.10803500 3.54181200 -1.45108000

H -6.62514100 3.87697300 -0.54644900

H -5.61746800 4.41657600 -1.88396300

H -6.84901800 3.19563600 -2.17845000

N -4.83500600 -2.14886400 0.74495900

O -3.61483100 0.07679200 -0.34232000
