# Supplementary material for: Design of an open-shell nitrogen-centered diradicaloid with tunable stimuli-responsive electronic properties
Source: Commun Chem. 2022 Oct 14;5:127. doi: 10.1038/s42004-022-00747-8 (PMC9814612; doi:10.1038/s42004-022-00747-8)
Supplement: Supplementary file 5 — Supplementary Data 2 [file 42004_2022_747_MOESM5_ESM.zip › Supplementary Data 2/Supplementary Data 2.pdf]

checkCIF (basic structural check) running

Checking for embedded fcf data in CIF ...
Found embedded fcf data in CIF. Extracting fcf data from uploaded CIF, please wait . .

checkCIF/PLATON (basic structural check)

Structure factors have been supplied for datablock(s) exp_1391

THIS REPORT IS FOR GUIDANCE ONLY. IF USED AS PART OF A REVIEW PROCEDURE FOR PUBLICATION, IT SHOULD NOT REPLACE THE EXPERTISE OF AN EXPERIENCED CRYSTALLOGRAPHIC REFEREE.

No syntax errors found. CIF dictionary
Please wait while processing .... Interpreting this report

Structure factor report

Datablock: exp_1391

Bond precision: C-C = 0.0040 A Wavelength=1.54184

Cell: a=6.0491(3) b=18.1428(5) c=15.3109(5)
alpha=90 beta=97.068(4) gamma=90

Temperature: 100 K

	Calculated	Reported
Volume	1667.57(11)	1667.57(11)
Space group	P 21/n	P 1 21/n 1
Hall group	-P 2yn	-P 2yn
Moiety formula	C40 H46 N2 O2	C40 H46 N2 O2
Sum formula	C40 H46 N2 O2	C40 H46 N2 O2
Mr	586.79	586.79
Dx,g cm-3	1.169	1.169
Z	2	2
Mu (mm-1)	0.549	0.549
F000	632.0	632.0
F000'	633.68	
h,k,lmax	7,21,18	7,21,18
Nref	2979	2965
Tmin,Tmax	0.924,0.957	0.585,1.000
Tmin'	0.839	

Correction method= # Reported T Limits: Tmin=0.585 Tmax=1.000 AbsCorr = MULTI-SCAN

Data completeness= 0.995 Theta(max)= 67.058

R(reflections)= 0.0758(2029) wR2(reflections)= 0.2387(2965)

S = 1.033 Npar= 205

The following ALERTS were generated. Each ALERT has the format
test-name_ALERT_alert-type_alert-level.
Click on the hyperlinks for more details of the test.

Alert level C

RINTA01_ALERT_3_C The value of Rint is greater than 0.12
Rint given 0.133
PLAT020_ALERT_3_C The Value of Rint is Greater Than 0.12 .... 0.133 Report

PLAT213_ALERT_2_C Atom C14 has ADP max/min Ratio ..... 3.6 prolat
PLAT213_ALERT_2_C Atom C16 has ADP max/min Ratio ..... 3.5 prolat
PLAT220_ALERT_2_C NonSolvent Resd 1 C Ueq(max)/Ueq(min) Range 3.4 Ratio
PLAT242_ALERT_2_C Low 'MainMol' Ueq as Compared to Neighbors of C13 Check
PLAT250_ALERT_2_C Large U3/U1 Ratio for Average U(i,j) Tensor .... 3.3 Note
PLAT790_ALERT_4_C Centre of Gravity not Within Unit Cell: Resd. # 1 Note
C40 H46 N2 O2
PLAT906_ALERT_3_C Large K Value in the Analysis of Variance ...... 4.829 Check
PLAT911_ALERT_3_C Missing FCF Refl Between Thmin & STh/L= 0.597 14 Report

---

Alert level G

PLAT003_ALERT_2_G Number of Uiso or Uij Restrained non-H Atoms ... 1 Report
PLAT072_ALERT_2_G SHELXL First Parameter in WGHT Unusually Large 0.15 Report
PLAT186_ALERT_4_G The CIF-Embedded .res File Contains ISOR Records 1 Report
PLAT804_ALERT_5_G Number of ARU-Code Packing Problem(s) in PLATON 2 Info
PLAT860_ALERT_3_G Number of Least-Squares Restraints .... 6 Note
PLAT909_ALERT_3_G Percentage of I>2sig(I) Data at Theta(Max) Still 47% Note
PLAT933_ALERT_2_G Number of OMIT Records in Embedded .res File ... 13 Note
PLAT941_ALERT_3_G Average HKL Measurement Multiplicity .... 4.8 Low
PLAT978_ALERT_2_G Number C-C Bonds with Positive Residual Density. 1 Info

---

0 **ALERT level A** = Most likely a serious problem - resolve or explain
0 **ALERT level B** = A potentially serious problem, consider carefully
10 **ALERT level C** = Check. Ensure it is not caused by an omission or oversight
9 **ALERT level G** = General information/check it is not something unexpected

0 ALERT type 1 CIF construction/syntax error, inconsistent or missing data
9 ALERT type 2 Indicator that the structure model may be wrong or deficient
7 ALERT type 3 Indicator that the structure quality may be low
2 ALERT type 4 Improvement, methodology, query or suggestion
1 ALERT type 5 Informative message, check

It is advisable to attempt to resolve as many as possible of the alerts in all categories. Often the minor alerts point to easily fixed oversights, errors and omissions in your CIF or refinement strategy, so attention to these fine details can be worthwhile. In order to resolve some of the more serious problems it may be necessary to carry out additional measurements or structure refinements. However, the purpose of your study may justify the reported deviations and the more serious of these should normally be commented upon in the discussion or experimental section of a paper or in the "special_details" fields of the CIF. checkCIF was carefully designed to identify outliers and unusual parameters, but every test has its limitations and alerts that are not important in a particular case may appear. Conversely, the absence of alerts does not guarantee there are no aspects of the results needing attention. It is up to the individual to critically assess their own results and, if necessary, seek expert advice.

Publication of your CIF in IUCr journals

A basic structural check has been run on your CIF. These basic checks will be run on all CIFs submitted for publication in IUCr journals (*Acta Crystallographica*, *Journal of Applied Crystallography*, *Journal of Synchrotron Radiation*); however, if you intend to submit to *Acta Crystallographica Section C* or *E* or *IUCrData*, you should make sure that full publication checks are run on the final version of your CIF prior to submission.

Publication of your CIF in other journals

Please refer to the *Notes for Authors* of the relevant journal for any special instructions relating to CIF submission.

PLATON version of 10/08/2020; check.def file version of 06/08/2020

Datablock exp_1391 - ellipsoid plot

Download CIF editor (publCIF) from the IUCr
Download CIF editor (enCIFer) from the CCDC
Test a new CIF entry
