# Supplementary material for: Design of an open-shell nitrogen-centered diradicaloid with tunable stimuli-responsive electronic properties
Source: Commun Chem. 2022 Oct 14;5:127. doi: 10.1038/s42004-022-00747-8 (PMC9814612; doi:10.1038/s42004-022-00747-8)

checkCIF/PLATON report

Structure factors have been supplied for datablock(s) exp_1931

THIS REPORT IS FOR GUIDANCE ONLY. IF USED AS PART OF A REVIEW PROCEDURE FOR PUBLICATION, IT SHOULD NOT REPLACE THE EXPERTISE OF AN EXPERIENCED CRYSTALLOGRAPHIC REFEREE.

No syntax errors found. CIF dictionary Interpreting this report

Datablock: exp_1931

Bond precision:	C-C = 0.0101 A		Wavelength=1.54184
Cell:	a=40.823(2)	b=9.4048(4)	c=17.7684(7)
	alpha=90	beta=98.280(5)	gamma=90
Temperature:	170 K		

	Calculated	Reported
Volume	6750.8(5)	6750.7(5)
Space group	C 2/c	C 1 2/c 1
Hall group	-C 2yc	-C 2yc
Moiety formula	C40 H46 N2 O2, 2(Cl6 Sb) [+ solvent]	Cl6 Sb, C20 H23 N O
Sum formula	C40 H46 Cl12 N2 O2 Sb2 [+ solvent]	C20 H23 Cl6 N O Sb
Mr	1255.71	627.84
Dx,g cm-3	1.235	1.235
Z	4	8
Mu (mm-1)	10.930	10.930
F000	2488.0	2488.0
F000’	2509.74	
h,k,lmax	48,11,21	48,11,21
Nref	6023	5979
Tmin,Tmax	0.259,0.301	0.035,1.000
Tmin’	0.016	

Correction method= # Reported T Limits: Tmin=0.035 Tmax=1.000
AbsCorr = MULTI-SCAN

Data completeness= 0.993 Theta(max)= 67.075

R(reflections)= 0.0825(3893) wR2(reflections)= 0.2312(5979)

S = 1.007 Npar= 268

```
The following ALERTS were generated. Each ALERT has the format
       test-name_ALERT_alert-type_alert-level.
Click on the hyperlinks for more details of the test.
```

Alert level B

```
PLAT973_ALERT_2_B Check Calcd Positive Resid. Density on       Sb1       1.75 eA-3
```

Alert level C

```
RINTA01_ALERT_3_C  The value of Rint is greater than 0.12
            Rint given   0.160
PLAT020_ALERT_3_C The Value of Rint is Greater Than 0.12 .........      0.160 Report
PLAT094_ALERT_2_C Ratio of Maximum / Minimum Residual Density ....       2.84 Report
PLAT234_ALERT_4_C Large Hirshfeld Difference C13      --C15      .       0.16 Ang.
PLAT242_ALERT_2_C Low    'MainMol' Ueq as Compared to Neighbors of        C13 Check
PLAT242_ALERT_2_C Low    'MainMol' Ueq as Compared to Neighbors of        C17 Check
PLAT244_ALERT_4_C Low    'Solvent' Ueq as Compared to Neighbors of        Sb1 Check
PLAT342_ALERT_3_C Low Bond Precision on  C-C Bonds ...............    0.01014 Ang.
PLAT906_ALERT_3_C Large K Value in the Analysis of Variance ......      3.312 Check
PLAT911_ALERT_3_C Missing FCF Refl Between Thmin & STh/L=    0.597         43 Report
PLAT975_ALERT_2_C Check Calcd Resid. Dens.  1.09A   From N1              0.53 eA-3
```

Alert level G

```
PLAT042_ALERT_1_G Calc. and Reported Moiety Formula Strings Differ     Please Check
PLAT045_ALERT_1_G Calculated and Reported Z Differ by a Factor ...       0.50 Check
PLAT072_ALERT_2_G SHELXL First  Parameter in WGHT  Unusually Large       0.13 Report
PLAT606_ALERT_4_G Solvent Accessible VOID(S) in Structure ........          ! Info
PLAT720_ALERT_4_G Number of Unusual/Non-Standard Labels ..........          1 Note
PLAT794_ALERT_5_G Tentative Bond Valency for Sb1       (V)       .       5.12 Info
PLAT909_ALERT_3_G Percentage of I>2sig(I) Data at Theta(Max) Still        34% Note
PLAT910_ALERT_3_G Missing # of FCF Reflection(s) Below Theta(Min).          1 Note
PLAT933_ALERT_2_G Number of OMIT Records in Embedded .res File ...         42 Note
PLAT978_ALERT_2_G Number C-C Bonds with Positive Residual Density.          0 Info
```

```
   0 ALERT level A = Most likely a serious problem - resolve or explain
   1 ALERT level B = A potentially serious problem, consider carefully
  11 ALERT level C = Check. Ensure it is not caused by an omission or oversight
  10 ALERT level G = General information/check it is not something unexpected

   2 ALERT type 1 CIF construction/syntax error, inconsistent or missing data
   8 ALERT type 2 Indicator that the structure model may be wrong or deficient
   7 ALERT type 3 Indicator that the structure quality may be low
   4 ALERT type 4 Improvement, methodology, query or suggestion
   1 ALERT type 5 Informative message, check
```

It is advisable to attempt to resolve as many as possible of the alerts in all categories. Often the minor alerts point to easily fixed oversights, errors and omissions in your CIF or refinement strategy, so attention to these fine details can be worthwhile. In order to resolve some of the more serious problems it may be necessary to carry out additional measurements or structure refinements. However, the purpose of your study may justify the reported deviations and the more serious of these should normally be commented upon in the discussion or experimental section of a paper or in the "special_details" fields of the CIF. checkCIF was carefully designed to identify outliers and unusual parameters, but every test has its limitations and alerts that are not important in a particular case may appear. Conversely, the absence of alerts does not guarantee there are no aspects of the results needing attention. It is up to the individual to critically assess their own results and, if necessary, seek expert advice.

Publication of your CIF in IUCr journals

A basic structural check has been run on your CIF. These basic checks will be run on all CIFs submitted for publication in IUCr journals (*Acta Crystallographica*, *Journal of Applied Crystallography*, *Journal of Synchrotron Radiation*); however, if you intend to submit to *Acta Crystallographica Section C* or *E* or *IUCrData*, you should make sure that full publication checks are run on the final version of your CIF prior to submission.

Publication of your CIF in other journals

Please refer to the *Notes for Authors* of the relevant journal for any special instructions relating to CIF submission.

PLATON version of 22/03/2021; check.def file version of 19/03/2021

Datablock exp_1931 - ellipsoid plot

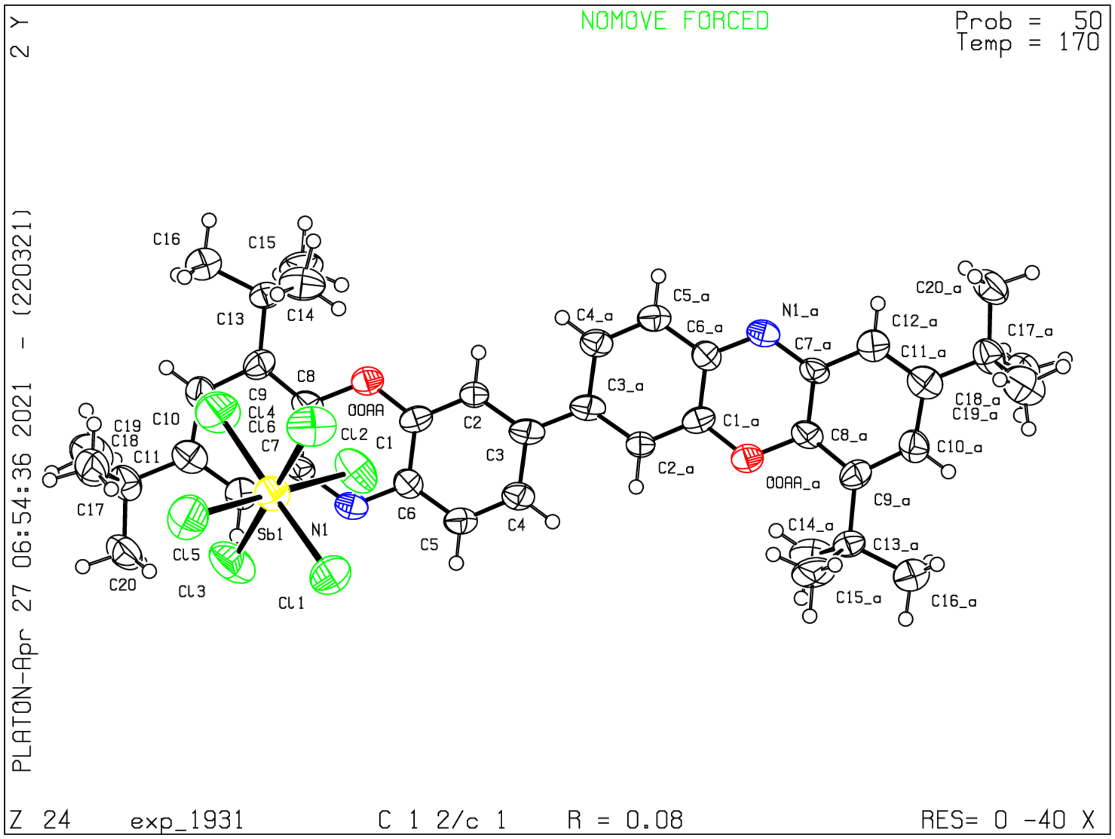

Supplement: Supplementary file 6 — Supplementary Data 3 [file 42004_2022_747_MOESM6_ESM.zip › Supplementary Data 3/Supplementary Data 3.pdf]
