# Supplementary material for: Design of an open-shell nitrogen-centered diradicaloid with tunable stimuli-responsive electronic properties
Source: Commun Chem. 2022 Oct 14;5:127. doi: 10.1038/s42004-022-00747-8 (PMC9814612; doi:10.1038/s42004-022-00747-8)

checkCIF/PLATON report

Structure factors have been supplied for datablock(s) exp_2148

THIS REPORT IS FOR GUIDANCE ONLY. IF USED AS PART OF A REVIEW PROCEDURE FOR PUBLICATION, IT SHOULD NOT REPLACE THE EXPERTISE OF AN EXPERIENCED CRYSTALLOGRAPHIC REFEREE.

No syntax errors found.   CIF dictionary   Interpreting this report

Datablock: exp_2148

```
Bond precision:  C-C = 0.0060 A          Wavelength=1.54184

Cell:          a=5.8080(7)       b=9.2231(12)      c=16.3047(10)
               alpha=97.258(8)   beta=100.154(7)   gamma=90.988(10)
Temperature:   170 K

                Calculated                 Reported
Volume          852.14(16)                 852.14(16)
Space group     P -1                       P -1
Hall group      -P 1                       -P 1
Moiety formula  C42 H50 N2 O2              C42 H50 N2 O2
Sum formula     C42 H50 N2 O2              C42 H50 N2 O2
Mr              614.84                     614.84
Dx,g cm-3       1.198                      1.198
Z               1                          1
Mu (mm-1)       0.559                      0.559
F000            332.0                      332.0
F000'           332.87
h,k,lmax        6,11,19                    6,11,19
Nref            3023                       2966
Tmin,Tmax       0.941,0.962                0.782,1.000
Tmin'           0.865

Correction method= # Reported T Limits: Tmin=0.782 Tmax=1.000
AbsCorr = MULTI-SCAN

Data completeness= 0.981          Theta(max)= 67.028

R(reflections)= 0.1112( 2415)     wR2(reflections)= 0.3191( 2966)

S = 1.093                 Npar= 215
```

The following ALERTS were generated. Each ALERT has the format
test-name_ALERT_alert-type_alert-level.
Click on the hyperlinks for more details of the test.

Alert level B

```
PLAT930_ALERT_2_B FCF-based   Twin Law         [ 1 0 0] Est.d BASF       0.25 Check
```

Alert level C

```
RINTA01_ALERT_3_C  The value of Rint is greater than 0.12
            Rint given   0.128
PLAT020_ALERT_3_C The Value of Rint is Greater Than 0.12 .........      0.128 Report
PLAT082_ALERT_2_C High R1 Value ..................................       0.11 Report
PLAT084_ALERT_3_C High wR2 Value (i.e. > 0.25) ...................       0.32 Report
PLAT340_ALERT_3_C Low Bond Precision on  C-C Bonds ...............    0.00595 Ang.
PLAT790_ALERT_4_C Centre of Gravity not Within Unit Cell: Resd.  #          1 Note
              C42 H50 N2 O2
PLAT906_ALERT_3_C Large K Value in the Analysis of Variance ......     15.532 Check
PLAT906_ALERT_3_C Large K Value in the Analysis of Variance ......      3.492 Check
PLAT906_ALERT_3_C Large K Value in the Analysis of Variance ......      2.087 Check
PLAT911_ALERT_3_C Missing FCF Refl Between Thmin & STh/L=    0.597         57 Report
```

Alert level G

```
PLAT072_ALERT_2_G SHELXL First  Parameter in WGHT  Unusually Large       0.15 Report
PLAT804_ALERT_5_G Number of ARU-Code Packing Problem(s) in PLATON           1 Info
PLAT870_ALERT_4_G ALERTS Related to Twinning Effects Suppressed ..          ! Info
PLAT909_ALERT_3_G Percentage of I>2sig(I) Data at Theta(Max) Still        68% Note
PLAT910_ALERT_3_G Missing # of FCF Reflection(s) Below Theta(Min).          1 Note
PLAT931_ALERT_5_G CIFcalcFCF  Twin Law         [ 1 0 0] Est.d BASF       0.25 Check
PLAT933_ALERT_2_G Number of OMIT Records in Embedded .res File ...         49 Note
```

0 **ALERT level A** = Most likely a serious problem - resolve or explain
1 **ALERT level B** = A potentially serious problem, consider carefully
10 **ALERT level C** = Check. Ensure it is not caused by an omission or oversight
7 **ALERT level G** = General information/check it is not something unexpected

0 ALERT type 1 CIF construction/syntax error, inconsistent or missing data
4 ALERT type 2 Indicator that the structure model may be wrong or deficient
10 ALERT type 3 Indicator that the structure quality may be low
2 ALERT type 4 Improvement, methodology, query or suggestion
2 ALERT type 5 Informative message, check

It is advisable to attempt to resolve as many as possible of the alerts in all categories. Often the minor alerts point to easily fixed oversights, errors and omissions in your CIF or refinement strategy, so attention to these fine details can be worthwhile. In order to resolve some of the more serious problems it may be necessary to carry out additional measurements or structure refinements. However, the purpose of your study may justify the reported deviations and the more serious of these should normally be commented upon in the discussion or experimental section of a paper or in the "special_details" fields of the CIF. checkCIF was carefully designed to identify outliers and unusual parameters, but every test has its limitations and alerts that are not important in a particular case may appear. Conversely, the absence of alerts does not guarantee there are no aspects of the results needing attention. It is up to the individual to critically assess their own results and, if necessary, seek expert advice.

Publication of your CIF in IUCr journals

A basic structural check has been run on your CIF. These basic checks will be run on all CIFs submitted for publication in IUCr journals (*Acta Crystallographica*, *Journal of Applied Crystallography*, *Journal of Synchrotron Radiation*); however, if you intend to submit to *Acta Crystallographica Section C* or *E* or *IUCrData*, you should make sure that full publication checks are run on the final version of your CIF prior to submission.

Publication of your CIF in other journals

Please refer to the *Notes for Authors* of the relevant journal for any special instructions relating to CIF submission.

PLATON version of 13/07/2021; check.def file version of 13/07/2021

Datablock exp_2148 - ellipsoid plot

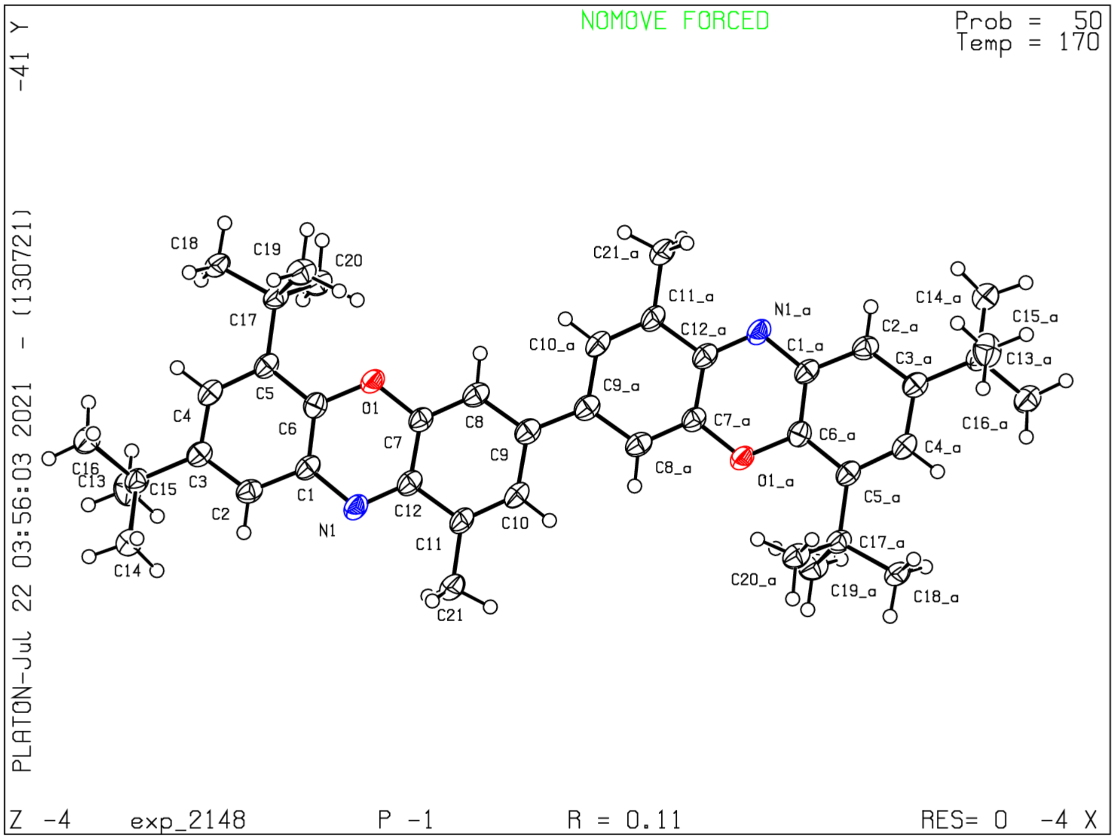

Supplement: Supplementary file 7 — Supplementary Data 4 [file 42004_2022_747_MOESM7_ESM.zip › Supplementary Data 4/Supplementary Data 4.pdf]
