# Supplementary material for: Design of an open-shell nitrogen-centered diradicaloid with tunable stimuli-responsive electronic properties
Source: Commun Chem. 2022 Oct 14;5:127. doi: 10.1038/s42004-022-00747-8 (PMC9814612; doi:10.1038/s42004-022-00747-8)
Supplement: Supplementary file 8 — Supplementary Data 5 [file 42004_2022_747_MOESM8_ESM.zip › Supplementary Data 5/Supplementary Data 5.pdf]

checkCIF (basic structural check) running

Checking for embedded fcf data in CIF ...
Found embedded fcf data in CIF. Extracting fcf data from uploaded CIF, please wait . .

checkCIF/PLATON (basic structural check)

Structure factors have been supplied for datablock(s) exp_1355

THIS REPORT IS FOR GUIDANCE ONLY. IF USED AS PART OF A REVIEW PROCEDURE FOR PUBLICATION, IT SHOULD NOT REPLACE THE EXPERTISE OF AN EXPERIENCED CRYSTALLOGRAPHIC REFEREE.

No syntax errors found.
Please wait while processing ....

CIF dictionary
Interpreting this report

Structure factor report

Datablock: exp_1355

Bond precision:	C-C = 0.0057 A		Wavelength=1.54184
Cell:	a=19.1147(4)	b=10.0807(2)	c=9.0952(2)
	alpha=90	beta=97.220(2)	gamma=90
Temperature: 100 K			

	Calculated	Reported
Volume	1738.65(6)	1738.65(6)
Space group	P 21/c	P 1 21/c 1
Hall group	-P 2ybc	-P 2ybc
Moiety formula	C40 H50 N2 O2 [+ solvent]	C40 H50 N2 O2
Sum formula	C40 H50 N2 O2 [+ solvent]	C40 H50 N2 O2
Mr	590.82	590.82
Dx,g cm-3	1.129	1.129
Z	2	2
Mu (mm-1)	0.527	0.527
F000	640.0	640.0
F000'	641.68	
h,k,lmax	22,12,10	22,12,10
Nref	3096	3032
Tmin,Tmax	0.848,0.910	0.669,1.000
Tmin'	0.801	

Correction method= # Reported T Limits: Tmin=0.669 Tmax=1.000 AbsCorr = MULTI-SCAN

Data completeness= 0.979 Theta(max)= 67.078

R(reflections)= 0.1029(2898) wR2(reflections)= 0.2749(3032)

S = 1.116 Npar= 215

The following ALERTS were generated. Each ALERT has the format
test-name_ALERT_alert-type_alert-level.
Click on the hyperlinks for more details of the test.

Alert level B

PLAT930_ALERT_2_B FCF-based Twin Law (1 0 0)[4 0 1] Est.d BASF 0.20 Check

Alert level C

DIFMX02_ALERT_1_C The maximum difference density is > 0.1*ZMAX*0.75
The relevant atom site should be identified.
PLAT029_ALERT_3_C _diffrn_measured_fraction_theta_full value Low . 0.979 Why?
PLAT084_ALERT_3_C High wR2 Value (i.e. > 0.25) .... 0.27 Report
PLAT097_ALERT_2_C Large Reported Max. (Positive) Residual Density 0.64 eA-3
PLAT242_ALERT_2_C Low 'MainMol' Ueq as Compared to Neighbors of C17 Check
PLAT340_ALERT_3_C Low Bond Precision on C-C Bonds .... 0.00571 Ang.
PLAT420_ALERT_2_C D-H Without Acceptor N1 --H1 . Please Check
PLAT906_ALERT_3_C Large K Value in the Analysis of Variance ...... 10.831 Check
PLAT906_ALERT_3_C Large K Value in the Analysis of Variance ...... 2.393 Check
PLAT911_ALERT_3_C Missing FCF Refl Between Thmin & STh/L= 0.597 63 Report

---

Alert level G

PLAT007_ALERT_5_G Number of Unrefined Donor-H Atoms .... 3 Report
PLAT083_ALERT_2_G SHELXL Second Parameter in WGHT Unusually Large 6.95 Why ?
PLAT300_ALERT_4_G Atom Site Occupancy of O1 Constrained at 0.5 Check
And 3 other PLAT300 Alerts
More ...
PLAT301_ALERT_3_G Main Residue Disorder ....(Resd 1) 5% Note
PLAT605_ALERT_4_G Largest Solvent Accessible VOID in the Structure 17 A**3
PLAT870_ALERT_4_G ALERTS Related to Twinning Effects Suppressed .. ! Info
PLAT909_ALERT_3_G Percentage of I>2sig(I) Data at Theta(Max) Still 90% Note
PLAT910_ALERT_3_G Missing # of FCF Reflection(s) Below Theta(Min). 2 Note
PLAT931_ALERT_5_G CIFcalcFCF Twin Law [4 0 1] Est.d BASF 0.20 Check
PLAT933_ALERT_2_G Number of OMIT Records in Embedded .res File ... 64 Note
PLAT992_ALERT_5_G Repd & Actual _reflns_number_gt Values Differ by 1 Check

---

0 **ALERT level A** = Most likely a serious problem - resolve or explain
1 **ALERT level B** = A potentially serious problem, consider carefully
10 **ALERT level C** = Check. Ensure it is not caused by an omission or oversight
14 **ALERT level G** = General information/check it is not something unexpected

1 ALERT type 1 CIF construction/syntax error, inconsistent or missing data
6 ALERT type 2 Indicator that the structure model may be wrong or deficient
9 ALERT type 3 Indicator that the structure quality may be low
6 ALERT type 4 Improvement, methodology, query or suggestion
3 ALERT type 5 Informative message, check

It is advisable to attempt to resolve as many as possible of the alerts in all categories. Often the minor alerts point to easily fixed oversights, errors and omissions in your CIF or refinement strategy, so attention to these fine details can be worthwhile. In order to resolve some of the more serious problems it may be necessary to carry out additional measurements or structure refinements. However, the purpose of your study may justify the reported deviations and the more serious of these should normally be commented upon in the discussion or experimental section of a paper or in the "special_details" fields of the CIF. checkCIF was carefully designed to identify outliers and unusual parameters, but every test has its limitations and alerts that are not important in a particular case may appear. Conversely, the absence of alerts does not guarantee there are no aspects of the results needing attention. It is up to the individual to critically assess their own results and, if necessary, seek expert advice.

Publication of your CIF in IUCr journals

A basic structural check has been run on your CIF. These basic checks will be run on all CIFs submitted for publication in IUCr journals (*Acta Crystallographica*, *Journal of Applied Crystallography*, *Journal of Synchrotron Radiation*); however, if you intend to submit to *Acta Crystallographica Section C* or *E* or *IUCrData*, you should make sure that full publication checks are run on the final version of your CIF prior to submission.

Publication of your CIF in other journals

Please refer to the *Notes for Authors* of the relevant journal for any special instructions relating to CIF submission.

PLATON version of 16/07/2020; check.def file version of 12/07/2020

Datablock exp_1355 - ellipsoid plot

Download CIF editor (publCIF) from the IUCr
Download CIF editor (enCIFer) from the CCDC
Test a new CIF entry
