# Supplementary material for: Design of an open-shell nitrogen-centered diradicaloid with tunable stimuli-responsive electronic properties
Source: Commun Chem. 2022 Oct 14;5:127. doi: 10.1038/s42004-022-00747-8 (PMC9814612; doi:10.1038/s42004-022-00747-8)
Supplement: Supplementary file 10 — Supplementary Data 7 [file 42004_2022_747_MOESM10_ESM.zip › Supplementary Data 7/Supplementary Data 7.pdf]

checkCIF (basic structural check) running

Checking for embedded fcf data in CIF ...
Found embedded fcf data in CIF. Extracting fcf data from uploaded CIF, please wait **. . .**

checkCIF/PLATON (basic structural check)

Structure factors have been supplied for datablock(s) exp_1456

THIS REPORT IS FOR GUIDANCE ONLY. IF USED AS PART OF A REVIEW PROCEDURE FOR PUBLICATION, IT SHOULD NOT REPLACE THE EXPERTISE OF AN EXPERIENCED CRYSTALLOGRAPHIC REFEREE.

No syntax errors found.
Please wait while processing ....

CIF dictionary
Interpreting this report

Structure factor report

Datablock: exp_1456

Bond precision: C-C = 0.0035 A Wavelength=1.54184

Cell:	a=10.2479(3)	b=12.4899(4)	c=21.9716(7)
	alpha=74.823(3)	beta=79.678(3)	gamma=73.455(3)

Temperature: 293 K

	Calculated	Reported
Volume	2585.11(15)	2585.11(15)
Space group	P -1	P -1
Hall group	-P 1	-P 1
Moiety formula	C44 H60 N2 O2 [+ solvent]	C44 H60 N2 O2
Sum formula	C44 H60 N2 O2 [+ solvent]	C44 H60 N2 O2
Mr	648.94	648.94
Dx,g cm-3	0.834	0.834
Z	2	2
Mu (mm-1)	0.383	0.383
F000	708.0	708.0
F000'	709.81	
h,k,lmax	12,14,26	12,14,26
Nref	9234	8913
Tmin,Tmax	0.887,0.933	0.489,1.000
Tmin'	0.865	

Correction method= # Reported T Limits: Tmin=0.489 Tmax=1.000 AbsCorr = MULTI-SCAN

Data completeness= 0.965 Theta(max)= 67.079

R(reflections)= 0.0755(6011) wR2(reflections)= 0.2531(8913)

S = 1.058 Npar= 489

The following ALERTS were generated. Each ALERT has the format
test-name_ALERT_alert-type_alert-level.
Click on the hyperlinks for more details of the test.

Alert level C

PLAT029_ALERT_3_C _diffrn_measured_fraction_theta_full value Low . 0.965 Why?
PLAT220_ALERT_2_C NonSolvent Resd 1 C Ueq(max)/Ueq(min) Range 3.5 Ratio
PLAT242_ALERT_2_C Low 'MainMol' Ueq as Compared to Neighbors of C23 Check

PLAT242_ALERT_2_C Low 'MainMol' Ueq as Compared to Neighbors of C27 Check
PLAT353_ALERT_3_C Long N-H (N0.87,N1.01A) N1 - H1 . 1.02 Ang.
PLAT420_ALERT_2_C D-H Without Acceptor N1 --H1 . Please Check
PLAT420_ALERT_2_C D-H Without Acceptor N2 --H2A . Please Check
PLAT906_ALERT_3_C Large K Value in the Analysis of Variance ...... 2.266 Check
PLAT911_ALERT_3_C Missing FCF Refl Between Thmin & STh/L= 0.597 318 Report

---

Alert level G

PLAT002_ALERT_2_G Number of Distance or Angle Restraints on AtSite 11 Note
PLAT003_ALERT_2_G Number of Uiso or Uij Restrained non-H Atoms ... 11 Report
PLAT007_ALERT_5_G Number of Unrefined Donor-H Atoms .... 2 Report
PLAT072_ALERT_2_G SHELXL First Parameter in WGHT Unusually Large 0.17 Report
PLAT154_ALERT_1_G The s.u.'s on the Cell Angles are Equal ..(Note) 0.003 Degree
PLAT172_ALERT_4_G The CIF-Embedded .res File Contains DFIX Records 8 Report
PLAT176_ALERT_4_G The CIF-Embedded .res File Contains SADI Records 3 Report
PLAT177_ALERT_4_G The CIF-Embedded .res File Contains DELU Records 6 Report
PLAT178_ALERT_4_G The CIF-Embedded .res File Contains SIMU Records 10 Report
PLAT199_ALERT_1_G Reported _cell_measurement_temperature ..... (K) 293 Check
PLAT200_ALERT_1_G Reported _diffrn_ambient_temperature ..... (K) 293 Check
PLAT300_ALERT_4_G Atom Site Occupancy of C28 Constrained at 0.5 Check

And 23 other PLAT300 Alerts

More ...

PLAT301_ALERT_3_G Main Residue Disorder ....(Resd 1) 6% Note
PLAT380_ALERT_4_G Incorrectly? Oriented X(sp2)-Methyl Moiety ..... C13 Check
PLAT412_ALERT_2_G Short Intra XH3 .. XHn H18 ..H29D . 2.07 Ang.
x,y,z = 1_555 Check
PLAT412_ALERT_2_G Short Intra XH3 .. XHn H22 ..H28F . 1.90 Ang.
x,y,z = 1_555 Check
PLAT606_ALERT_4_G Solvent Accessible VOID(S) in Structure .... ! Info
PLAT860_ALERT_3_G Number of Least-Squares Restraints .... 102 Note
PLAT909_ALERT_3_G Percentage of I>2sig(I) Data at Theta(Max) Still 44% Note
PLAT910_ALERT_3_G Missing # of FCF Reflection(s) Below Theta(Min). 4 Note
PLAT913_ALERT_3_G Missing # of Very Strong Reflections in FCF .... 1 Note
PLAT933_ALERT_2_G Number of OMIT Records in Embedded .res File ... 292 Note
PLAT941_ALERT_3_G Average HKL Measurement Multiplicity .... 2.9 Low
PLAT978_ALERT_2_G Number C-C Bonds with Positive Residual Density. 3 Info

---

0 **ALERT level A** = Most likely a serious problem - resolve or explain
0 **ALERT level B** = A potentially serious problem, consider carefully
9 **ALERT level C** = Check. Ensure it is not caused by an omission or oversight
47 **ALERT level G** = General information/check it is not something unexpected

3 ALERT type 1 CIF construction/syntax error, inconsistent or missing data
12 ALERT type 2 Indicator that the structure model may be wrong or deficient
10 ALERT type 3 Indicator that the structure quality may be low
30 ALERT type 4 Improvement, methodology, query or suggestion
1 ALERT type 5 Informative message, check

It is advisable to attempt to resolve as many as possible of the alerts in all categories. Often the minor alerts point to easily fixed oversights, errors and omissions in your CIF or refinement strategy, so attention to these fine details can be worthwhile. In order to resolve some of the more serious problems it may be necessary to carry out additional measurements or structure refinements. However, the purpose of your study may justify the reported deviations and the more serious of these should normally be commented upon in the discussion or experimental section of a paper or in the "special_details" fields of the CIF. checkCIF was carefully designed to identify outliers and unusual parameters, but every test has its limitations and alerts that are not important in a particular case may appear. Conversely, the absence of alerts does not guarantee there are no aspects of the results needing attention. It is up to the individual to critically assess their own results and, if necessary, seek expert advice.

Publication of your CIF in IUCr journals

A basic structural check has been run on your CIF. These basic checks will be run on all CIFs submitted for publication in IUCr journals (*Acta Crystallographica*, *Journal of Applied Crystallography*, *Journal of Synchrotron Radiation*); however, if you intend to submit to *Acta Crystallographica Section C* or *E* or

IUCrData, you should make sure that full publication checks are run on the final version of your CIF prior to submission.

Publication of your CIF in other journals

Please refer to the *Notes for Authors* of the relevant journal for any special instructions relating to CIF submission.

PLATON version of 18/09/2020; check.def file version of 20/08/2020

Datablock exp_1456 - ellipsoid plot

Download CIF editor (publCIF) from the IUCr
Download CIF editor (enCIFer) from the CCDC
Test a new CIF entry
