# Supplementary material for: Design of an open-shell nitrogen-centered diradicaloid with tunable stimuli-responsive electronic properties
Source: Commun Chem. 2022 Oct 14;5:127. doi: 10.1038/s42004-022-00747-8 (PMC9814612; doi:10.1038/s42004-022-00747-8)
Supplement: Supplementary file 1 — Shi_PR File [file 42004_2022_747_MOESM1_ESM.pdf]

nature portfolio

Peer Review File

Design of an open-shell nitrogen-centered diradicaloid with tunable stimuli-responsive electronic properties

Open Access This file is licensed under a Creative Commons Attribution 4.0 International License, which permits use, sharing, adaptation, distribution and reproduction in any medium or format, as long as you give appropriate credit to the original author(s) and the source, provide a link to the Creative Commons license, and indicate if changes were made. In the cases where the authors are anonymous, such as is the case for the reports of anonymous peer reviewers, author attribution should be to 'Anonymous Referee' followed by a clear attribution to the source work. The images or other third party material in this file are included in the article's Creative Commons license, unless indicated otherwise in a credit line to the material. If material is not included in the article's Creative Commons license and your intended use is not permitted by statutory regulation or exceeds the permitted use, you will need to obtain permission directly from the copyright holder. To view a copy of this license, visit http://creativecommons.org/licenses/by/4.0/.

This manuscript has been previously reviewed at another Nature Portfolio journal. This document only contains reviewer comments and rebuttal letters for versions considered at Communications Chemistry.

REVIEWERS' COMMENTS:

Reviewer #1 (Remarks to the Author):

The authors have addressed all the issues raised by the referees, and hence I can now recommend publication of this manuscript. I am satisfied with the authors' response and no further review is needed in my opinion.

Reviewer #2 (Remarks to the Author):

The current version of teh manuscript satisfies by previous concerns on this article. As such, the article is positively evaluated for publication in this Journal.

Reviewer #3 (Remarks to the Author):

The manuscript reports nitrogen-centered diradicaloid molecules stemming from nitrogen-containing phenoxazine dimers bridged via a double bond. The characterization of the compounds in the manuscript was acceptable and the paper was relatively well written. I served as reviewer on a previous draft of this manuscript submitted to another venue. The authors have improved the manuscript significantly from their original submission but there are still some minor errors and there are results that still raise some questions. For example, the authors have tried to address my concerns about the possibility of the cis isomer being energetically feasible and they now provide some calculated data but the data that they provided in the new draft shows the trans isomer being thermodynamically favored by only 0.17 kcal/mol. This is barely favorable (i.e. this difference in energy equates to an equilibrium ratio of about 1.3: 1 at ambient temperature) and does not explain why they only observe the trans in the solid state. Perhaps there are crystal packing effects. It is hard to tell from observing SI figures 9 and 10. It is suggested that the authors find some reasonable explanation of this observation (i.e. look more closely at the packing and see if there are electron-rich aryls pi-stacking with electron-poor rings or some other phenomenon).

In my original review, I asked what the phrase “diradical characteristics” meant (on line 411 of the new draft) and perhaps I was not blunt enough in my original review of what I really had a problem with. I thank the authors for interpreting my query so plainly by explaining diradical character in their rebuttal but this was completely unnecessary since I would not be qualified to review this manuscript if I was not already familiar with open-shell molecules. To say it plainly, I was actually trying to point out the poor English grammar and suggest that they change this phrase to read “diradical character” rather than “diradical characteristics.” The latter is not a phrase used ever to describe open-shell molecules.

The authors provide NOESY data in SI figure 1. The crosspeaks do not make sense for the labels that are provided. For example, it seems strange that the proton labeled "b" has a crosspeak with t-butyl labeled as "f" which is on the other side of the phenyl ring. I'm not sure what the authors are trying to show here. It's also strange, although not unprecedented, that the proton labeled as "a" does not show a crosspeak to both t-butyl groups. The authors are encouraged to review their NMR data carefully before publication.

Other than these small issues, I think the paper is nice work and warrants publication. It is still not as novel as they originally sold the paper as, but is certainly a great result. This venue is more appropriate for publication of this work once they make some minor edits/improvements.

Response to Reviewer 3:

The manuscript reports nitrogen-centered diradicaloid molecules stemming from nitrogen-containing phenoxazine dimers bridged via a double bond. The characterization of the compounds in the manuscript was acceptable and the paper was relatively well written. I served as reviewer on a previous draft of this manuscript submitted to another venue. The authors have improved the manuscript significantly from their original submission but there are still some minor errors and there are results that still raise some questions. For example, the authors have tried to address my concerns about the possibility of the cis isomer being energetically feasible and they now provide some calculated data but the data that they provided in the new draft shows the *trans* isomer being thermodynamically favored by only 0.17 kcal/mol. This is barely favorable (i.e. this difference in energy equates to an equilibrium ratio of about 1.3: 1 at ambient temperature) and does not explain why they only observe the *trans* in the solid state. Perhaps there are crystal packing effects. It is hard to tell from observing SI figures 9 and 10. It is suggested that the authors find some reasonable explanation of this observation (i.e. look more closely at the packing and see if there are electron-rich aryls pi-stacking with electron-poor rings or some other phenomenon).

In my original review, I asked what the phrase “diradical characteristics” meant (on line 411 of the new draft) and perhaps I was not blunt enough in my original review of what I really had a problem with. I thank the authors for interpreting my query so plainly by explaining diradical character in their rebuttal but this was completely unnecessary since I would not be qualified to review this manuscript if I was not already familiar with open-shell molecules. To say it plainly, I was actually trying to point out the poor English grammar and suggest that they change this phrase to read “diradical character” rather than “diradical characteristics.” The latter is not a phrase used ever to describe open-shell molecules.

The authors provide NOESY data in SI figure 1. The crosspeaks do not make sense for the labels that are provided. For example, it seems strange that the proton labeled "b" has a crosspeak with *t*-butyl labeled as "f" which is on the other side of the phenyl ring. I'm not sure what the authors are trying to show here. It's also strange, although not

unprecedented, that the proton labeled as "a" does not show a crosspeak to both t-butyl groups. The authors are encouraged to review their NMR data carefully before publication.

Other than these small issues, I think the paper is nice work and warrants publication. It is still not as novel as they originally sold the paper as, but is certainly a great result. This venue is more appropriate for publication of this work once they make some minor edits/improvements.

Reply: We greatly appreciate the reviewer's positive comments and meaningful advice in this manuscript.

First of all, as pointed out by the reviewer, **1a**'s preference for the *trans* isomer may be related to the intermolecular packing motif, since there was only a slight difference of 0.17 kcal/mol from the calculated energies of the two forms. As we have described in the manuscript (Fig. 4a), **1a** adopted 1D columnar packing along the *a* axis with offset face-to-face π–π stacking (~ 3.7 Å) and the adjacent π-stacks were connected through C−H · · · H−C interactions (~ 2.50 Å), which enhanced its conformation stability. Therefore, the crystal packing effects might also account for the favorable *trans* isomer observed in the solid state.

Secondly, we have understood the reviewer's consideration about the phrase "diradical characteristics", and we have replaced it with "diradical character" to describe our work more correctly.

Finally, we have corrected the labels of **1a** in its NOESY spectrum and variable temperature ^{1}H-NMR spectra according to the 1D and 2D NOESY spectra we have redone. Notably, due to the obvious NMR line broadening, the NOESY spectrum of **1a** was recorded at a low temperature (233 K). Specifically, we found the protons of *tert*-butyl labeled as "*g*" in higher field had NOE effect with both two protons of "*a*" and "*b*" in aromatic region, while the protons of *tert*-butyl labeled as "*f*" in lower field only exhibited NOE effect with aromatic proton "*a*" according to the 1D NOESY spectrum. The very weak crosspeak between *t*-butyl "*g*" and proton "*a*" in 2D NOESY spectrum of **1a** was mainly due to its intrinsic paramagnetic broadening. Therefore, the unambiguous assignment of the ^{1}H NMR spectrum of **1a** can be made based upon a

combination of 1D and 2D NOESY techniques. The experiment result had added into the supplementary Fig. 1.

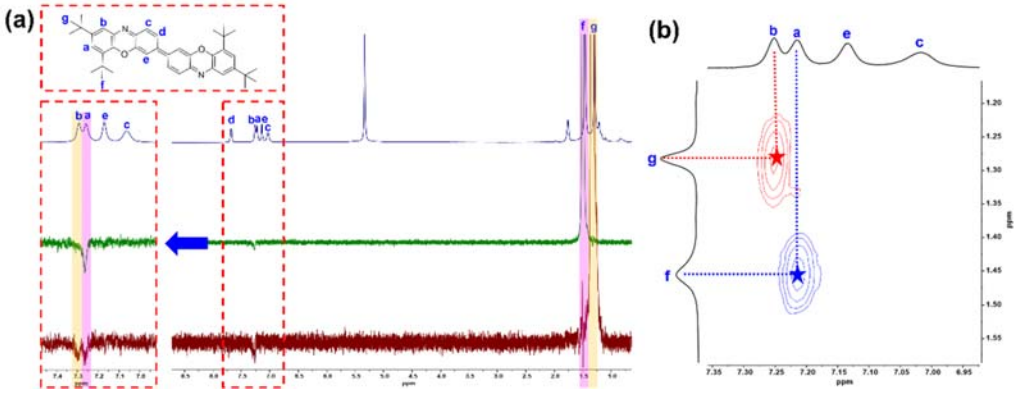

Supplementary Fig. 1: (a) 1D NOESY NMR (500 MHz, CD_2Cl_2, 233 K) spectrum and (b) 2D NOESY NMR (600 MHz, CD_2Cl_2, 233 K) spectrum of **1a**.
